# Intestinal miRNAs regulated in response to dietary lipids

**DOI:** 10.1038/s41598-020-75751-w

**Published:** 2020-11-03

**Authors:** Judit Gil-Zamorano, João Tomé-Carneiro, María-Carmen Lopez de las Hazas, Lorena del Pozo-Acebo, M. Carmen Crespo, Diego Gómez-Coronado, Luis A. Chapado, Emilio Herrera, María-Jesús Latasa, María Belén Ruiz-Roso, Mónica Castro-Camarero, Olivier Briand, Alberto Dávalos

**Affiliations:** 1 Laboratory of Epigenetics of Lipid Metabolism, Madrid Institute for Advanced Studies Food (IMDEA Food), CEI UAM + CSIC, Carretera de Canto Blanco, 8, 28049 Madrid, Spain; 2 Laboratory of Functional Foods, Madrid Institute for Advanced Studies Food (IMDEA Food), CEI UAM CSIC, 28049 Madrid, Spain; 3 Servicio de Bioquímica-Investigación, Hospital Universitario Ramón y Cajal, IRYCIS, 28034 Madrid, Spain; 4 Centre of Biomedical Research in Physiopathology of Obesity and Nutrition (CIBEROBN), Instituto de Salud Carlos III, 28029 Madrid, Spain; 5 Department of Biochemistry and Chemistry, Faculties of Pharmacy and Medicine, Universidad San Pablo CEU, 28668 Madrid, Spain; 6 Servicio de Cirugía Experimental, Hospital Universitario Ramón y Cajal, IRYCIS, 28034 Madrid, Spain; 7 Univ. Lille, Inserm, CHU Lille, Institut Pasteur de Lille, U1011-EGID, Lille, 59000 France

**Keywords:** Gastrointestinal models, Metabolism, Non-coding RNAs, Lipids, RNA

## Abstract

The role of miRNAs in intestinal lipid metabolism is poorly described. The small intestine is constantly exposed to high amounts of dietary lipids, and it is under conditions of stress that the functions of miRNAs become especially pronounced. Approaches consisting in either a chronic exposure to cholesterol and triglyceride rich diets (for several days or weeks) or an acute lipid challenge were employed in the search for intestinal miRNAs with a potential role in lipid metabolism regulation. According to our results, changes in miRNA expression in response to fat ingestion are dependent on factors such as time upon exposure, gender and small intestine section. Classic and recent intestinal in vitro models (i.e. differentiated Caco-2 cells and murine organoids) partially mirror miRNA modulation in response to lipid challenges in vivo. Moreover, intestinal miRNAs might play a role in triglyceride absorption and produce changes in lipid accumulation in intestinal tissues as seen in a generated intestinal *Dicer1*-deletion murine model. Overall, despite some variability between the different experimental cohorts and in vitro models, results show that some miRNAs analysed here are modulated in response to dietary lipids, hence likely to participate in the regulation of lipid metabolism, and call for further research.

## Introduction

The intestine plays an important regulatory role in whole-body lipid homeostasis^1^. In the intestine, cholesterol and triglycerides are synthesized endogenously, and dietary lipids are absorbed and packed into chylomicrons for release into circulation in a multifactorial-dependent regulation^2^. Dysregulation of this process can contribute to dyslipidemias which are characteristic of metabolic disorders and cardiovascular disease^3^. Despite the great advances made on the prevention and treatment of dyslipidemias, the development of therapies with enhanced efficacy is still needed^4^.

MicroRNAs (miRNAs) are 18–24 nucleotide-long key elements in gene expression modulation at the posttranscriptional level, exerting important biological effects both intra- and extra-cellularly. miRNAs usually bind to several target mRNAs and one mRNA transcript can be targeted by multiple miRNAs^5^. Moreover, miRNAs are important for the maintenance of homeostatic conditions and their dysregulation may reflect pathophysiological changes^6^. Due to the relevant role miRNAs have in these processes their use as potential therapeutic tools in several diseases is being investigated^7^. Increasing evidence suggests that miRNAs can be therapeutically modulated by the diet^8^.

The processing of precursor miRNAs to form most mature miRNAs is indispensably dependent on the RNase III endonuclease Dicer^9^. Dicer deletion in cell/tissue-type specific studies has shown important roles for miRNAs in vascular development and atherosclerosis^10–12^, neurological development and functionality^13^, and obesity^14^, among others. The role of miRNAs in the regulation of cholesterol and lipid metabolism in different tissues has been established previously; however, their role in the intestine is much less described. Thus, further clarification is needed regarding the miRNAs acting in the intestinal compartment, along with their target genes and the regulatory networks that are affected.

Tissue specific Dicer KO models have been used in some studies to assess the importance of miRNAs in intestinal homeostasis^15–18^, as well as their involvement in lipid metabolism^19–21^. While the primary role of miRNAs seems to be the ‘fine-tuning’ of gene expression, it is under conditions of stress that the functions of miRNAs become especially pronounced, underscoring their roles in disease^22^. Modern dietary patterns, with highly palatable and energetic foods, rich in simple carbohydrates and lipids, contribute to dietary excess-related diseases. In this context, the intestine must cope with the high amount of lipid consumed, which might represent a “stress condition”. Therefore, it is likely that exposing the intestines to dietary lipids will underscore novel miRNAs associated with the regulation of different aspects of intestinal lipid metabolism. Given the above, the purpose of the present study was to search for novel miRNAs with a potentially relevant role in intestinal lipid metabolism. Moreover, an intestine-specific *Dicer1* KO mouse model was used to address the importance of intestinal miRNAs in gut lipid metabolism.

## Results

### Screening for intestinal miRNAs responsive to acute and chronic lipid challenges

**Figure 1:**
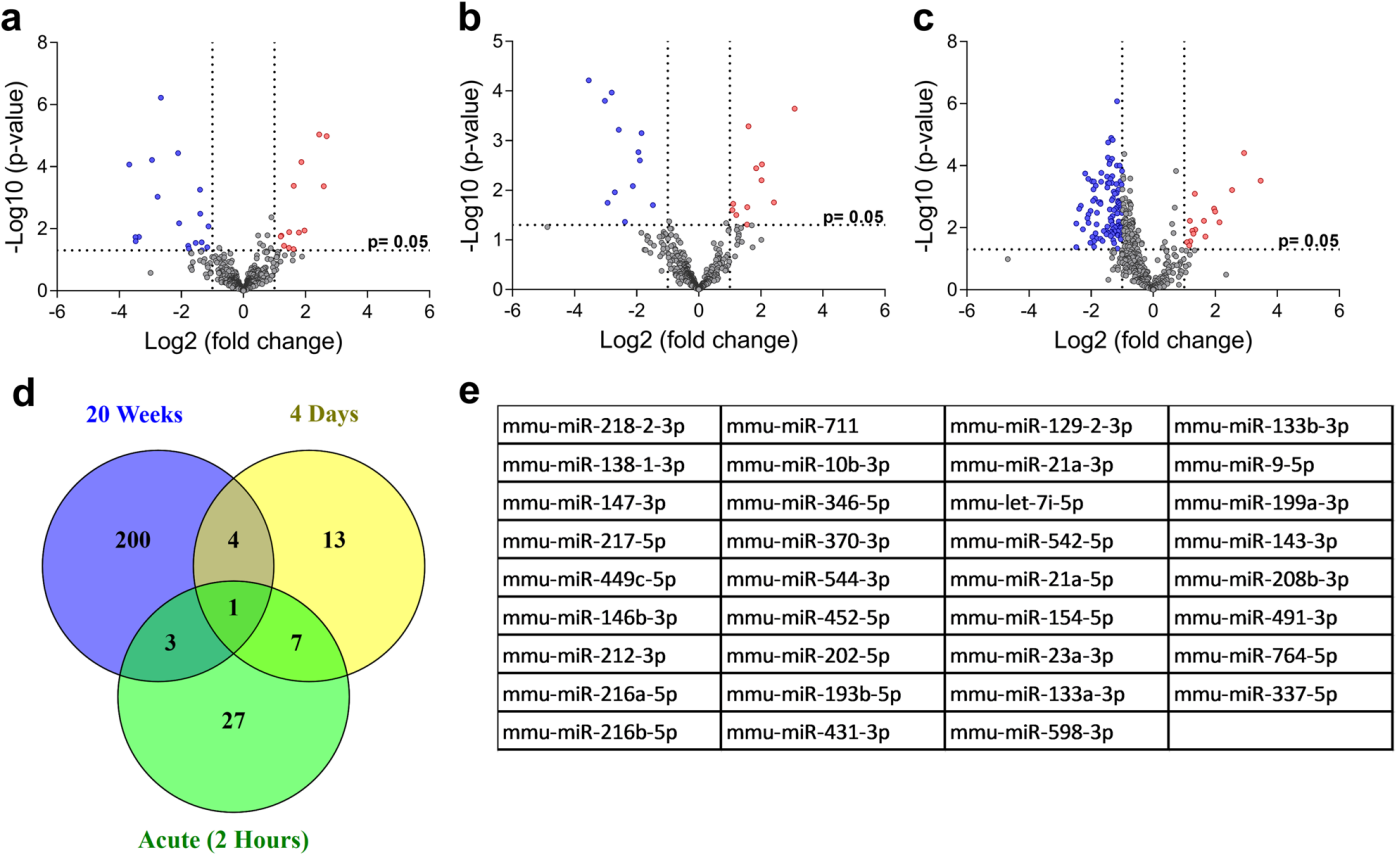
Differentially expressed miRNAs found (**a**) 2 h after an oral administration of a cholesterol-enriched (40 mg) olive oil (250 µL) solution (acute), (**b**) 4 days after the consumption of a high-fat diet (HFD), and (**c**) 20 weeks after HFD, in the small intestine (whole) of male C57BL/6 mice (n ≥ 4 per group). Up- and down-regulated miRNAs are shown as red and blue dots, respectively. (**d**) Venn diagram represents the number of common differently expressed miRNAs in *acute*, 4 days and 20 weeks studies. mmu-miR-218-2-3p was common among the three studies; mmu-miR-147-3p, -138-1-3p, -1894-3p and -666-3p were common between the two chronic consumption studies; mmu-miR-449c-5p, -1894‐5p and ‐217‐5p were shared by the 20 weeks and acute experiments; mmu‐miR‐879‐3p, ‐711, ‐146b‐3p, ‐216b‐5p, ‐212‐3p, ‐216a‐5p and ‐291a-5p were common to the 4 days and acute studies. (**e**) List of 35 miRNAs selected for further validation.

Since stressful conditions, such as dietary lipid challenges, can underscore miRNAs function, three sets of experimental settings (one acute and two chronic) were carried out with male C57BL/6 mice. In the case of the acute study, the expression of miRNAs in the small intestine was investigated 2 h after the administration of a sole dose of a cholesterol-enriched olive oil solution. On the other hand, chronic approaches involved the consumption of HFD for 4 days or 20 weeks, followed by the assessment of miRNA expression in the small intestine in each case. Numerous differently expressed miRNAs were found in *acute* (Fig. 1a, Supplementary Table S1), 4 days (Fig. 1b, Supplementary Table S2) and 20 weeks (Fig. 1c, Supplementary Table S3) studies.

Next, a list of promising miRNAs as important role players in intestinal lipid metabolism was selected for validation. One selection criterium consisted in miRNAs common to at least 2 experimental settings (Fig. 1d). Among the differentially expressed miRNAs found, mmu-miR-218-2-3p was common to all three experimental settings. Four miRNAs were shared between the chronic consumption studies, namely mmu‐miR‐147‐3p, ‐138‐1‐3p, ‐1894‐3p and ‐666‐3p. In addition, the *acute* experiment showed mutual miRNAs with 20 weeks (mmu‐miR‐449c‐5p, ‐1894‐5p and ‐217‐5p) and 4 days (mmu‐miR‐879‐3p, ‐711, ‐146b‐3p, ‐216b‐5p, ‐212‐3p, ‐216a‐5p and ‐291a-5p) experiments. Additionally, miRNAs showing the highest fold changes in each study were also selected for validation. Furthermore, any miRNAs without previously reported human counterparts were excluded. Finally, 35 miRNAs were selected for further validation (Fig. 1e).

### Intestinal miRNAs expression in response to dietary lipids is influenced by different factors

#### Time-course response upon exposure to dietary lipids

**Figure 2:**
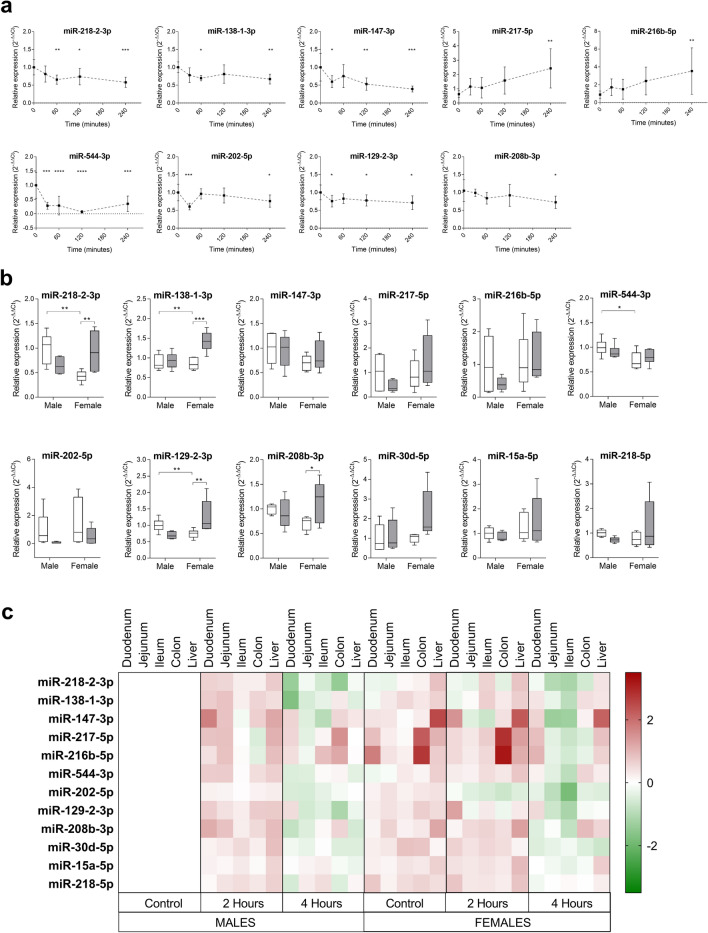
(**a**) Time-course (30, 60, 120 and 240 min) expression of miRNAs showing significant differences in response to an oral lipid challenge in male C57BL/6 mice. Data are shown as mean ± SD; *n* ≥ 7. *p < 0.05, **p < 0.001, ***p < 0.0001, compared with t = 0 (control). (**b**) Relative expression of miRNAs determined 2 h after an oral lipid challenge given orally to male and female wild-type mice (grey), compared to controls (white); *n* ≥ 10 per group. Two-way ANOVA was followed by Bonferroni’s post-hoc tests for multiple comparisons. *p < 0.05, **p < 0.01, ***p < 0.001. (**c**) Heatmap of differentially expressed intestinal and hepatic miRNAs 2 and 4 h after an oral lipid challenge compared to controls (male mice); *n* ≥ 8 per group. Up- and down-regulated miRNAs compared to controls are represented in red and green, respectively.

Some of the previously selected miRNAs (see above) may play a role in the acute intestinal response to dietary lipids. Thus, a kinetic study of up to 4 h upon the exposure to a lipid challenge [cholesterol-enriched (40 mg) olive oil (250 µL)] was performed to assess if and how miRNA levels of expression change with time in the small intestine. Upon the lipid challenge, 9 miRNAs showed statistically significant expression changes in, at least, one time point compared to basal levels (t = 0) (Fig. 2a), whereas the other 26 did not show significant changes (Supplementary Fig. S1).

#### Sex influences intestinal miRNAs expression in response to dietary lipids

Selected miRNAs were evaluated in a cohort of WT mice (*n* > 20 per group) to assess sex influence in intestinal miRNAs expression in the small intestine in response to dietary lipids. A large variability was found when intestinal miRNAs expression data was analysed without gender separation (data not shown). However, when the analysis was divided by gender, a third of miRNAs (miR-218-2-3p, -544-3p, -129-2-3p and -138-1-3p) showed statistically significant differences of expression between male and female controls (Fig. 2b). In addition, significant differences were seen for miR-218-2-3p, -208b-3p, -129-2-3p and -138-1-3p in female mice fed with a HFD compared to controls, whereas miRNAs levels were not significantly affected in males. Overall, results suggest that, in response to a HFD, intestinal miRNAs levels are influenced by animal gender and that the changes in the expression of certain miRNAs are enhanced in females.

#### miRNAs levels found in the small intestine of HFD-fed mice vary depending on the intestinal segment analysed

The small intestine has certain polarity along the whole tissue; thus, it is conceivable that miRNA expression is not identical between the different sections of the small intestine. As seen above, changes in miRNAs levels as a response to a lipid challenge may occur at specific time points and be transient (see first sub-section). Moreover, gender differences seem to influence miRNA expression in response to dietary lipids (see previous sub-section). Given the above, the expression levels of selected miRNAs were assessed, at different time points, in the duodenum, jejunum and ileum of mice (*n* ≥ 8 per group) receiving an oral lipid challenge, and a gender-divided analysis was conducted. With the purpose of widening the scope of examination, colon and liver miRNA expression was also assessed.

As expected, miRNAs expression changes depended, at least partially, on small intestine section, gender and time after exposure to the lipid challenge (Fig. 2c, Supplementary Fig. S2). For example, in males, miR-218-2-3p expression in the duodenum and jejunum is significantly increased at 2 h upon exposure, returning to basal values after 4 h, whereas expression levels remain constant in females. Interestingly, the expression patterns of this miRNA in ileum seem to be reversed in what comes to gender, i.e. there is a significantly increase at 2 h upon exposure in females, which returns to basal levels after 4 h, whereas expression levels remain constant in males. Other examples worth referring include miR-129–2-3p, -138-1-3p and -147-3p. In males, 2 h upon the lipid challenge miR-138-1-3p levels were found to be significantly raised compared to control in jejunum. At 4 h post-lipid challenge, however, dropped compared to control, reaching statistical significance in the duodenum of males and in the ileum of females. miR-129-2-3p levels were found to be raised compared to control in duodenum of females, 2 h upon the lipid challenge. Yet, at 4 h post-lipid challenge, miR-129-2-3p levels dropped significantly in the jejunum and ileum of females. Finally, miR-147-3p levels were found to be raised in duodenum (reaching statistical significance) and jejunum in males, 2 h after the lipid challenge. However, 4 h post-lipid challenge levels dropped to control levels.

In response to the lipid challenge, the expression of some miRNAs was also significantly altered in a gender- and time-dependent way both in liver (miR-30d-5p, -129-2-3p, -147-3p and -202-5p) and colon (miR-30d-5p, -129-2-3p, -202-5p, -216b-5p-3p, -217-5p and -218-2-3p) (Fig. 2c, Supplementary Fig. S3).

### In vitro intestinal models partially mirror miRNA expression changes in response to lipid challenges in vivo

#### Caco-2 as a classic model of the intestinal epithelium

**Figure 3:**
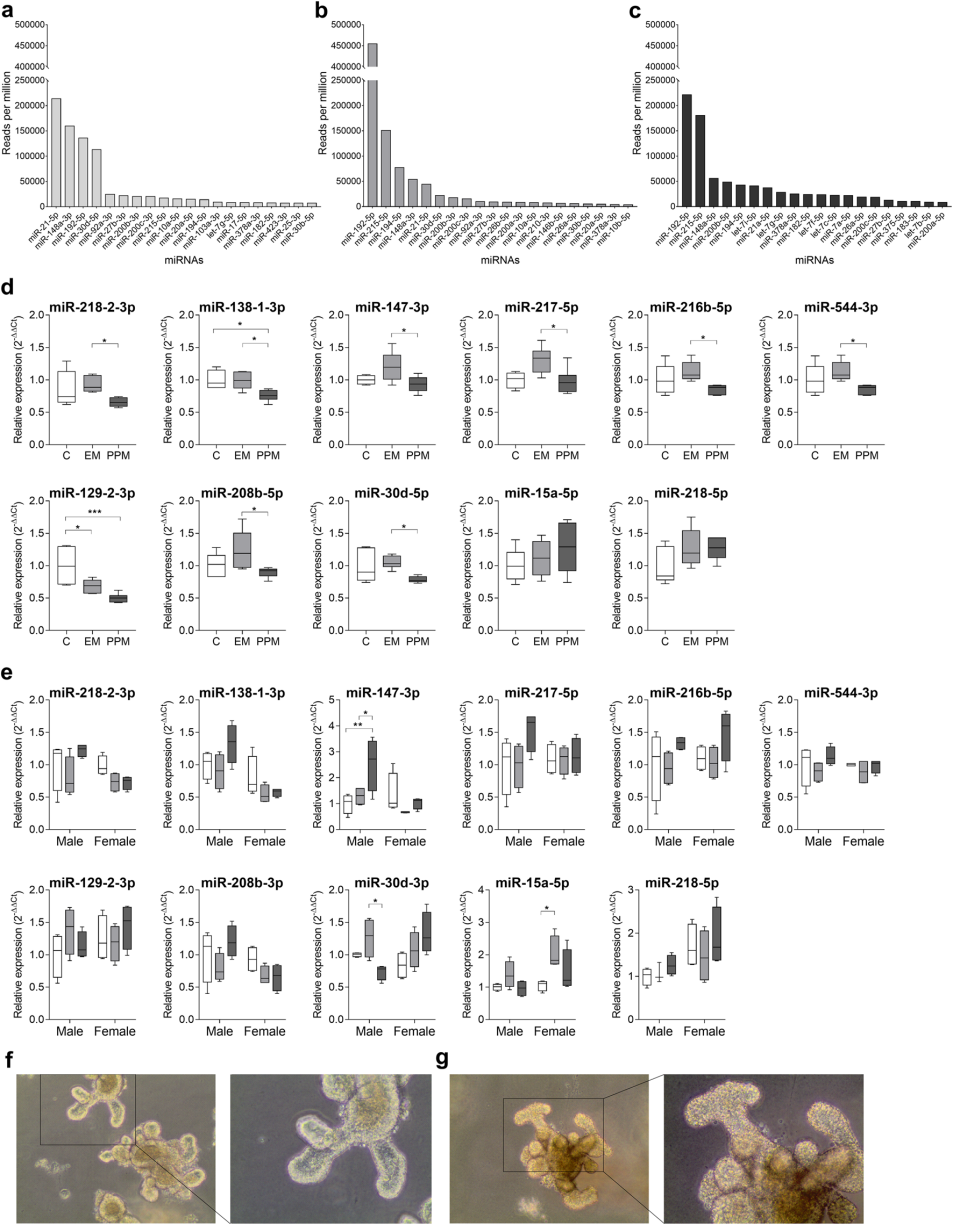
The twenty most abundant miRNAs, represented as reads per million, found in undifferentiated (**a**) and differentiated (**b**) Caco-2 cells, and in enteroids (isolated from male and female C57BL/6 mice) exposed to postprandial micelles (PPM) for 24 h (**c**). (**d**) Relative expression levels of selected miRNAs in differentiated Caco-2 cells exposed to DMEM (controls), empty micelles (EM) or PPM, for 24 h; *n* = 6 per group. One-way ANOVA was followed by Bonferroni’s post-hoc tests for multiple comparisons. *p < 0.05, **p < 0.01, ***p < 0.001. (**e**) Relative expression of selected miRNAs in intestinal organoids exposed to DMEM (controls; white), EM (grey) or PPM (dark grey), for 24 h; *n* = 4 per group. Two-way ANOVA was followed by Bonferroni’s post-hoc tests for multiple comparisons. *p < 0.05, **p < 0.01. (**f**,**g**) Light microscope representative images of mature enteroids treated with empty (**f**) or post-prandial (**g**) micelles (for each case, image on the left (10 ×) is zoomed on the right (20 ×)).

First, small RNA-Seq was used to compare miRNA expression between undifferentiated and differentiated Caco-2 cells (Fig. 3a,b). Although, some miRNAs characteristically expressed in the intestine, such as the let-7 family, were not present in either case, the reads of other typical intestinal miRNAs (miR-192, -215, -194, -30d, -148a-3p and -21-5p) in differentiated Caco-2 cells were similar to the ones found in the small intestine. Next, differentiated Caco-2 cells were exposed to DMEM (controls), empty micelles (EM) or postprandial lipid micelles (PPM), for 24 h. As was the case for enteroids (see below) expression levels of selected miRNAs were assessed to confirm their response upon the exposure to lipid micelles (Fig. 3d). Statistical significance between control and PPM groups was seen for miR-129-2-3p and miR-138-1-3p. In the latter case, significantly statistical differences were also present between PPM and EM groups.

#### Enteroids as a recent model of the intestinal epithelium

Here, enteroids were exposed to postprandial lipid micelles for 24 h and small RNAs were analysed by RNA-seq. Highly expressed miRNAs included miR-192, miR-215, miR-148 and the let-7 family (Fig. 3c), which have all been reported to be characteristic of the small intestine. Previous work in our lab, showed that 3 miRNAs responded significantly (p < 0.05) to the lipid challenge (PPM) compared to controls, namely mmu-miR-30d-5p, -15a-5p and -218-5p (unpublished data). Thus, here, these miRNAs were assessed in conjunction with the other miRNAs showing significant differential expression in response to an acute lipid challenge (see previous section). Interestingly, significant gender-specific expression patterns were found for miR-15a-5p, -30d-5p and -147-3p in response to the lipid challenge (Fig. 3e).

### Bioinformatic analysis of miRNAs targets

**Figure 4:**
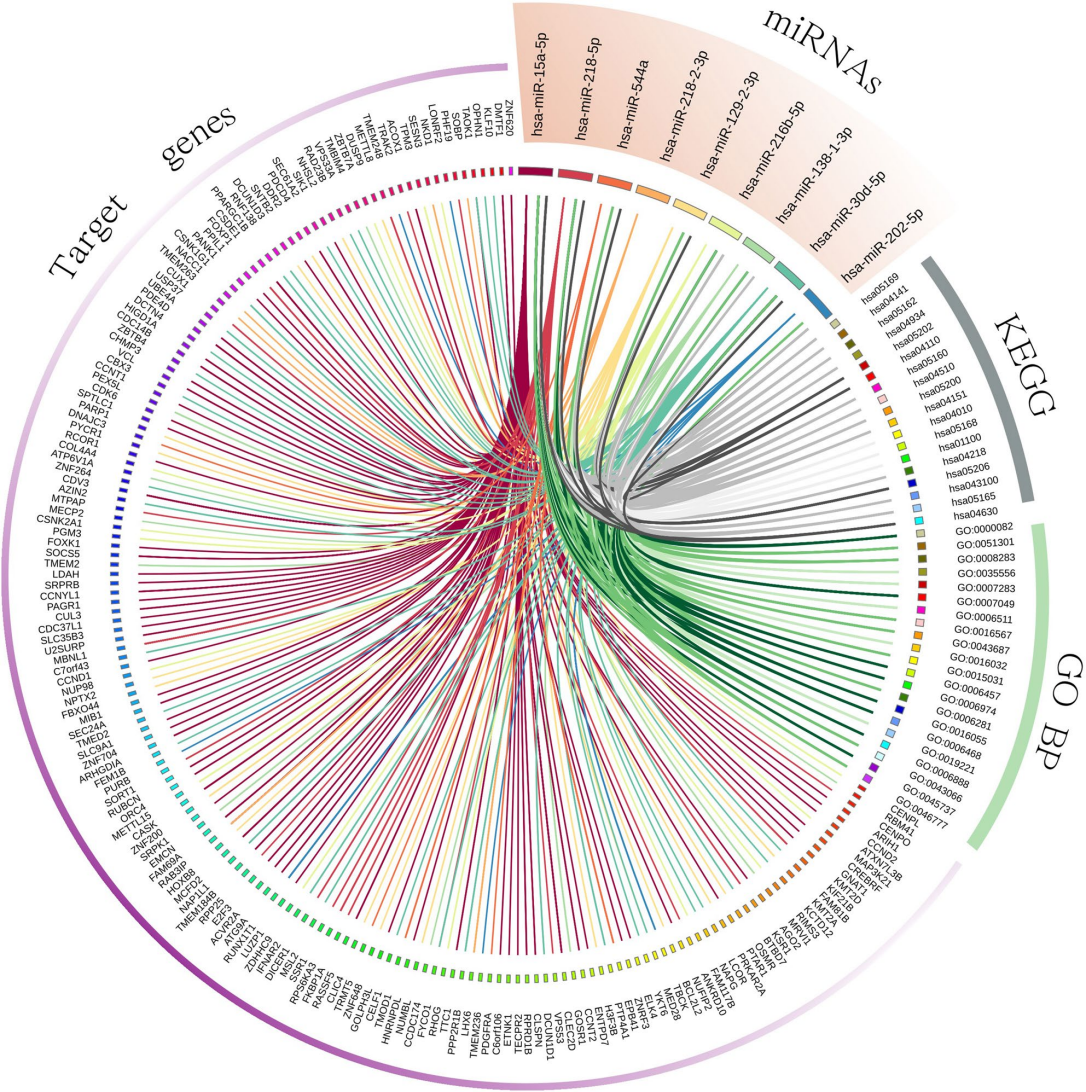
CIRCOS graphical representation of relationships among each selected miRNA with their validated target genes and the pathways involved. Different color lines relate each miRNA to genes for which there is an experimentally validated interaction. Lines between each pathway (green for KEGG and grey for GO pathways) and a miRNA imply a relationship based on the latter target genes. The different colour gradation in the pathway lines (green and grey for KEGG and GO, respectively) indicate the adjusted p-value: dark (p-value < 0.05), medium (p-value 0.05–0.01) and light (p-value > 0.01).

To assess possible targets and pathways involving the 12 selected miRNAs, a bioinformatics analysis was performed. One hundred and eighty different gene targets were obtained, focusing only on experimentally verified miRNA-target interactions (using miRTarBase entries that have been validated experimentally). A set of pathways in which miRNAs may be involved was identified by gene set enrichment analysis (GSEA) (Fig. 4, Supplementary Table S4). For example, according to the Kyoto Encyclopedia of Genes and Genomes (KEEG), significant pathways identified included cell cycle (hsa04110), JAK-STAT signalling pathway (hsa04630), microRNAs in cancer (hsa05206) and transcriptional misregulation in cancer (hsa05202). As for the Gene Ontology (GO) enrichment analysis the following identified pathways were significantly affected: positive regulation of cyclin-dependent protein serine-threonine kinase activity (GO: 0045737), protein folding (GO: 0006457); cell cycle (GO: 0007049), Wnt signalling pathway (GO: 0016055), G1S transition of mitotic cell cycle (GO: 0000082), ER to Golgi vesicle-mediated transport (GO: 0006888), protein phosphorylation (GO: 0006468) and cellular response to DNA damage stimulus (GO: 0006974).

### Loss of intestinal *Dicer1* influences lipid absorption

**Figure 5:**
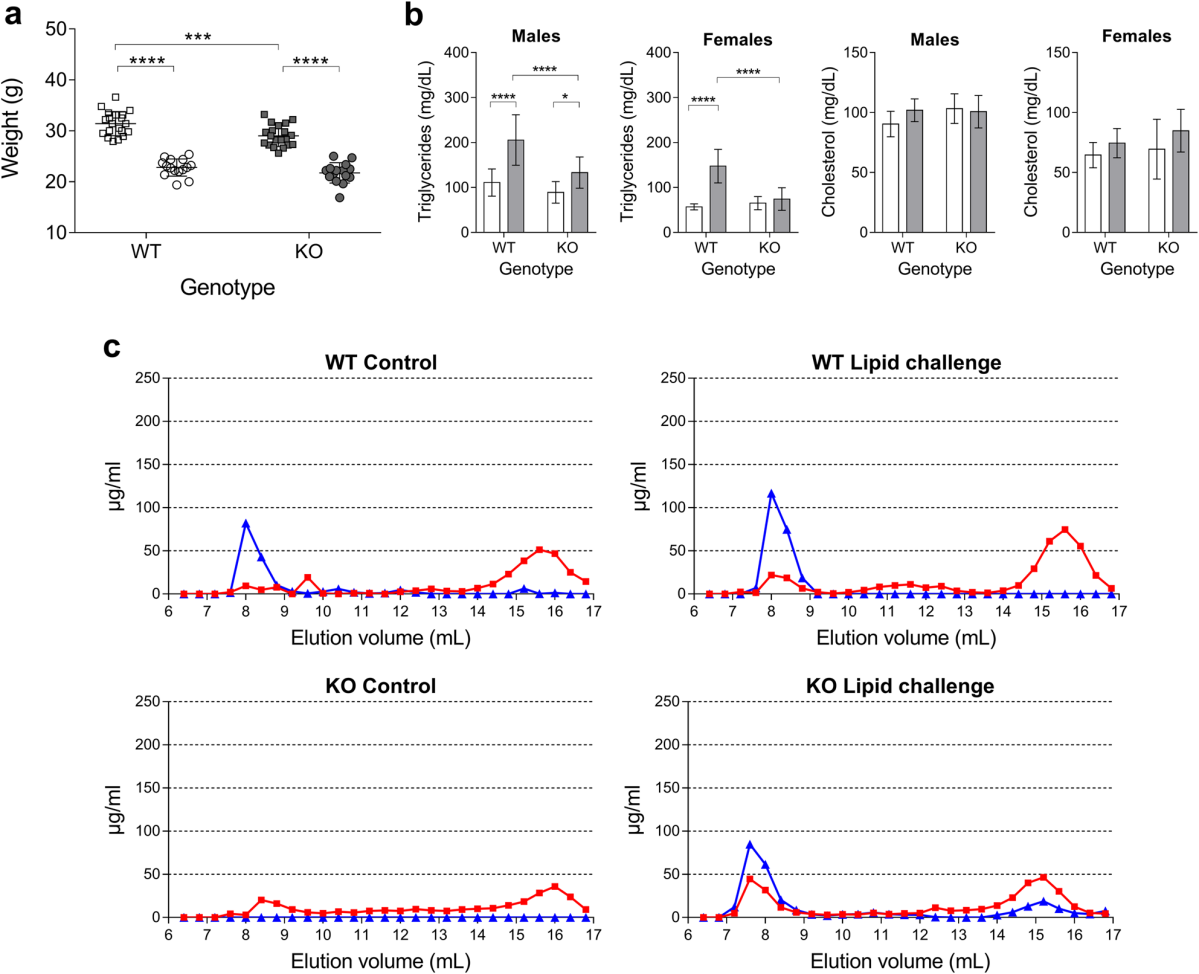
(**a**) Males (squares) and females (circles) weight (g) for WT (*Dicer1*^loxP/loxP^, Vil-cre(−)) and KO (*Dicer1*^loxP/loxP^, Vil-cre(+)) mice; *n* ≥ 15 per group. Corresponding means and SD bars are shown for each group. Two-way ANOVA was followed by Bonferroni’s post-hoc tests for multiple comparisons. *p < 0.05, **p < 0.01, ***p < 0.001, ****p < 0.0001. (**b**) Plasma triglycerides and cholesterol levels 2 h after an oral administration of a cholesterol-enriched olive oil solution (grey), compared to controls (water; white); *n* ≥ 10 per group. Data are represented as mean ± SD. Two-way ANOVA was followed by Bonferroni’s post-hoc tests for multiple comparisons. p < 0.05, **p < 0.01, ***p < 0.001, ****p < 0.0001. (**c**) Plasma cholesterol (red) and triglycerides (blue) FPLC profile in WT and *Dicer1* KO mice submitted to an oral lipid challenge or water (controls), *n* = 4 per group.

Since loss of Dicer studies can provide valuable information regarding the potential relevance of miRNAs in intestinal lipid metabolism, here, an intestinal *Dicer1* KO mice model was first generated. A significant difference in mean weight was seen between WT and KO males in favor of the former (Fig. 5a), whereas females showed non-significant weight differences between groups. In addition, 2 h after the oral administration of a lipid challenge, triglyceride levels were significantly lower in KO mice compared to WT, both in males and females (Fig. 5b), whereas cholesterol levels remained constant in both genotypes and genders. In control conditions, *Dicer1* KO mice hardly present VLDL, whereas after the lipid challenge, they show VLDL with lower triglyceride (TG) content and HDL with lower cholesterol content compared to WT mice (Fig. 5c).

### Changes in lipid accumulation in animals that lack intestinal *Dicer1*

**Figure 6:**
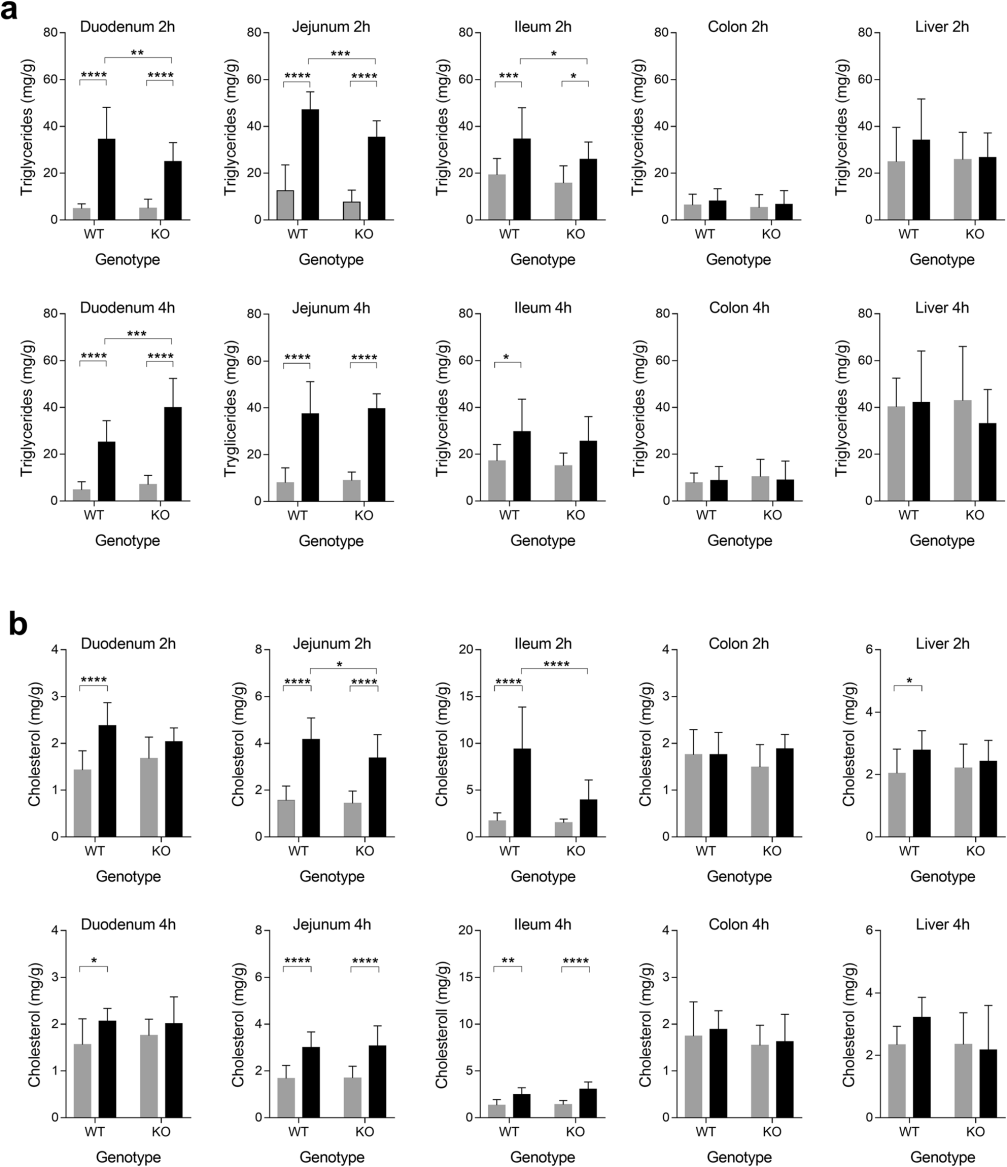
Intestinal and hepatic triglyceride (**a**) and cholesterol (**b**) levels (mg/g) found in WT (*Dicer1*^loxP/loxP^, Vil-cre(−)) and KO (*Dicer1*^loxP/loxP^, Vil-cre(+)) mice, 2 and 4 h after the administration of cholesterol-enriched olive oil (black) or water (grey); *n* ≥ 9. Data are represented as mean ± SD. Two-way ANOVA was followed by Bonferroni’s post-hoc tests for multiple comparisons. *p < 0.05, **p < 0.01, ***p < 0.001, ****p < 0.0001.

Lipid accumulation was analysed in different sections of the small intestine of *Dicer1* deficient mice, using colon and liver as controls (Fig. 6). In all three sections of the small intestine, triglyceride levels were lower 2 h after the lipid challenge (Fig. 6a) in *Dicer1* KO mice compared to their control littermates. By contrast, 4 h post lipid challenge, increased levels of triglycerides were found in the duodenum. Moreover, 2 h post challenge, lower levels of cholesterol were found in the duodenum and jejunum of *Dicer1* KO mice (Fig. 6b). Statistically significant changes between genotypes were not found in phospholipid levels (Supplementary Fig. S4). Overall, data suggest that the loss of *Dicer1* in the intestine may induce a delay in lipid absorption, as observed at 2 h post challenge, and an increase in lipid accumulation, as detected 4 h post challenge, in specific parts of the intestine.

## Discussion

miRNAs are involved in a multitude of cellular processes and intestinal responses may also be reflected in changes in the profile of miRNAs. Many studies have already demonstrated the importance of microRNAs in the maintenance of intestinal homeostasis, affecting the architecture and functionality of the epithelium itself and also the interaction/communication between the immune system and the microbiota^23^. The intestinal microbiome can influence host metabolic phenotypes and miRNAs are involved in the host gene regulation produced by the microbiota^24,25^. Intestinal epithelial cells, Paneth cells and globet cells are the main sources of fecal miRNAs, which can affect bacterial growth and the composition of the intestinal microbiota^16^. Furthermore, miRNAs have been described to regulate lipid metabolism in different tissues, including adipose, liver, brain or the vasculature. However, their role in intestinal lipid metabolism is poorly described. Here, the focus was set on the regulation of microRNAs in the intestine as a fundamental organ in lipid absorption and metabolism.

The functions of miRNAs become especially pronounced under stress conditions. The small intestine is constantly exposed to high amounts of dietary lipids, which could be considered as a stress condition. In this sense, alterations in the intestinal epithelium were already observed after 3 days of HFD, as saturated fats result in increased intestinal villi height in the jejunum and ileum of animals who underwent intestinal resection^26^. Fat can influence nutrient absorption and the protective function of the mucosal barrier. Adaptive changes occur in mice enterocytes in short periods of time, modulating gene expression related to glucose and lipid metabolism in the duodenum and jejunum^27^. Also, the intestines of animals fed with HFD, for 2, 4 and 8 weeks, show a differential expression of genes that intervene in lipid metabolism, cell cycle and inflammation. Furthermore, a differential expression in genes associated with PPARα regulated lipid metabolism genes was observed with 20–30% fat diets, whereas in diets with 45% fat differentially expressed genes were related to alterations in cell cycle and immunity^28,29^.

Here, the profile of intestinal miRNAs was investigated in murine models submitted to a stress condition caused by dietary lipid challenges in the form of HFD (chronic) or acute high fat solution administrations by oral gavage. In a previous study, we explored how the reduction in miRNA-mediated regulation, observed in the same *Dicer1* KO model used here, could affect intestinal lipid metabolism after an acute lipid challenge, by investigating cholesterol and lipid metabolism-related genes potentially modulated by intestinal miRNAs^21^. In that study, KO mice showed a decreased expression of markers related to the integrity of the intestinal epithelium, which could be related to an alteration in lipid metabolism and the process of ketogenesis. Hmgcs2, Acat1 and Orl1 gene expression was significantly affected in *Dicer1* KO mice compared to WT and this was accompanied by the repression of five miRNAs capable of targeting all three genes. Here, the list of differently expressed miRNAs, common to both acute and chronic experimental settings, included none of these miRNAs. In addition, the kinetics accompanying the expression changes observed and their potential gender- and intestinal section- dependence were also explored here. Importantly, genetic analyses were complemented with lipid analysis, including the determination of plasma lipid and lipoprotein profiles and intestinal and hepatic lipid levels.

Several differentially expressed miRNAs were found, yet miR-218-2-3p was the single miRNA common to both chronic and acute experimental settings. miR-218-2-3p expression was significantly increased at 2 h upon exposure to a lipid challenge in the duodenum and jejunum of male mice, and in the ileum of females. Recently, via loss and gain of function experiments, Bresciani et al. showed that miRNA-218 targets the lipin-1 gene altering its functionality^30^. Members of the Lipin family have a critical role in the regulation of intestinal lipid homeostasis and chylomicron production^31^. Nevertheless, the lipin gene family was not found amongst the list of genes with validated interactions (3′ UTR and binding probability > 0.95) for the selected miRNAs.

The reduction seen in the levels of miR-138-1-3p in the 20 weeks study and in the duodenum of males and ileum of females 4 h post-lipid challenge compared to controls is in line with the results seen in Caco-2 cells after 24 h of exposure to postprandial micelles. On the other hand, miR-138-1-3p levels were increased in the 4 days study, as well as in females and in the jejunum of males, 2 h upon the lipid challenge. miR‐138 has been reported to modulate osteogenic mesenchymal stem cell differentiation^32^ and its ectopic expression increases the generation of pluripotent stem cells through a decrease in p53^33^. To the best of our knowledge, it has not been associated to lipid metabolism.

As for miR-129-2-3p, a reduction in its levels was seen in the 4 days experiment and kinetics studies, as well as in the jejunum and ileum of female mice and in the colon of both genders, which also agrees with what was seen in Caco-2 cells. However, 2 h upon the lipid challenge, a statistically significant rise in the levels of miR-129-2-3p compared to controls was found in the duodenum of females. As for miR-138, most literature on miR‐129‐2‐3p refers to oncology studies. In addition, miR‐129‐2‐3p was associated with the risk of ischemic stroke^34^ and it has also been suggested that it is related with inflammatory processes^35^.

Compared to controls, the levels of miR-147-3p were found to be raised after the lipid challenge both in enteroids isolated from male mice and in the duodenum of males. However, this miR showed a decrease in expression levels in the kinetics experiment and in the 20 weeks and 4 days studies (compared to controls). MiR‐147 has mainly been described as an anti-inflammatory miR^36,37^ and has also been reported in oncology research^38,39^. To the best of our knowledge it has never been associated with a potential role in the intestinal regulation of lipid metabolism.

The integrative analysis of the studied miRNAs shows that the possible target genes of these miRNAs are related in a series of pathways. These pathways are involved in the maintenance of intestinal homeostasis, adaptive changes and/or inflammatory processes. This, as previously noted, would occur in response to a high-fat diet. For example, it is known that Wnt signalling components regulate a complex set of cellular responses by activating genes, ion channels and cytoskeletal rearrangements that regulate polarity, proliferation and maturation of cells in the gut. Therefore, the Wnt signalling pathway is critical to establish and maintain the proliferative capacity. This allows the self-renewal of intestinal cells but is also involved in carcinogenesis^40^. In the same way, intestinal cell kinase (serine/threonine protein kinase) plays an important role in the proliferation and differentiation of intestinal epithelial cells^41^. Another pathway pointed out is JAK-STAT signalling pathway, which regulates the transcription of numerous genes and has been implicated in the pathogenesis of several diseases, including inflammatory bowel disease^42^. In addition, it has also been linked to the response of intestinal enterocytes to a high-fat diet, which triggers a transient activation of intestinal stem cells^43^.

Historically, in vivo experiments have largely been performed on males to avoid complications related to feminine hormonal cycles, estrous cycle stages, etc., which has led to a sex-biased imbalance in research^44^. Genetic and transcriptional mechanisms, and regulatory pathways underlying sexual differentiation result in developmental and progression differences in various pathologies, as well as in response to treatment. There are also major sex differences in lipid and lipoprotein metabolism^45–47^. Interestingly, it has been shown that the estrogen receptor modulates the processing of certain miRNAs^48^. Other studies show that the expression profile of miRNAs in adipose tissue is influenced by gonadal hormones and that they also change in response to HFD. Thus, these different expression profiles may contribute to variations in gene expression in adipose tissue due to sex, as well as in the development of adipose tissue and diet-induced obesity^49^. Here, variability was lessened when gender was taken into consideration in the analysis of postprandial intestinal miRNA expression levels in the kinetic study, reinforcing the hypothesis that there are sex-based differences in the modulation of miRNA levels in response to the ingestion dietary lipids in these mice.

*Dicer1* disrupted mice have induced lipid accumulation in the small intestine^50^ and a severely impaired ability to process dietary triglycerides^15^. Here, *Dicer1* KO mice showed a decreased expression of markers related to the maintenance of the identity and maturation state of the intestine, such as Lgr5, Ki67, lysozyme C1 and mucin 2^21^. This could help explain the disorganization and alteration of the intestinal barrier described by McKenna et al., which would contribute to the decreased weight in KO mice compared to their litter controls. Nevertheless, in the study by McKenna et al. the smaller size KO animals compared to their litter controls became indistinguishable at 7 weeks of age, whereas the difference in weight seen here was still significant in adult animals. This discrepancy could be due to the gender-separated analysis performed in this study. Indeed, statistical significance was lost when the analysis was performed without gender separation (p value = 0.0921). Regarding total plasma lipids, there were no significant differences between WT and KO mice at baseline, which is in agreement the study by McKenna et al.^15^. However, plasma triglyceride levels were lower in KO mice after the oral challenge, independently of gender, which seems to be in line with the lack of proper dietary TG processing in *Dicer1* deficient mice reported by McKenna et al. However, FPLC results indicate a slight difference in lipidic distribution in lipoproteins, with decreased cholesterol and TG levels in HDL and in VLDL, respectively, both at baseline and postprandially. This contradiction between the total levels and the profile of individual lipoproteins, would indicate an underlying mechanism, probably related to the lack of miRNAs, which affects the distribution of lipids between the different lipoproteins and postprandially. Whether or not this disparity is influenced by the action of intestinal miRNAs is unknown and deserves further attention. Gut lipid data also appear to support that proper TG processing is lacking in mutants and the smaller increase in TG levels (compared to WT) seen 2 h after the lipid challenge may be due to an absorption problem. Since TG levels normalize with respect to WT animals after 4 h, this could indicate there is a delay in dietary lipid absorption. This possible delay was also observed for cholesterol, although statistical significance was found only in jejunum and ileum. The possible delay in lipid absorption is likely to be in agreement with lipid accumulation observed in the feces of mice lacking intestinal *Dicer1*, on high-fat chow, compared to controls^15^. Confirmation that these changes in intestinal lipid absorption are mediated by miRNAs and characterization of the mechanisms of action involved could be important in the search for new therapeutic targets against lipid-related disorders.

Finally, two different in vitro models representing the intestinal epithelium were used here to study miRNAs expression in response to lipid challenges. In vitro models are employed to facilitate the study of complex in vivo phenomena in a simplified context, allowing well-controlled and repeatable conditions for the evaluation of cell response. Caco-2 cells are extensively used models of the intestinal epithelium. When differentiated, these cells adopt a behaviour similar to enterocytes^51^. The expression profile of microRNAs in differentiated Caco-2 cells, which is shown here for the first time, indicates their similarity to the profile of intestinal miRNAs^15^, which is one indicator that this in vitro model could be appropriate to study miRNAs in an intestinal epithelium context. Nevertheless, apart from other limitations, this model is far from recapitulating the complex microenvironment of the intestine^52^. Here, for example, other characteristic miRNAs were not expressed in Caco-2 cells, such as the let-7 family, and the response to postprandial lipids only partially mimicked the one observed in vivo. In this sense, intestinal organoids (enteroids), which are propagated from epithelial intestinal stem cells that exist within the adult intestinal tissue, better recapitulate the diversity, development, and differentiation of the intestinal epithelium^53^. Here, characteristic miRNAs present in the small intestine^15^, including the let-7 family, were identified in enteroids. However, this model assumes a static, closed-light model, with inaccessibility to the apical surface and lacking the relationship with other tissues, immune cells, vasculature, and mechanical forces that recreate and define normal intestinal physiology^52^, which could help explain why miRNA expression did not consistently mirror in vivo results. Ideally, future research could be carried out in improved culture systems such as a Transwell permeable insert allowing access to the apical and basolateral surfaces of intestinal epithelial cells^54^. Another recently developed method manages to reverse the epithelial polarity of enteroids, maintaining the ability to differentiate between the various lineages of intestinal cells and to absorb nutrients adequately^55^.

To conclude, here, we aimed at finding robust miRNA candidates, whose expression changes in response to lipid challenges would be highly reproducible across different models. However, the variability found in response to lipid challenges between the different experimental cohorts and in vitro models revealed that miRNAs do not respond exactly in the same manner in all the systems tested. One explanation for this is that the initial screening was performed in the whole small intestine of male mice, while most subsequent assessments were performed in both genders, in separate intestine sections, and/or in different models. Thus, the significant changes in expression observed initially, which determined the selection of candidate miRNAs for subsequent validations, may have been attenuated. Another possible reason for the moderate consistency found between models is that the changes in the levels of some miRNAs seen in the screening might be due to other intestinal biological processes not specifically related to the response to dietary lipids.

Overall, several miRNAs (miR-218-2-3p, -138-1-3p, -147-3p, -217-5p, -216b-5p, 544-3p, 202-5p, -129-2-3p, -208b-3p, -30d-5p, -15a-5p and -218-5p) were modulated in response to dietary lipids, hence are likely to participate in the regulation of lipid metabolism and call for further research. miR-129-2-3p, -138–1-3p, -147-3p and -218-2-3p expression responses were the most consistent among all the different experimental models studied here.

## Methods

### Murine models and crosses

All procedures involving mice were carried out in accordance with guidelines of the European Communities Directive 86/609/EEC, and experimental protocols performed on animals were approved by the Animal Ethics Committee (Proex 281/15 and Proex 282/15) of the Ramón y Cajal Hospital (Madrid, Spain). Mice were housed in a standard animal facility and maintained under controlled conditions, in temperature- (25 ± 2 °C) and lighting-controlled rooms (12 h light–dark cycles), with food and water available ad libitum.

Male C57BL/6 mice were purchased from Charles River (Écully, France). To generate intestinal-specific *Dicer1* knockout (KO) mice and wild-type (WT) littermates, *Dicer1*^floxed^ (B6.Cg-*Dicer1* < tm1Bdh > /J) and Vil-cre (B6.Cg-Tg(*Vil1*-cre)997 Gum/J) mice were purchased from Jackson Laboratories. Females homozygous for the *Dicer1*^flox^ were cross-bred with Vil-cre transgenic males to generate *Dicer*^flox/+^; Vil-cre mice. Mice were mated with each other to generate homozygous mutants, *Dicer*^flox/flox^; Vil-cre, which were used for the experiments aiming to compare WT (refering to *Dicer1*^loxP/loxP^; Vil-cre(−) mice) and KO (refering to *Dicer1*^loxP/loxP^; Vil-cre(+), intestinal-specific *Dicer1* knockout mice) littermates.

### In vivo experiments

C57BL/6 male mice were used in the screening for intestinal miRNAs responsive to acute and chronic lipid challenges. The acute experimental setting consisted in the administration, by oral gavage, of either a cholesterol-enriched olive oil solution (250 µL of olive oil with 40 mg of cholesterol) or 300 µL of water (control); mice were sacrificed 2 h after the oral challenge (n = 5 per group). As for the chronic experimental settings mice were fed either a commercial atherogenic diet (TD 02028, Harlan Laboratories) or a standard diet (A04, SAFE, controls); mice were sacrificed after 4 days (n = 5 per group) in one setting and after 20 weeks (n = 4 per group) in the other.

C57BL/6 male mice used in the kinetic were divided into five experimental groups and sacrificed after 0 (basal time point), 30, 60, 120 and 240 min of receiving an oral administration of a cholesterol-enriched olive oil solution.

The first cohort of intestinal *Dicer1* knockout mice and their WT littermates were used to assess sex influence in intestinal miRNAs expression in response to dietary lipids. Mice were divided in four experimental groups: (1) WT, control (*n* = 12 males; *n* = 10 females); (2) WT, treated (*n* = 11 males; *n* = 10 females); (3) KO, control (*n* = 12 males; *n* = 11 females) and (4) KO, treated (*n* = 14 males; *n* = 11 females). The second cohort of intestinal *Dicer1* knockout mice and their WT littermates was used to assess miRNA expression in the different small intestine sections in response to an oral lipid challenge. Mice were divided in eight experimental groups: (1) Females, control, 2 h (*n* = 6 KO; *n* = 5 WT); (2) Females, control, 4 h (*n* = 4 KO; *n* = 5 WT); (3) Females, treated, 2 h (*n* = 5 KO; *n* = 9 WT); 4) Females, treated, 4 h (*n* = 4 KO; *n* = 9 WT); (5) Males, control, 2 h (*n* = 7 KO; *n* = 4 WT); (6) Males, control, 4 h (*n* = 5 KO; *n* = 4 WT); (7) Males, treated, 2 h (*n* = 9 KO; *n* = 8 WT) and (8) Males, treated, 4 h (*n* = 5 KO; *n* = 8 WT).

#### Sample collection

Mice were anesthetized with a mixture of ketamine/xylazine, sacrificed by exsanguination and perfused with PBS. To obtain plasma, blood samples were collected in Na_2_-EDTA tubes and centrifuged at 1500×*g* for 15 min, at 4 °C, and stored at − 80 °C. All gastrointestinal tissues collected (small and large intestine, and liver) were immediately frozen with liquid nitrogen and stored at − 80 °C until processing.

### In vitro models

#### Caco-2 cells

Caco-2 cells, obtained from the American Type Culture Collection (ATCC, Ref HTB37), were seeded and maintained in DMEM (Dulbecco’s Modified Eagle’s Medium) containing 10% FBS, 1% l-Glutamine and antibiotics (Pen/Strep, Amphotericin) (Lonza) at 37 °C and 5% CO_2_. For differentiated cells, Caco-2 cells between passes 28–55, were split and seeded at a density of 1.2 × 10^5^ cells/well on 12 mm diameter polycarbonate Transwell filter inserts (Corning) with 0.4 µm pore size. The inserts were placed onto 12-well plates and monolayers were cultured until differentiation for 3 weeks.

#### Mouse intestinal organoids

For the obtention of mouse intestinal organoids (enteroids) derived from adult intestinal stem cells (ISC), intestinal crypts were isolated from the small intestine of male and female WT mice, following the protocol provided by STEMCELL Technologies. Crypts were cultured at 37 °C until organoids were fully developed (5–7 days).

#### Lipid micelles used to mirror a dietary lipid challenge

Postprandial micelles (PPM) were prepared following a previously described method^56^. The final composition of the micelles obtained was 0.6 mmol/L of oleic acid (OA, Sigma O1383), 0.2 mmol/L of l‐α‐phosphatidylcholine (LPC, Sigma L4129), 0.05 mmol/L of cholesterol (CHOL, Sigma C3045), 0.2 mmol/L of 2-monooleylglycerol (2-MO, Sigma M7765) and 2 mM of taurocholate (TC, Sigma T9034). Empty micelles (EM), without oleic acid and cholesterol, were generated to serve as a vehicle control. In the case of Caco-2 cells, micelles were prepared in DMEM with 10% LPDS, while for enteroids DMEM/F12 was used. Control groups were incubated with DMEM with 10% LPDS in the case of Caco-2 cells or DMEM/F12 for enteroids. For both intestinal epithelium models incubation periods consisted of 24 h.

### Analysis of microRNAs

#### RNA isolation

Frozen tissues were homogenized in QIAzol Lysis Reagent (Qiagen), using *TIO basic ULTRA-TURRAX* (IKA). Total RNA was isolated using miRNeasy mini kits (Qiagen) following the manufacturer´s instructions. Total RNA was quantified in a Nanodrop-2000 spectrophotometer (ThermoScientific) and RNA integrity was evaluated with the Agilent 2100 Bioanalyzer system.

#### Small RNA-Seq

Total RNA with a RIN score > 8.0 was used for cDNA library preparation. Library preparations were performed using a NEBNext Small RNA Library Prep Set for Illumina (NEB), following the manufacturer’s protocol. cDNA library was sequenced with NextSeq. 500 from Illumina. FASTQC tool was used to evaluate quality control. Bowtie2 was used for sequence alignment with reference genome. RNA counting was carried out using HTSeq-count to allow differential expression analysis. Small RNA sequencing was performed by *Sistemas Genómicos* (Valencia, Spain).

#### RT-qPCR

Total RNA was reverse-transcribed (RT) to cDNA using miScript II RT Kit (Qiagen), according to the manufacturer’s instructions. miRNAs expression levels were determined, in duplicates, by quantitative real time PCR (qPCR) on a 7900HT Real-Time PCR system (Applied Biosystems), using miScript SYBR Green PCR Kits (Qiagen).

The screening for intestinal miRNAs regulated by dietary lipid challenges was performed using miRNome panels (641 mature miRNAs) Version 3 (Exiqon). Relative expression was calculated using GenEx software (https://multid.se/genex/, MultiD Analyses) for data pre-processing and analysis. The common miRNAs among the different experimental studies were represented in a Venn diagram^57^.

Relative quantification of selected miRNAs was performed using specific primer for each miRNA (Isogen). Mature miRNA primer sequences were obtained from the miRBase (www.mirbase.org). Relative expression levels were calculated with the 2^−ΔΔCt^ method^58^ using RNU1A1, RNU6 and RNU43 to normalization.

### Lipid analysis

#### Plasma lipid and lipoprotein profiles

Total plasma triglycerides and cholesterol concentrations were analyzed with a microtiter assay using Triglycerides-LQ GPO-POD and Cholesterol-LQ CHOD-POD (Spinreact) commercial kits. For plasma lipoprotein profiles, 400 µL of pooled plasmas from each group were analysed using fast phase liquid chromatography (FPLC) method. Separation was performed by gel filtration using a Superose 6 h 10/30 column (Pharmacia) as previously described^59^.

#### Intestinal and hepatic lipid levels

Lipid extraction for the analysis of triglycerides, cholesterol and phospholipids in tissues was carried out according to Folch’s method^60^, followed by solubilization in water by the addition of Triton X-100^61^. Lipid analysis was performed using a standard enzymatic-colorimetric determination (Cholesterol CHOD-POD, Triglycerides GPO-POD and Phospholipids CHO-POD, Spinreact).

### Statistical analysis

Results were expressed as mean ± standard error of the mean and statistically significant differences between groups were assessed using student’s T-test, one-way ANOVA or two-way ANOVA depending on the experimental setting. p < 0.05 was considered as statistically significant. All statistical analyses were performed using GraphPad Prism software (V.7, GraphPad Software Inc, San Diego, https://www.graphpad.com/scientific-software/prism/), except for the screening assay (GenEx software).

### Bioinformatic approach

To predict miRNA targets, the list of 12 selected miRNAs was imported into the miRWalk database^62,63^. Then, the validated interactions with an entry in miRTarBase (results from 3UTR and binding probability > 0.95) were selected for a total of 180 different genes. Next, a geneset enrichment analysis (GSEA) was performed using the Kyoto Encyclopedia of Genes and Genomes (KEEG) pathways and Gene Ontology (GO) enrichment analysis (Biological process). The relation between miRNAs with their target genes (Genes ID) and pathways (KEGG and GO annotations) were summarized and represented using the Circos visualization tool^64^.

## Supplementary information

Supplementary Information.

## References

[1] Abumrad NA, Davidson NO (2012). Role of the gut in lipid homeostasis. Physiol. Rev..

[2] Dash S, Xiao C, Morgantini C, Lewis GF (2015). New insights into the regulation of chylomicron production. Annu. Rev. Nutr..

[3] Xiao C, Hsieh J, Adeli K, Lewis GF (2011). Gut-liver interaction in triglyceride-rich lipoprotein metabolism. Am. J. Physiol. Endocrinol. Metab..

[4] Tonkin A, Byrnes A (2014). Treatment of dyslipidemia. F1000Prime Rep..

[5] Helwak A, Kudla G, Dudnakova T, Tollervey D (2013). Mapping the human miRNA interactome by CLASH reveals frequent noncanonical binding. Cell.

[6] Tüfekci KU, Öner MG, Meuwissen RLJ, Genç Ş (2014). The role of microRNAs in human diseases. Methods Mol. Biol..

[7] Asadzadeh Z (2019). microRNAs in cancer stem cells: Biology, pathways, and therapeutic opportunities. J. Cell. Physiol..

[8] Nuñez-Sánchez MA (2015). MicroRNAs expression in normal and malignant colon tissues as biomarkers of colorectal cancer and in response to pomegranate extracts consumption: Critical issues to discern between modulatory effects and potential artefacts. Mol. Nutr. Food Res..

[9] Chendrimada TP (2005). TRBP recruits the Dicer complex to Ago2 for microRNA processing and gene silencing. Nature.

[10] Schober A, Weber C (2016). Mechanisms of microRNAs in atherosclerosis. Annu. Rev. Pathol. Mech. Dis..

[11] Zhou Z, Schober A, Nazari-Jahantigh M (2018). Dicer promotes endothelial recovery and limits lesion formation after vascular injury through miR-126-5p. Int. J. Cardiol..

[12] Wei Y (2018). Dicer in macrophages prevents atherosclerosis by promoting mitochondrial oxidative metabolism. Circulation.

[13] Li T (2018). The deletion of dicer in mature myelinating glial cells causes progressive axonal degeneration but not overt demyelination in adult mice. Glia.

[14] Mang GM (2015). A neuron-specific deletion of the microRNA-processing enzyme dicer induces severe but transient obesity in mice. PLoS ONE.

[15] McKenna LB (2010). MicroRNAs control intestinal epithelial differentiation, architecture, and barrier function. Gastroenterology.

[16] Liu S (2016). The host shapes the gut microbiota via fecal microRNA. Cell Host Microbe.

[17] Nakato G (2016). Epithelium-intrinsic microRNAs contribute to mucosal immune homeostasis by promoting M-cell maturation. PLoS ONE.

[18] Sun LN (2017). Dicer suppresses cytoskeleton remodeling and tumorigenesis of colorectal epithelium by miR-324-5p mediated suppression of HMGXB3 and WASF-2. Oncotarget.

[19] Briand O (2016). Liver X receptor regulates triglyceride absorption through intestinal down-regulation of scavenger receptor class B, type 1. Gastroenterology.

[20] Mantilla-Escalante DC (2019). Postprandial circulating miRNAs in response to a dietary fat challenge. Nutrients.

[21] Ruiz-Roso MB (2020). Intestinal lipid metabolism genes regulated by miRNAs. Front. Genet..

[22] Small EM, Olson EN (2011). Pervasive roles of microRNAs in cardiovascular biology. Nature.

[23] Runtsch MC, Round JL, O’Connell RM (2014). MicroRNAs and the regulation of intestinal homeostasis. Front. Genet..

[24] Dalmasso G (2011). Microbiota modulate host gene expression via micrornas. PLoS ONE.

[25] Goodrich JK (2014). Human genetics shape the gut microbiome. Cell.

[26] Sukhotnik I (2004). Effect of dietary fat on early morphological intestinal adaptation in a rat with short bowel syndrome. Pediatr. Surg. Int..

[27] Clara R (2017). Metabolic adaptation of the small intestine to short- and medium-term high-fat diet exposure. J. Cell. Physiol..

[28] de Wit NJ (2008). The role of the small intestine in the development of dietary fat-induced obesity and insulin resistance in C57BL/6J mice. BMC Med. Genomics.

[29] de Wit NJW (2011). Dose-dependent effects of dietary fat on development of obesity in relation to intestinal differential gene expression in C57BL/6J mice. PLoS ONE.

[30] Bresciani E (2019). miRNA-218 targets lipin-1 and glucose transporter type 4 genes in 3T3-L1 cells treated with lopinavir/ritonavir. Front. Pharmacol..

[31] Zhang P (2019). Lipin 2/3 phosphatidic acid phosphatases maintain phospholipid homeostasis to regulate chylomicron synthesis. J. Clin. Investig..

[32] Eskildsen T (2011). MicroRNA-138 regulates osteogenic differentiation of human stromal (mesenchymal) stem cells in vivo. Proc. Natl. Acad. Sci. U.S.A..

[33] Ye D (2012). MiR-138 promotes induced pluripotent stem cell generation through the regulation of the P53 signaling. Stem Cells.

[34] Huang S (2019). miR-129-2-3p directly targets SYK gene and associates with the risk of ischaemic stroke in a Chinese population. J. Cell. Mol. Med..

[35] Umehara T (2019). Identification of specific miRNAs in neutrophils of type 2 diabetic mice: Overexpression of miRNA-129-2-3p accelerates diabetic wound healing. Diabetes.

[36] Spinosa M (2018). Human mesenchymal stromal cell-derived extracellular vesicles attenuate aortic aneurysm formation and macrophage activation via microRNA-147. FASEB J..

[37] Chatterjee V (2014). MicroRNA-147b regulates vascular endothelial barrier function by targeting ADAM15 expression. PLoS ONE.

[38] Sui C-J (2016). MicroRNA-147 suppresses human hepatocellular carcinoma proliferation migration and chemosensitivity by inhibiting HOXC6. Am. J. Cancer Res..

[39] Shen J, Niu W, Zhang H, Jun M, Zhang H (2018). Downregulation of microRNA-147 inhibits cell proliferation and increases the chemosensitivity of gastric cancer cells to 5-fluorouracil by directly targeting PTEN. Oncol. Res..

[40] Gregorieff A (2005). Expression pattern of Wnt signaling components in the adult intestine. Gastroenterology.

[41] Fu Z, Kim J, Vidrich A, Sturgill TW, Cohn SM (2009). Intestinal cell kinase, a MAP kinase-related kinase, regulates proliferation and G1 cell cycle progression of intestinal epithelial cells. Am. J. Physiol. Gastrointest. Liver Physiol..

[42] Coskun M, Salem M, Pedersen J, Nielsen OH (2013). Involvement of JAK/STAT signaling in the pathogenesis of inflammatory bowel disease. Pharmacol. Res..

[43] von Frieling J (2020). A high-fat diet induces a microbiota-dependent increase in stem cell activity in the Drosophila intestine. PLoS Genet..

[44] Beery AK, Zucker I (2011). Sex bias in neuroscience and biomedical research. Neurosci. Biobehav. Rev..

[45] Palmisano BT, Zhu L, Eckel RH, Stafford JM (2018). Sex differences in lipid and lipoprotein metabolism. Mol. Metab..

[46] Mischke M (2013). Maternal Western-style high fat diet induces sex-specific physiological and molecular changes in two-week-old mouse offspring. PLoS ONE.

[47] Steegenga WT (2014). Sexually dimorphic characteristics of the small intestine and colon of prepubescent C57BL/6 mice. Biol. Sex Differ..

[48] Castellano L (2009). The estrogen receptor-α-induced microRNA signature regulates itself and its transcriptional response. Proc. Natl. Acad. Sci. U.S.A..

[49] Link JC, Reue K (2017). Genetic basis for sex differences in obesity and lipid metabolism. Annu. Rev. Nutr..

[50] Huang TC (2012). Regulation of lipid metabolism by dicer revealed through SILAC mice. J. Proteome Res..

[51] Antunes F, Andrade F, Araújo F, Ferreira D, Sarmento B (2013). Establishment of a triple co-culture in vitro cell models to study intestinal absorption of peptide drugs. Eur. J. Pharm. Biopharm..

[52] Costa J, Ahluwalia A (2019). Advances and current challenges in intestinal in vitro model engineering: A digest. Front. Bioeng. Biotechnol..

[53] Sato T (2009). Single Lgr5 stem cells build crypt-villus structures in vitro without a mesenchymal niche. Nature.

[54] Altay G (2019). Self-organized intestinal epithelial monolayers in crypt and villus-like domains show effective barrier function. Sci. Rep..

[55] Co JY (2019). Controlling epithelial polarity: A human enteroid model for host-pathogen interactions. Cell Rep..

[56] Chateau D (2005). Lipid micelles stimulate the secretion of triglyceride-enriched apolipoprotein B48-containing lipoproteins by Caco-2 cells. J. Cell. Physiol..

[57] Oliveros, J. C. *VENNY. An Interactive Tool for Comparing Lists with Venn Diagrams*. (2007). https://bioinfogp.cnb.csic.es/tools/venny/index.html, 10.1017/S0266267108002022.

[58] Livak KJ, Schmittgen TD (2001). Analysis of relative gene expression data using real-time quantitative PCR and the 2-ΔΔCT method. Methods.

[59] Tomé-Carneiro J (2016). Hydroxytyrosol supplementation modulates the expression of miRNAs in rodents and in humans. J. Nutr. Biochem..

[60] Folch J, Lees M, Stanley GHS (1957). A simple method for the isolation and purification of total lipides from animal tissues. J. Biol. Chem..

[61] Carr TP, Andresen CJ, Rudel LL (1993). Enzymatic determination of triglyceride, free cholesterol, and total cholesterol in tissue lipid extracts. Clin. Biochem..

[62] Dweep H, Sticht C, Pandey P, Gretz N (2011). MiRWalk—Database: Prediction of possible miRNA binding sites by ‘walking’ the genes of three genomes. J. Biomed. Inform..

[63] Dweep H, Gretz N (2015). MiRWalk20: A comprehensive atlas of microRNA-target interactions. Nat. Methods.

[64] Krzywinski M (2009). Circos: An information aesthetic for comparative genomics. Genome Res..

