# Supplementary material for: Intestinal miRNAs regulated in response to dietary lipids
Source: Sci Rep. 2020 Nov 3;10:18921. doi: 10.1038/s41598-020-75751-w (PMC7642330; doi:10.1038/s41598-020-75751-w)
Supplement: Supplementary file 1 — Supplementary Information. [file 41598_2020_75751_MOESM1_ESM.pdf]

Intestinal miRNAs regulated in response to dietary lipids

Judit Gil-Zamorano[1], João Tomé-Carneiro[2], María-Carmen López de las Hazas[1], Lorena del Pozo-Acebo[1], M. Carmen Crespo[2], Diego Gómez-Coronado[3,4], Luis A. Chapado[1], Emilio Herrera[5]; María-Jesús Latasa[1], María Belén Ruiz-Roso[1], Mónica Castro-Camarero[6], Olivier Briand [7], Alberto Dávalos[1]*.

[1] Laboratory of Epigenetics of Lipid Metabolism, Madrid Institute for Advanced Studies Food (IMDEA Food), CEI UAM + CSIC, 28049 Madrid, Spain.

[2] Laboratory of Functional Foods, Madrid Institute for Advanced Studies Food (IMDEA Food), CEI UAM CSIC, 28049 Madrid, Spain.

[3]Servicio de Bioquímica-Investigación, Hospital Universitario Ramón y Cajal, IRYCIS, 28034 Madrid, Spain

[4]Centre of Biomedical Research in Physiopathology of Obesity and Nutrition (CIBEROBN), Instituto de Salud Carlos III, 28029 Madrid, Spain.

[5]Depatment of Biochemistry and Chemistry, Faculties of Pharmacy and Medicine, Universidad San Pablo CEU, 28668 Madrid, Spain.

[6]Servicio de Cirugía Experimental, Hospital Universitario Ramón y Cajal, IRYCIS, 28034 Madrid, Spain

[7]Univ. Lille, Inserm, CHU Lille, Institut Pasteur de Lille, U1011- EGID, F-59000 Lille, France.

* Correspondence to: Dr. Alberto Dávalos, Laboratory of Epigenetics of Lipid Metabolism. IMDEA Food Institute. Carretera de Canto Blanco, 8. 28049 Madrid (Spain).

E-mail: alberto.davalos@imdea.org

Tel. +34912796985

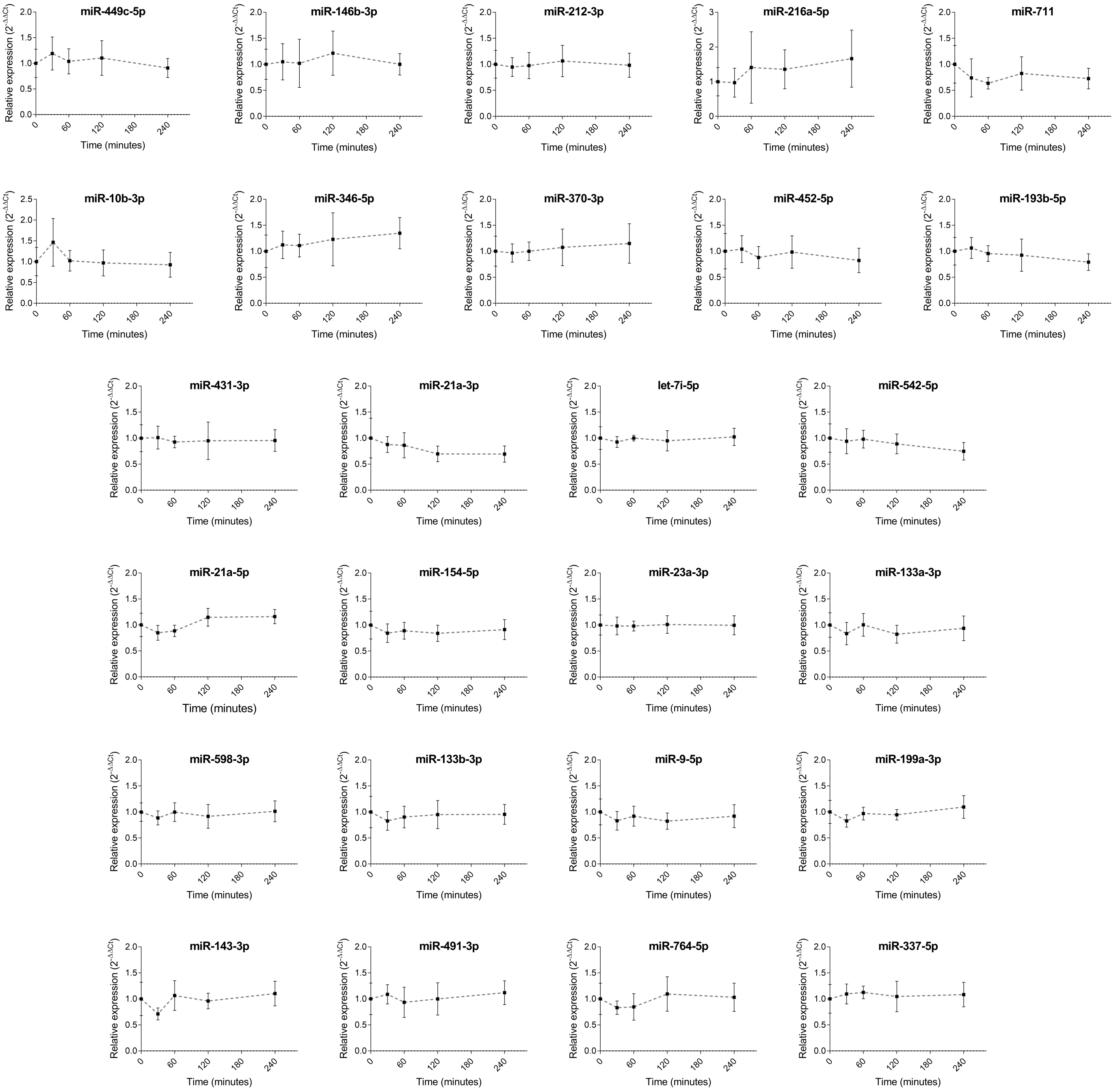

Supplementary Figure 1. Time-course (0, 60, 120 and 240 minutes) expression of miRNAs showing non-significant differences (i.e. 26 out of the 35 previously selected, please see figure 1E) in response to an oral lipid challenge (250 μL olive oil enriched with 40 mg of cholesterol) administrated to male C57BL/6 mice; *n* ≥ 7. Data are shown as mean ± SD.

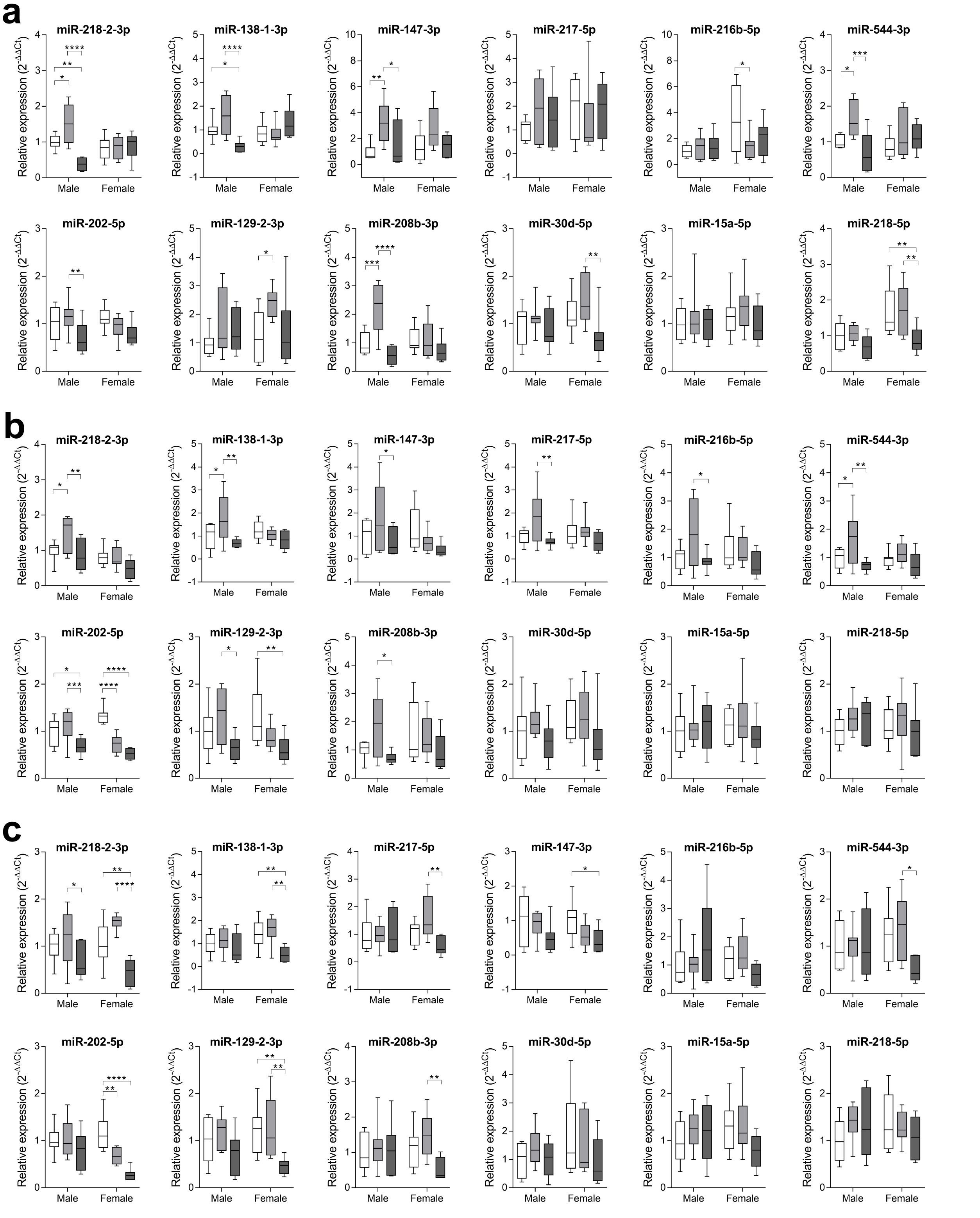

Supplementary Figure 2. Relative expression levels of selected miRNAs in different sections of the small intestine, duodenum (**a**), jejunum (**b**) and ileum (**c**), in response to an oral lipid challenge (250 µL olive oil enriched with 40 mg of cholesterol) in male and female WT mice. Control (water, white bars), 2 hours after lipid challenge (grey bars) and 4 hours after lipid challenge (dark grey bars). Data are shown as mean ± SD; $n \geq 8$. *$p < 0.05$, **$p < 0.001$, ***$p < 0.0001$, compared with male control. Two-way ANOVA was followed by Bonferroni's post-hoc tests for multiple comparisons. *$p < 0.05$, **$p < 0.01$, ***$p < 0.001$, ****$p < 0.0001$.

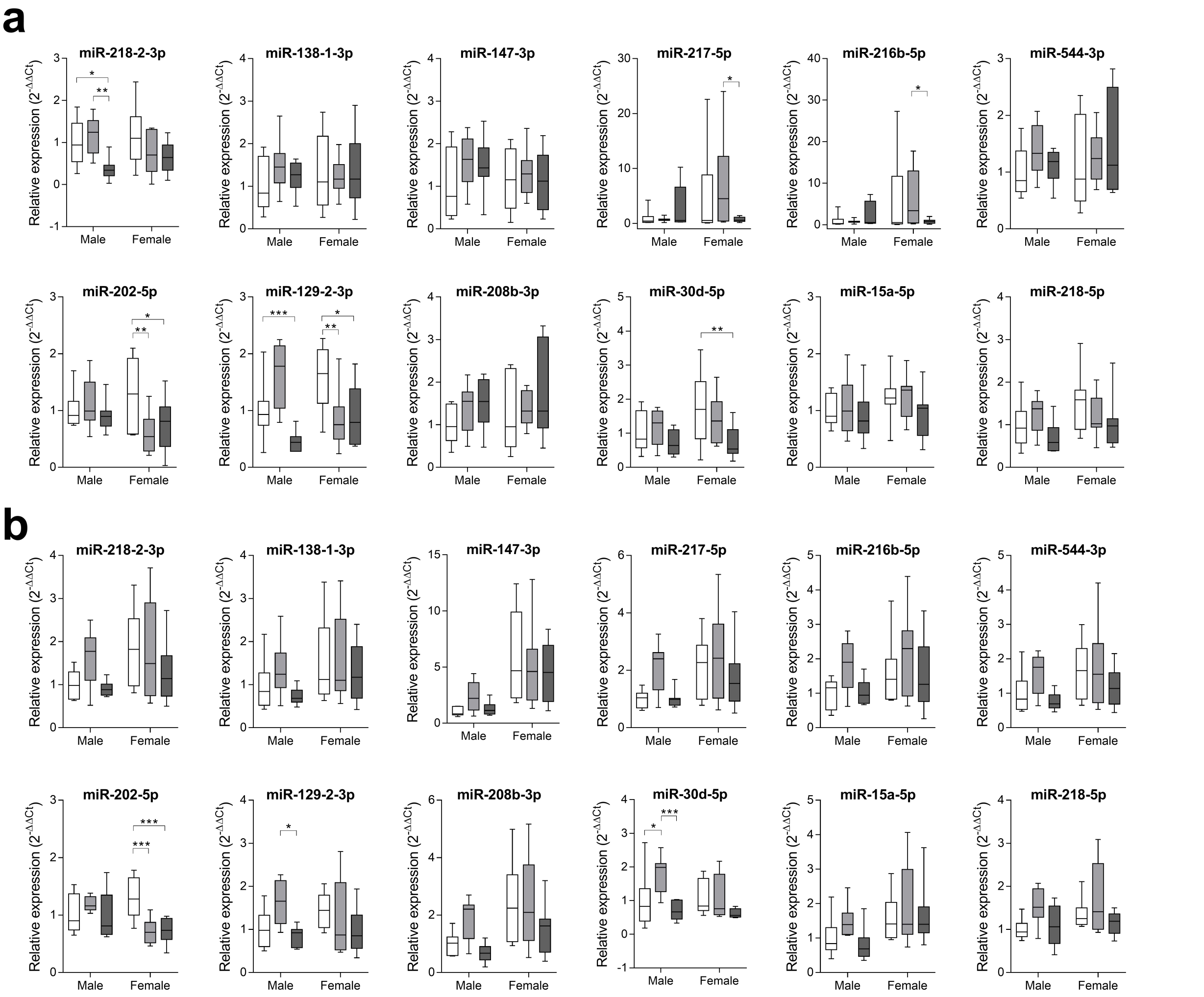

Supplementary Figure 3. Relative expression levels of selected miRNAs in colon (**a**) and liver (**b**) in response to an oral lipid challenge (250 µL olive oil enriched with 40 mg of cholesterol) in male and female WT mice. Controls (water, white bars), 2 hours after lipid challenge (grey bars) and 4 hours after lipid challenge (dark grey bars). Data are shown as mean ± SD; *n* ≥ 8. Two-way ANOVA was followed by Bonferroni's post-hoc tests for multiple comparisons. *$p < 0.05$, **$p < 0.01$, ***$p < 0.001$, ****$p < 0.0001$.

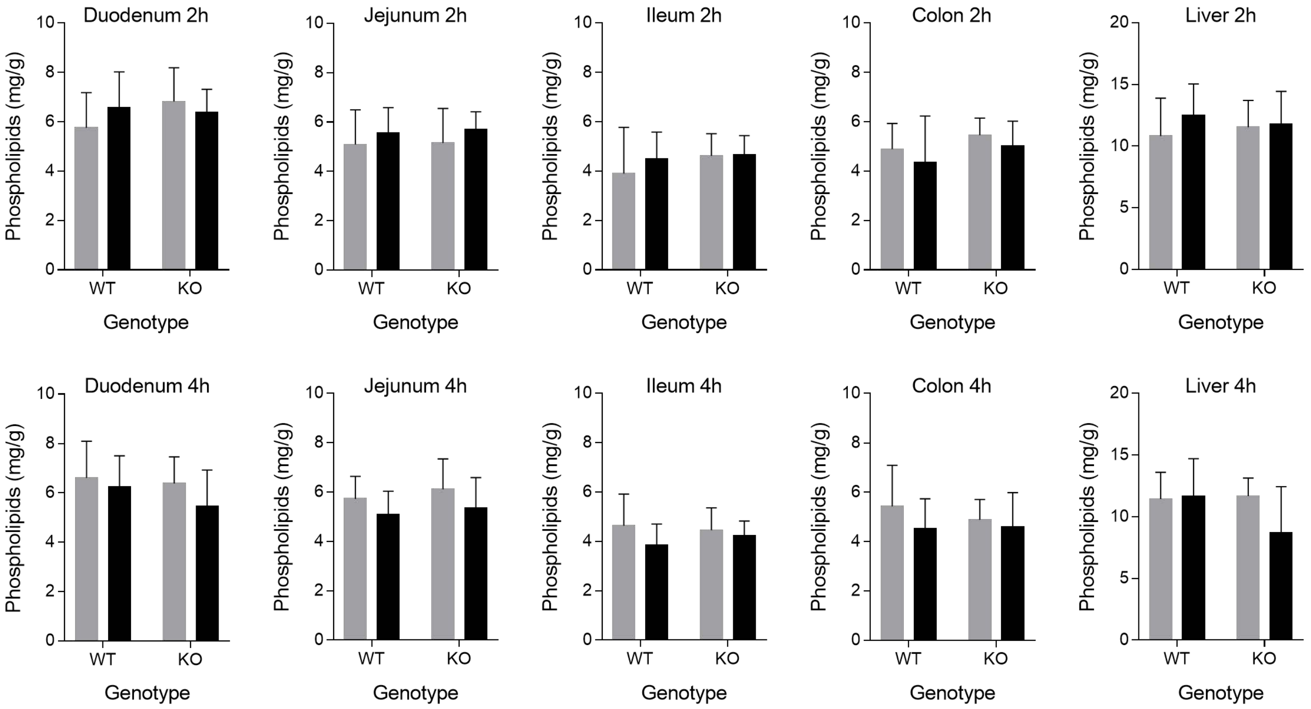

Supplementary Figure 4. Intestinal and hepatic phospholipids (mg/g) in WT (*Dicer1*$^{loxP/loxP}$, Vil-cre(-)) an KO (*Dicer1*$^{loxP/loxP}$, Vil-cre(+)) mice, 2 and 4 hours after an oral administration of cholesterol-enriched olive oil (black) or water (controls; grey); $n \geq 9$. Data are represented as mean ± SD. Two-way ANOVA was followed by Bonferroni's post-hoc tests for multiple comparisons. *p < 0.05, **p < 0.01, ***p < 0.001, ****p < 0.0001.

Supplementary Table 1. Intestinal miRNAs altered in *accute* study. Found 2 h after an oral administration of cholesterol-enriched (40 mg) olive oil (250 µL) in male C57BL/6 mice. [a] In bold, microRNAs with significant change, unpaired T-test, $p < 0.05$. [b] Pulse oleic *vs.* control. [c] Benjamini and Hochberg corrected values.

miRNA[a]	Fold Change[b]	P-value	P (Benjamini-Hochberg)[c]
**mmu-miR-1198-3p**	**-6.31**	**6.01E-07**	**3.80E-04**
**mmu-miR-146b-3p**	**5.43**	**9.17E-06**	**2.17E-03**
**mmu-miR-344d-3p**	**6.43**	**1.04E-05**	**2.17E-03**
**mmu-miR-295-3p**	**-4.30**	**3.65E-05**	**5.72E-03**
**mmu-miR-1953**	**-7.70**	**6.05E-05**	**7.44E-03**
**mmu-miR-202-5p**	**3.66**	**7.12E-05**	**7.44E-03**
**mmu-miR-218-2-3p**	**-12.77**	**8.54E-05**	**7.65E-03**
**mmu-miR-669k-3p**	**3.09**	**4.15E-04**	**2.97E-02**
**mmu-miR-193b-5p**	**6.01**	**4.26E-04**	**2.97E-02**
**mmu-miR-344c-3p**	**-2.63**	**5.56E-04**	**3.49E-02**
**mmu-miR-742-5p**	**-6.80**	**9.35E-04**	**5.33E-02**
**mmu-miR-669i**	**-2.62**	**3.29E-03**	**1.52E-01**
**mmu-miR-21a-3p**	**1.87**	**4.24E-03**	**1.66E-01**
**mmu-miR-431-3p**	**-4.19**	**6.66E-03**	**2.46E-01**
**mmu-miR-669h-3p**	**-2.18**	**8.38E-03**	**2.77E-01**
**mmu-miR-1938**	**3.92**	**1.14E-02**	**3.40E-01**
**mmu-miR-337-5p**	**2.78**	**1.29E-02**	**3.65E-01**
**mmu-miR-711**	**3.46**	**1.34E-02**	**3.65E-01**
**mmu-miR-208a-5p**	**1.71**	**1.62E-02**	**4.01E-01**
**mmu-miR-495-3p**	**1.39**	**1.64E-02**	**4.01E-01**
**mmu-miR-300-5p**	**2.33**	**1.66E-02**	**4.01E-01**
**mmu-miR-291a-5p**	**2.30**	**1.77E-02**	**4.05E-01**
**mmu-miR-216a-5p**	**-10.27**	**1.83E-02**	**4.05E-01**
**mmu-miR-217-5p**	**-11.16**	**1.87E-02**	**4.05E-01**
**mmu-miR-216b-5p**	**-11.10**	**2.54E-02**	**5.18E-01**
**mmu-miR-133a-5p**	**-2.54**	**2.71E-02**	**5.32E-01**
**mmu-miR-1929-5p**	**-2.89**	**2.86E-02**	**5.44E-01**
**mmu-miR-212-3p**	**1.45**	**3.02E-02**	**5.56E-01**
**mmu-miR-449c-5p**	**1.55**	**3.51E-02**	**6.05E-01**
**mmu-miR-879-3p**	**-3.42**	**3.53E-02**	**6.05E-01**
**mmu-miR-343**	**2.48**	**3.57E-02**	**6.05E-01**
**mmu-miR-1894-5p**	**-2.24**	**3.93E-02**	**6.15E-01**
**mmu-miR-433-5p**	**-1.71**	**4.13E-02**	**6.15E-01**
**mmu-miR-671-3p**	**2.77**	**4.15E-02**	**6.15E-01**
**mmu-miR-412-3p**	**1.85**	**4.19E-02**	**6.15E-01**
**mmu-miR-129-2-3p**	**-3.38**	**4.38E-02**	**6.24E-01**
**mmu-miR-770-5p**	**3.07**	**4.53E-02**	**6.31E-01**
**mmu-miR-466h-5p**	**-2.53**	**4.95E-02**	**6.75E-01**
mmu-miR-672-5p	1.67	5.32E-02	6.93E-01
mmu-miR-1193-5p	2.16	5.50E-02	6.93E-01
mmu-miR-1951	1.71	5.55E-02	6.93E-01
mmu-miR-383-5p	-2.57	5.56E-02	6.93E-01
mmu-miR-1895	1.61	5.63E-02	6.93E-01
mmu-miR-762	1.68	5.78E-02	6.97E-01
mmu-miR-344b-3p	-3.23	6.10E-02	7.15E-01
mmu-miR-34c-3p	-3.18	6.29E-02	7.15E-01
mmu-miR-362-3p	-1.34	6.39E-02	7.15E-01
mmu-miR-323-3p	3.02	6.65E-02	7.15E-01
mmu-miR-450a-2-3p	2.14	6.67E-02	7.15E-01
mmu-miR-296-3p	-1.54	6.73E-02	7.15E-01
mmu-miR-183-3p	1.36	7.22E-02	7.43E-01
mmu-miR-742-3p	2.53	7.23E-02	7.43E-01
mmu-miR-1981-5p	-1.86	7.60E-02	7.48E-01
mmu-miR-92b-3p	2.13	7.88E-02	7.48E-01
mmu-miR-1947-5p	3.71	7.93E-02	7.48E-01
mmu-miR-448-3p	-1.64	8.01E-02	7.48E-01
mmu-miR-1247-5p	-2.01	8.12E-02	7.48E-01
mmu-miR-294-5p	2.10	8.27E-02	7.48E-01
mmu-miR-698-3p	-1.53	8.39E-02	7.48E-01
mmu-miR-452-5p	-1.93	8.47E-02	7.48E-01
mmu-miR-487b-3p	1.35	9.07E-02	7.83E-01
mmu-miR-615-3p	1.73	9.33E-02	7.83E-01
mmu-miR-298-5p	1.71	9.42E-02	7.83E-01
mmu-miR-761	-1.74	9.47E-02	7.83E-01
mmu-miR-1955-5p	-2.38	9.50E-02	7.83E-01
mmu-miR-615-5p	1.85	9.76E-02	7.86E-01
mmu-miR-1966-5p	3.00	1.06E-01	8.21E-01
mmu-miR-1948-3p	-2.63	1.06E-01	8.21E-01
mmu-miR-32-5p	-1.29	1.07E-01	8.21E-01
mmu-miR-18b-5p	-1.85	1.10E-01	8.28E-01
mmu-miR-465b-5p	2.29	1.14E-01	8.52E-01
mmu-miR-29c-5p	-1.38	1.16E-01	8.52E-01
mmu-miR-384-3p	-2.44	1.18E-01	8.52E-01
mmu-miR-122-5p	-1.65	1.19E-01	8.52E-01
mmu-let-7a-2-3p	-3.10	1.20E-01	8.52E-01
mmu-miR-1967	-1.80	1.23E-01	8.52E-01
mmu-miR-203-5p	-1.32	1.25E-01	8.52E-01
mmu-miR-1902	1.96	1.25E-01	8.52E-01
mmu-miR-501-5p	-1.51	1.26E-01	8.52E-01
mmu-miR-1927	-2.11	1.26E-01	8.52E-01
mmu-miR-542-3p	-1.64	1.32E-01	8.78E-01
mmu-miR-297a-3p	2.10	1.37E-01	8.96E-01
mmu-miR-10a-3p	-1.57	1.41E-01	9.00E-01

mmu-miR-504-5p	2.09	1.46E-01	9.27E-01
mmu-miR-23a-5p	-2.22	1.49E-01	9.37E-01
mmu-miR-744-5p	1.28	1.52E-01	9.43E-01
mmu-miR-376a-3p	-1.50	1.70E-01	9.73E-01
mmu-miR-673-5p	-3.19	1.74E-01	9.73E-01
mmu-miR-1186a	-3.25	1.74E-01	9.73E-01
mmu-miR-804	-2.21	1.76E-01	9.73E-01
mmu-miR-409-3p	1.65	1.77E-01	9.73E-01
mmu-miR-101a-5p	-1.46	1.79E-01	9.73E-01
mmu-miR-700-3p	1.93	1.79E-01	9.73E-01
mmu-miR-483-3p	1.61	1.79E-01	9.73E-01
mmu-miR-692	-1.65	1.80E-01	9.73E-01
mmu-miR-34c-5p	1.20	1.85E-01	9.73E-01
mmu-miR-369-3p	1.29	1.87E-01	9.73E-01
mmu-miR-1907	1.57	1.96E-01	9.73E-01
mmu-miR-17-5p	1.23	1.98E-01	9.73E-01
mmu-miR-532-3p	-1.39	2.01E-01	9.73E-01
mmu-miR-874-3p	1.37	2.01E-01	9.73E-01
mmu-miR-500-3p	-1.30	2.05E-01	9.73E-01
mmu-miR-687	-1.33	2.07E-01	9.73E-01
mmu-miR-669d-5p	-1.55	2.08E-01	9.73E-01
mmu-miR-433-3p	2.08	2.10E-01	9.73E-01
mmu-miR-484	1.20	2.11E-01	9.73E-01
mmu-miR-329-3p	-1.46	2.11E-01	9.73E-01
mmu-miR-7b-5p	1.19	2.12E-01	9.73E-01
mmu-miR-670-5p	-1.75	2.12E-01	9.73E-01
mmu-miR-654-3p	1.51	2.13E-01	9.73E-01
mmu-miR-873a-5p	-3.16	2.15E-01	9.73E-01
mmu-miR-1195	1.38	2.16E-01	9.73E-01
mmu-miR-574-3p	1.41	2.16E-01	9.73E-01
mmu-miR-30d-3p	-1.32	2.26E-01	9.87E-01
mmu-miR-139-3p	2.00	2.27E-01	9.87E-01
mmu-miR-2137	1.40	2.28E-01	9.87E-01
mmu-miR-1946b	-1.64	2.30E-01	9.87E-01
mmu-miR-669n	-1.60	2.32E-01	9.87E-01
mmu-miR-669j	-1.38	2.34E-01	9.87E-01
mmu-miR-434-5p	1.24	2.38E-01	9.87E-01
mmu-miR-147-3p	1.28	2.48E-01	9.87E-01
mmu-miR-875-5p	1.63	2.48E-01	9.87E-01
mmu-miR-335-3p	-1.19	2.49E-01	9.87E-01
mmu-miR-496a-3p	-1.59	2.49E-01	9.87E-01
mmu-miR-466j	-2.07	2.49E-01	9.87E-01
mmu-miR-466k	-1.71	2.53E-01	9.87E-01
mmu-miR-431-5p	1.24	2.55E-01	9.87E-01
mmu-miR-466b-5p	-2.09	2.55E-01	9.87E-01
mmu-miR-384-5p	1.40	2.55E-01	9.87E-01

mmu-miR-872-3p	-1.20	2.57E-01	9.87E-01
mmu-miR-193a-5p	-1.71	2.59E-01	9.87E-01
mmu-miR-1982.1-3p	-7.95	2.66E-01	9.87E-01
mmu-miR-369-5p	1.64	2.67E-01	9.87E-01
mmu-miR-211-5p	-1.73	2.70E-01	9.87E-01
mmu-miR-291b-5p	1.47	2.72E-01	9.87E-01
mmu-miR-210-3p	1.59	2.74E-01	9.87E-01
mmu-miR-490-5p	-1.26	2.74E-01	9.87E-01
mmu-miR-539-5p	-2.08	2.75E-01	9.87E-01
mmu-miR-27a-3p	1.14	2.77E-01	9.87E-01
mmu-miR-1905	1.24	2.78E-01	9.87E-01
mmu-miR-137-3p	1.11	2.80E-01	9.87E-01
mmu-miR-743a-3p	-1.50	2.83E-01	9.87E-01
mmu-miR-130a-3p	1.16	2.83E-01	9.87E-01
mmu-miR-26a-5p	1.24	2.85E-01	9.87E-01
mmu-miR-1940	1.47	2.85E-01	9.87E-01
mmu-miR-106a-5p	1.20	2.91E-01	9.87E-01
mmu-miR-1196-5p	1.68	2.93E-01	9.87E-01
mmu-miR-30a-3p	-1.16	2.96E-01	9.87E-01
mmu-miR-129-1-3p	-2.31	2.97E-01	9.87E-01
mmu-miR-681	-1.46	2.98E-01	9.87E-01
mmu-miR-467f	1.50	3.02E-01	9.87E-01
mmu-miR-320-3p	1.25	3.11E-01	9.87E-01
mmu-miR-708-3p	1.67	3.17E-01	9.87E-01
mmu-miR-704	1.93	3.23E-01	9.87E-01
mmu-miR-1b-5p	1.89	3.27E-01	9.87E-01
mmu-miR-208b-3p	-1.53	3.27E-01	9.87E-01
mmu-miR-191-3p	-1.28	3.28E-01	9.87E-01
mmu-miR-676-3p	-1.25	3.33E-01	9.87E-01
mmu-miR-802-5p	-1.17	3.34E-01	9.87E-01
mmu-miR-467e-5p	-1.19	3.38E-01	9.87E-01
mmu-miR-449a-5p	1.35	3.39E-01	9.87E-01
mmu-miR-598-3p	-1.43	3.41E-01	9.87E-01
mmu-miR-1191	-1.35	3.46E-01	9.87E-01
mmu-miR-410-3p	1.27	3.60E-01	9.87E-01
mmu-miR-30d-5p	-1.16	3.60E-01	9.87E-01
mmu-miR-501-3p	-1.20	3.64E-01	9.87E-01
mmu-miR-224-5p	1.34	3.68E-01	9.87E-01
mmu-miR-466d-5p	-2.25	3.72E-01	9.87E-01
mmu-miR-28a-3p	-1.28	3.77E-01	9.87E-01
mmu-miR-551b-3p	1.57	3.79E-01	9.87E-01
mmu-miR-132-3p	1.20	3.87E-01	9.87E-01
mmu-miR-872-5p	-1.22	3.87E-01	9.87E-01
mmu-miR-544-3p	-1.64	3.88E-01	9.87E-01
mmu-miR-677-5p	-1.38	3.88E-01	9.87E-01
mmu-miR-374b-5p	1.14	3.88E-01	9.87E-01

mmu-miR-340-5p	-1.22	3.88E-01	9.87E-01
mmu-miR-30e-3p	-1.19	3.89E-01	9.87E-01
mmu-miR-30c-1-3p	-1.32	3.92E-01	9.87E-01
mmu-miR-26b-5p	1.11	3.96E-01	9.87E-01
mmu-miR-409-5p	1.34	3.96E-01	9.87E-01
mmu-miR-466d-3p	1.72	3.97E-01	9.87E-01
mmu-miR-135b-5p	1.34	3.97E-01	9.87E-01
mmu-miR-297c-5p	-1.49	3.99E-01	9.87E-01
mmu-miR-29a-5p	-1.18	4.03E-01	9.87E-01
mmu-miR-1935	1.73	4.03E-01	9.87E-01
mmu-let-7b-5p	1.18	4.04E-01	9.87E-01
mmu-miR-10b-5p	1.13	4.10E-01	9.87E-01
mmu-miR-325-5p	-1.55	4.11E-01	9.87E-01
mmu-miR-1198-5p	-1.26	4.12E-01	9.87E-01
mmu-miR-337-3p	-1.34	4.25E-01	9.87E-01
mmu-miR-743b-5p	1.40	4.25E-01	9.87E-01
mmu-miR-450b-5p	-1.77	4.31E-01	9.87E-01
mmu-miR-331-5p	-1.65	4.32E-01	9.87E-01
mmu-miR-546	-1.41	4.33E-01	9.87E-01
mmu-miR-148a-3p	-1.24	4.33E-01	9.87E-01
mmu-miR-153-3p	-1.20	4.35E-01	9.87E-01
mmu-miR-713	1.15	4.35E-01	9.87E-01
mmu-miR-196a-5p	1.81	4.37E-01	9.87E-01
mmu-miR-668-3p	-1.27	4.38E-01	9.87E-01
mmu-miR-2183	-1.30	4.38E-01	9.87E-01
mmu-miR-346-5p	-1.63	4.38E-01	9.87E-01
mmu-miR-1930-5p	-1.35	4.41E-01	9.87E-01
mmu-miR-15b-3p	-1.20	4.44E-01	9.87E-01
mmu-miR-503-3p	1.15	4.46E-01	9.87E-01
mmu-let-7e-3p	-1.29	4.47E-01	9.87E-01
mmu-miR-196b-3p	-1.32	4.48E-01	9.87E-01
mmu-miR-297b-5p	1.46	4.51E-01	9.87E-01
mmu-miR-1957a	1.32	4.52E-01	9.87E-01
mmu-miR-138-1-3p	-1.47	4.52E-01	9.87E-01
mmu-miR-99b-3p	-1.35	4.54E-01	9.87E-01
mmu-miR-758-3p	1.41	4.56E-01	9.87E-01
mmu-miR-328-3p	1.33	4.57E-01	9.87E-01
mmu-let-7c-5p	1.12	4.62E-01	9.87E-01
mmu-let-7b-3p	1.33	4.63E-01	9.87E-01
mmu-miR-376b-5p	-1.27	4.66E-01	9.87E-01
mmu-miR-376c-5p	1.50	4.68E-01	9.87E-01
mmu-miR-130b-3p	1.09	4.71E-01	9.87E-01
mmu-miR-10a-5p	1.11	4.72E-01	9.87E-01
mmu-miR-330-3p	-1.36	4.75E-01	9.87E-01
mmu-miR-465a-5p	1.22	4.77E-01	9.87E-01
mmu-miR-694	1.30	4.80E-01	9.87E-01
mmu-miR-18a-3p	1.15	4.80E-01	9.87E-01
mmu-miR-361-5p	1.57	4.81E-01	9.87E-01
mmu-miR-411-3p	1.26	4.81E-01	9.87E-01
mmu-miR-33-3p	-1.23	4.82E-01	9.87E-01
mmu-miR-106b-3p	-1.23	4.83E-01	9.87E-01
mmu-miR-362-5p	-1.15	4.86E-01	9.87E-01
mmu-miR-425-3p	-1.21	4.87E-01	9.87E-01
mmu-miR-1a-3p	1.12	4.90E-01	9.87E-01
mmu-miR-195a-5p	1.13	4.91E-01	9.87E-01
mmu-miR-93-3p	-1.21	4.92E-01	9.87E-01
mmu-miR-98-5p	1.12	4.93E-01	9.87E-01
mmu-miR-540-3p	-1.27	4.93E-01	9.87E-01
mmu-miR-342-5p	-1.22	4.95E-01	9.87E-01
mmu-miR-34b-5p	-1.11	4.96E-01	9.87E-01
mmu-miR-201-5p	1.66	4.97E-01	9.87E-01
mmu-miR-103-2-5p	-1.18	4.98E-01	9.87E-01
mmu-miR-301a-3p	1.16	5.00E-01	9.87E-01
mmu-miR-188-5p	-1.14	5.05E-01	9.87E-01
mmu-miR-425-5p	1.11	5.05E-01	9.87E-01
mmu-miR-466f-5p	-1.38	5.06E-01	9.87E-01
mmu-miR-1900	-1.29	5.08E-01	9.87E-01
mmu-miR-154-3p	1.54	5.08E-01	9.87E-01
mmu-miR-1903	-1.28	5.10E-01	9.87E-01
mmu-miR-1897-5p	-1.36	5.13E-01	9.87E-01
mmu-miR-511-5p	-1.57	5.13E-01	9.87E-01
mmu-miR-24-3p	1.13	5.14E-01	9.87E-01
mmu-miR-146a-5p	1.11	5.14E-01	9.87E-01
mmu-let-7i-5p	1.14	5.14E-01	9.87E-01
mmu-miR-212-5p	-1.25	5.15E-01	9.87E-01
mmu-miR-494-3p	1.20	5.15E-01	9.87E-01
mmu-miR-468-3p	1.39	5.15E-01	9.87E-01
mmu-miR-1839-5p	-1.14	5.17E-01	9.87E-01
mmu-miR-16-1-3p	-1.25	5.18E-01	9.87E-01
mmu-miR-467c-5p	1.19	5.21E-01	9.87E-01
mmu-miR-34b-3p	1.25	5.23E-01	9.87E-01
mmu-miR-200b-3p	1.12	5.27E-01	9.87E-01
mmu-miR-377-3p	1.11	5.31E-01	9.87E-01
mmu-miR-206-3p	-1.63	5.34E-01	9.87E-01
mmu-miR-15b-5p	1.09	5.38E-01	9.87E-01
mmu-miR-144-3p	-1.18	5.39E-01	9.87E-01
mmu-let-7d-5p	1.13	5.39E-01	9.87E-01
mmu-let-7a-5p	1.11	5.42E-01	9.87E-01
mmu-miR-29b-3p	-1.11	5.44E-01	9.87E-01
mmu-miR-151-5p	1.11	5.45E-01	9.87E-01
mmu-let-7d-3p	1.40	5.47E-01	9.87E-01
mmu-miR-26a-1-3p	-1.24	5.48E-01	9.87E-01

mmu-miR-467h	-1.27	5.48E-01	9.87E-01
mmu-miR-26b-3p	-1.13	5.48E-01	9.87E-01
mmu-miR-467b-3p	-1.51	5.48E-01	9.87E-01
mmu-miR-126-5p	-1.14	5.48E-01	9.87E-01
mmu-miR-1964-3p	-1.48	5.49E-01	9.87E-01
mmu-miR-125b-2-3p	-1.24	5.50E-01	9.87E-01
mmu-miR-27a-5p	-1.27	5.50E-01	9.87E-01
mmu-miR-1932	-1.21	5.51E-01	9.87E-01
mmu-let-7f-1-3p	-1.18	5.52E-01	9.87E-01
mmu-miR-1952	1.36	5.53E-01	9.87E-01
mmu-miR-674-3p	-1.16	5.55E-01	9.87E-01
mmu-miR-192-3p	-1.15	5.55E-01	9.87E-01
mmu-miR-221-3p	1.10	5.55E-01	9.87E-01
mmu-miR-465c-5p	-1.33	5.55E-01	9.87E-01
mmu-miR-148b-3p	1.09	5.62E-01	9.87E-01
mmu-miR-297a-5p	-1.19	5.63E-01	9.87E-01
mmu-miR-466f	1.36	5.64E-01	9.87E-01
mmu-let-7g-3p	-1.13	5.65E-01	9.87E-01
mmu-miR-1941-3p	-1.12	5.66E-01	9.87E-01
mmu-miR-669e-5p	-1.46	5.66E-01	9.87E-01
mmu-miR-127-5p	-1.17	5.66E-01	9.87E-01
mmu-miR-871-5p	1.11	5.68E-01	9.87E-01
mmu-miR-327	-1.16	5.68E-01	9.87E-01
mmu-miR-709	1.15	5.69E-01	9.87E-01
mmu-miR-21a-5p	1.10	5.70E-01	9.87E-01
mmu-miR-125a-3p	1.38	5.70E-01	9.87E-01
mmu-miR-22-3p	1.09	5.72E-01	9.87E-01
mmu-miR-27b-5p	-1.25	5.75E-01	9.87E-01
mmu-let-7c-1-3p	1.29	5.78E-01	9.87E-01
mmu-miR-1894-3p	-1.37	5.81E-01	9.87E-01
mmu-miR-125b-1-3p	1.44	5.82E-01	9.87E-01
mmu-miR-214-5p	-1.13	5.85E-01	9.87E-01
mmu-miR-199a-3p	1.11	5.87E-01	9.87E-01
mmu-miR-199b-5p	-1.13	5.88E-01	9.87E-01
mmu-miR-25-3p	1.09	5.89E-01	9.87E-01
mmu-miR-532-5p	-1.14	5.92E-01	9.87E-01
mmu-let-7i-3p	-1.17	5.93E-01	9.87E-01
mmu-miR-196b-5p	-1.36	5.94E-01	9.87E-01
mmu-miR-136-5p	-1.12	5.99E-01	9.87E-01
mmu-miR-351-5p	1.17	6.00E-01	9.87E-01
mmu-miR-423-5p	1.13	6.03E-01	9.87E-01
mmu-miR-145a-3p	-1.13	6.04E-01	9.87E-01
mmu-miR-378a-5p	-1.14	6.04E-01	9.87E-01
mmu-miR-202-3p	1.52	6.08E-01	9.87E-01
mmu-miR-667-3p	-1.33	6.08E-01	9.87E-01
mmu-let-7f-2-3p	-1.14	6.10E-01	9.87E-01

mmu-miR-181d-5p	1.10	6.14E-01	9.87E-01
mmu-miR-1949	-1.14	6.15E-01	9.87E-01
mmu-miR-434-3p	-1.13	6.17E-01	9.87E-01
mmu-miR-126-3p	1.09	6.18E-01	9.87E-01
mmu-miR-18a-5p	1.09	6.20E-01	9.87E-01
mmu-miR-340-3p	-1.14	6.22E-01	9.87E-01
mmu-miR-142-3p	-1.09	6.22E-01	9.87E-01
mmu-miR-455-5p	1.10	6.25E-01	9.87E-01
mmu-miR-134-5p	-1.29	6.30E-01	9.87E-01
mmu-miR-33-5p	-1.09	6.32E-01	9.87E-01
mmu-miR-423-3p	1.10	6.34E-01	9.87E-01
mmu-miR-130b-5p	-1.15	6.34E-01	9.87E-01
mmu-miR-652-3p	1.11	6.35E-01	9.87E-01
mmu-miR-708-5p	-1.09	6.36E-01	9.87E-01
mmu-miR-710	1.19	6.41E-01	9.87E-01
mmu-miR-669l-5p	-1.23	6.42E-01	9.87E-01
mmu-miR-335-5p	1.12	6.43E-01	9.87E-01
mmu-miR-187-3p	-1.15	6.45E-01	9.87E-01
mmu-miR-186-5p	1.07	6.50E-01	9.87E-01
mmu-miR-339-3p	-1.15	6.52E-01	9.87E-01
mmu-miR-466f-3p	-1.18	6.53E-01	9.87E-01
mmu-miR-15a-3p	-1.14	6.57E-01	9.87E-01
mmu-miR-129-5p	1.24	6.60E-01	9.87E-01
mmu-miR-674-5p	-1.09	6.61E-01	9.87E-01
mmu-miR-764-5p	1.18	6.63E-01	9.87E-01
mmu-miR-146b-5p	1.06	6.65E-01	9.87E-01
mmu-miR-1249-3p	-1.13	6.66E-01	9.87E-01
mmu-miR-744-3p	-1.12	6.67E-01	9.87E-01
mmu-miR-429-3p	1.07	6.68E-01	9.87E-01
mmu-miR-99b-5p	-1.09	6.68E-01	9.87E-01
mmu-miR-10b-3p	-1.38	6.72E-01	9.87E-01
mmu-miR-1934-5p	-1.33	6.72E-01	9.87E-01
mmu-miR-135a-5p	1.07	6.72E-01	9.87E-01
mmu-miR-205-5p	-1.15	6.74E-01	9.87E-01
mmu-miR-338-3p	-1.11	6.75E-01	9.87E-01
mmu-miR-124-3p	-1.09	6.77E-01	9.87E-01
mmu-miR-455-3p	1.08	6.77E-01	9.87E-01
mmu-miR-706	1.12	6.79E-01	9.87E-01
mmu-miR-194-2-3p	-1.13	6.82E-01	9.87E-01
mmu-miR-141-5p	-1.11	6.82E-01	9.87E-01
mmu-miR-541-5p	1.13	6.82E-01	9.87E-01
mmu-miR-194-5p	-1.06	6.83E-01	9.87E-01
mmu-miR-1956	1.31	6.86E-01	9.87E-01
mmu-miR-182-5p	1.09	6.87E-01	9.87E-01
mmu-miR-293-3p	1.23	6.88E-01	9.87E-01
mmu-miR-31-5p	-1.08	6.88E-01	9.87E-01

mmu-miR-190a-5p	-1.08	6.89E-01	9.87E-01
mmu-miR-223-3p	1.07	6.92E-01	9.87E-01
mmu-miR-96-5p	-1.08	6.94E-01	9.87E-01
mmu-miR-325-3p	-1.25	6.98E-01	9.87E-01
mmu-miR-467b-5p	-1.07	6.98E-01	9.87E-01
mmu-miR-543-3p	-1.13	7.01E-01	9.87E-01
mmu-miR-324-3p	1.07	7.01E-01	9.87E-01
mmu-miR-138-5p	1.03	7.05E-01	9.87E-01
mmu-miR-450a-5p	1.10	7.06E-01	9.87E-01
mmu-miR-379-3p	1.08	7.07E-01	9.87E-01
mmu-miR-155-5p	1.10	7.07E-01	9.87E-01
mmu-miR-497-5p	1.06	7.09E-01	9.87E-01
mmu-miR-183-5p	1.06	7.10E-01	9.87E-01
mmu-miR-490-3p	-1.10	7.11E-01	9.87E-01
mmu-miR-150-5p	-1.11	7.11E-01	9.87E-01
mmu-miR-214-3p	-1.08	7.15E-01	9.87E-01
mmu-miR-486-3p	1.24	7.18E-01	9.87E-01
mmu-miR-20b-5p	1.09	7.20E-01	9.87E-01
mmu-miR-219-1-3p	-1.25	7.24E-01	9.87E-01
mmu-miR-148a-5p	-1.14	7.26E-01	9.87E-01
mmu-miR-7a-1-3p	-1.08	7.27E-01	9.87E-01
mmu-miR-105	1.25	7.30E-01	9.87E-01
mmu-miR-382-5p	1.07	7.33E-01	9.87E-01
mmu-miR-16-5p	1.06	7.34E-01	9.87E-01
mmu-miR-345-3p	1.11	7.34E-01	9.87E-01
mmu-miR-322-3p	-1.09	7.35E-01	9.87E-01
mmu-miR-185-5p	1.06	7.37E-01	9.87E-01
mmu-miR-181a-1-3p	-1.12	7.37E-01	9.87E-01
mmu-miR-222-3p	-1.08	7.38E-01	9.87E-01
mmu-miR-1946a	-1.11	7.39E-01	9.87E-01
mmu-miR-380-3p	-1.14	7.41E-01	9.87E-01
mmu-miR-143-3p	1.06	7.42E-01	9.87E-01
mmu-miR-200c-3p	1.06	7.43E-01	9.87E-01
mmu-miR-324-5p	1.07	7.47E-01	9.87E-01
mmu-miR-19b-1-5p	1.09	7.53E-01	9.87E-01
mmu-let-7e-5p	1.06	7.54E-01	9.87E-01
mmu-miR-154-5p	1.07	7.57E-01	9.87E-01
mmu-miR-19a-3p	1.05	7.58E-01	9.87E-01
mmu-miR-467d-5p	1.08	7.58E-01	9.87E-01
mmu-miR-142-5p	-1.06	7.65E-01	9.87E-01
mmu-miR-200c-5p	1.08	7.66E-01	9.87E-01
mmu-miR-23b-3p	1.05	7.73E-01	9.87E-01
mmu-miR-1928	1.15	7.75E-01	9.87E-01
mmu-miR-379-5p	1.08	7.77E-01	9.87E-01
mmu-miR-195a-3p	-1.11	7.78E-01	9.87E-01
mmu-miR-669b-5p	-1.12	7.78E-01	9.87E-01

mmu-miR-363-3p	1.07	7.81E-01	9.87E-01
mmu-miR-1961	1.05	7.86E-01	9.87E-01
mmu-miR-376c-3p	-1.05	7.86E-01	9.87E-01
mmu-miR-30c-2-3p	1.08	7.88E-01	9.87E-01
mmu-miR-200b-5p	-1.07	7.88E-01	9.87E-01
mmu-miR-151-3p	1.08	7.90E-01	9.87E-01
mmu-miR-467a-5p	-1.05	7.90E-01	9.87E-01
mmu-miR-666-3p	-1.27	7.92E-01	9.87E-01
mmu-miR-1306-3p	-1.28	7.93E-01	9.87E-01
mmu-miR-7a-5p	1.04	7.97E-01	9.87E-01
mmu-miR-34a-5p	-1.05	8.01E-01	9.87E-01
mmu-miR-92a-2-5p	-1.13	8.01E-01	9.87E-01
mmu-miR-467g	-1.12	8.02E-01	9.87E-01
mmu-miR-299a-3p	1.09	8.03E-01	9.87E-01
mmu-miR-186-3p	-1.08	8.06E-01	9.87E-01
mmu-miR-760-3p	1.16	8.07E-01	9.87E-01
mmu-miR-381-3p	-1.08	8.08E-01	9.87E-01
mmu-miR-326-3p	1.05	8.09E-01	9.87E-01
mmu-miR-188-3p	-1.12	8.09E-01	9.87E-01
mmu-miR-712-5p	-1.06	8.11E-01	9.87E-01
mmu-miR-152-3p	-1.05	8.13E-01	9.87E-01
mmu-miR-207	-1.11	8.13E-01	9.87E-01
mmu-let-7f-5p	1.04	8.14E-01	9.87E-01
mmu-miR-30b-5p	1.03	8.19E-01	9.87E-01
mmu-let-7g-5p	-1.05	8.19E-01	9.87E-01
mmu-miR-1983	-1.07	8.20E-01	9.87E-01
mmu-miR-20b-3p	1.11	8.20E-01	9.87E-01
mmu-miR-540-5p	1.06	8.21E-01	9.87E-01
mmu-miR-99a-5p	-1.05	8.21E-01	9.87E-01
mmu-miR-100-5p	-1.04	8.21E-01	9.87E-01
mmu-miR-421-3p	1.06	8.22E-01	9.87E-01
mmu-miR-877-5p	1.12	8.26E-01	9.87E-01
mmu-miR-20a-3p	-1.06	8.28E-01	9.87E-01
mmu-miR-411-5p	1.04	8.29E-01	9.87E-01
mmu-miR-139-5p	1.05	8.31E-01	9.87E-01
mmu-miR-488-3p	1.07	8.34E-01	9.87E-01
mmu-miR-491-3p	-1.06	8.34E-01	9.87E-01
mmu-miR-376b-3p	1.07	8.34E-01	9.87E-01
mmu-miR-200a-5p	-1.05	8.34E-01	9.87E-01
mmu-miR-375-3p	1.04	8.35E-01	9.87E-01
mmu-miR-9-3p	-1.07	8.39E-01	9.87E-01
mmu-miR-451a	-1.06	8.40E-01	9.87E-01
mmu-miR-1199-5p	1.05	8.40E-01	9.87E-01
mmu-miR-93-5p	1.04	8.44E-01	9.87E-01
mmu-miR-1839-3p	1.06	8.48E-01	9.87E-01
mmu-miR-149-5p	1.06	8.49E-01	9.87E-01

mmu-miR-190b-5p	1.08	8.49E-01	9.87E-01
mmu-miR-181b-5p	1.04	8.50E-01	9.87E-01
mmu-miR-2139	1.07	8.52E-01	9.87E-01
mmu-miR-467d-3p	-1.06	8.52E-01	9.87E-01
mmu-miR-22-5p	-1.04	8.53E-01	9.87E-01
mmu-miR-145a-5p	1.04	8.59E-01	9.87E-01
mmu-miR-503-5p	1.04	8.59E-01	9.87E-01
mmu-miR-219-5p	-1.05	8.60E-01	9.87E-01
mmu-miR-1943-5p	1.09	8.65E-01	9.87E-01
mmu-miR-184-3p	-1.12	8.66E-01	9.87E-01
mmu-miR-465a-3p	1.03	8.66E-01	9.87E-01
mmu-miR-29b-1-5p	-1.04	8.67E-01	9.87E-01
mmu-miR-330-5p	-1.05	8.68E-01	9.87E-01
mmu-miR-23a-3p	1.03	8.69E-01	9.87E-01
mmu-miR-345-5p	1.03	8.70E-01	9.87E-01
mmu-miR-192-5p	-1.03	8.73E-01	9.87E-01
mmu-miR-30e-5p	-1.03	8.73E-01	9.87E-01
mmu-miR-17-3p	-1.03	8.76E-01	9.87E-01
mmu-miR-30c-5p	1.03	8.77E-01	9.87E-01
mmu-miR-450b-3p	1.05	8.77E-01	9.87E-01
mmu-miR-28a-5p	1.03	8.80E-01	9.87E-01
mmu-miR-191-5p	-1.02	8.82E-01	9.87E-01
mmu-miR-127-3p	-1.04	8.84E-01	9.87E-01
mmu-miR-378a-3p	-1.02	8.86E-01	9.87E-01
mmu-miR-370-3p	-1.06	8.88E-01	9.87E-01
mmu-miR-141-3p	1.02	8.92E-01	9.87E-01
mmu-miR-592-5p	-1.05	8.92E-01	9.87E-01
mmu-miR-466c-5p	1.06	8.94E-01	9.87E-01
mmu-miR-106b-5p	1.02	8.95E-01	9.87E-01
mmu-miR-342-3p	1.02	8.98E-01	9.87E-01
mmu-miR-218-5p	1.03	9.01E-01	9.87E-01
mmu-miR-323-5p	1.03	9.01E-01	9.87E-01
mmu-miR-491-5p	1.03	9.01E-01	9.87E-01
mmu-miR-676-5p	-1.06	9.01E-01	9.87E-01
mmu-miR-30a-5p	1.02	9.05E-01	9.87E-01
mmu-miR-682	-1.06	9.09E-01	9.87E-01
mmu-miR-125a-5p	1.03	9.14E-01	9.87E-01
mmu-miR-382-3p	1.07	9.14E-01	9.87E-01
mmu-miR-133b-3p	1.02	9.15E-01	9.87E-01
mmu-miR-101b-3p	-1.01	9.17E-01	9.87E-01
mmu-miR-350-3p	-1.02	9.19E-01	9.87E-01
mmu-miR-15a-5p	1.02	9.22E-01	9.87E-01
mmu-miR-19b-3p	1.01	9.27E-01	9.87E-01
mmu-miR-181c-5p	-1.01	9.30E-01	9.87E-01
mmu-miR-27b-3p	-1.02	9.30E-01	9.87E-01
mmu-miR-193b-3p	-1.02	9.31E-01	9.87E-01

mmu-miR-203-3p	-1.01	9.31E-01	9.87E-01
mmu-miR-103-3p	-1.01	9.32E-01	9.87E-01
mmu-miR-101a-3p	-1.01	9.33E-01	9.87E-01
mmu-miR-299a-5p	-1.02	9.35E-01	9.87E-01
mmu-miR-291a-3p	1.07	9.36E-01	9.87E-01
mmu-miR-1982-5p	1.02	9.37E-01	9.87E-01
mmu-miR-331-3p	1.03	9.38E-01	9.87E-01
mmu-miR-300-3p	1.01	9.41E-01	9.87E-01
mmu-miR-3107-5p	1.02	9.41E-01	9.87E-01
mmu-miR-20a-5p	1.01	9.41E-01	9.87E-01
mmu-miR-204-5p	-1.01	9.42E-01	9.87E-01
mmu-miR-301b-3p	1.02	9.43E-01	9.87E-01
mmu-miR-29c-3p	-1.01	9.45E-01	9.87E-01
mmu-miR-125b-5p	-1.02	9.45E-01	9.87E-01
mmu-miR-193a-3p	-1.01	9.45E-01	9.87E-01
mmu-let-7a-1-3p	-1.01	9.49E-01	9.87E-01
mmu-miR-107-3p	1.01	9.53E-01	9.87E-01
mmu-miR-466g	-1.02	9.54E-01	9.87E-01
mmu-miR-215-5p	-1.01	9.55E-01	9.87E-01
mmu-miR-669c-5p	-1.02	9.56E-01	9.87E-01
mmu-miR-199a-5p	1.01	9.59E-01	9.87E-01
mmu-miR-339-5p	-1.01	9.59E-01	9.87E-01
mmu-miR-29a-3p	1.01	9.60E-01	9.87E-01
mmu-miR-140-5p	-1.01	9.60E-01	9.87E-01
mmu-miR-1968-5p	-1.02	9.60E-01	9.87E-01
mmu-miR-466a-3p	-1.03	9.60E-01	9.87E-01
mmu-miR-365-3p	-1.01	9.62E-01	9.87E-01
mmu-miR-128-3p	-1.01	9.62E-01	9.87E-01
mmu-miR-380-5p	-1.01	9.63E-01	9.87E-01
mmu-miR-133a-3p	-1.01	9.64E-01	9.87E-01
mmu-miR-140-3p	-1.01	9.65E-01	9.87E-01
mmu-miR-9-5p	-1.01	9.69E-01	9.87E-01
mmu-miR-669a-5p	1.01	9.70E-01	9.87E-01
mmu-miR-691	-1.02	9.71E-01	9.87E-01
mmu-miR-296-5p	-1.01	9.71E-01	9.87E-01
mmu-miR-322-5p	1.00	9.82E-01	> 0.99
mmu-miR-671-5p	1.00	9.86E-01	> 0.99
mmu-miR-181a-5p	-1.00	9.89E-01	> 0.99
mmu-miR-542-5p	1.00	> 0.99	> 0.99
mmu-miR-344-3p	-1.00	> 0.99	> 0.99
mmu-miR-338-5p	1.00	> 0.99	> 0.99
mmu-miR-31-3p	-1.00	> 0.99	> 0.99
mmu-miR-200a-3p	1.00	> 0.99	> 0.99

Supplementary Table 2. Intestinal miRNAs altered in 4 days study. miRNAs differentially expressed after the consumption of a high-fat diet (HFD) for 4 days, in male C57BL/6 mice. [a] In bold, microRNAs with significant change, unpaired T-test, $p < 0.05$. [b] 4 days HFD *vs.* control. [c] Benjamini and Hochberg corrected values.

miRNA[a]	Fold Change[b]	P-value	P (Benjamini-Hochberg)[c]
**mmu-miR-218-2-3p**	-11.65	6.08E-05	3.05E-02
**mmu-miR-346-5p**	-6.99	1.08E-04	3.05E-02
**mmu-miR-879-3p**	-8.12	1.58E-04	3.05E-02
**mmu-miR-10b-3p**	8.51	2.28E-04	3.29E-02
**mmu-miR-138-1-3p**	3.04	5.12E-04	4.93E-02
**mmu-miR-466b-5p**	-5.95	6.07E-04	5.01E-02
**mmu-miR-147-3p**	-3.58	7.02E-04	5.07E-02
**mmu-miR-1894-3p**	-3.84	1.70E-03	1.09E-01
**mmu-miR-544-3p**	-3.73	2.51E-03	1.32E-01
**mmu-miR-711**	4.10	3.00E-03	1.44E-01
**mmu-miR-344-3p**	3.61	3.59E-03	1.60E-01
**mmu-miR-146b-3p**	4.07	6.28E-03	2.59E-01
**mmu-miR-666-3p**	-4.37	8.25E-03	3.18E-01
**mmu-miR-673-5p**	-6.53	1.10E-02	3.74E-01
**mmu-miR-466k**	5.35	1.75E-02	5.17E-01
**mmu-miR-216b-5p**	-7.67	1.79E-02	5.17E-01
**mmu-miR-212-3p**	2.15	1.88E-02	5.17E-01
**mmu-miR-208b-3p**	-2.79	1.98E-02	5.20E-01
**mmu-miR-370-3p**	2.95	2.18E-02	5.47E-01
**mmu-miR-452-5p**	2.12	2.49E-02	6.00E-01
**mmu-miR-764-5p**	2.32	3.15E-02	6.75E-01
**mmu-miR-491-3p**	-1.93	4.25E-02	8.07E-01
**mmu-miR-216a-5p**	-5.20	4.33E-02	8.07E-01
**mmu-miR-298-5p**	1.84	4.56E-02	8.23E-01
**mmu-miR-291a-5p**	2.94	4.88E-02	8.55E-01
mmu-miR-1898	3.27	5.11E-02	8.57E-01
mmu-miR-384-3p	2.37	5.47E-02	8.57E-01
mmu-miR-1982.1-3p	-29.38	5.50E-02	8.57E-01
mmu-miR-344d-3p	2.57	5.78E-02	8.57E-01
mmu-miR-16-1-3p	-1.83	5.91E-02	8.57E-01
mmu-miR-1900	-1.89	5.93E-02	8.57E-01
mmu-miR-1983	1.96	6.25E-02	8.61E-01
mmu-miR-139-3p	2.33	6.26E-02	8.61E-01
mmu-miR-672-5p	1.94	6.53E-02	8.70E-01
mmu-miR-293-3p	-2.19	6.92E-02	8.70E-01
mmu-miR-873a-5p	-3.60	7.16E-02	8.70E-01
mmu-miR-294-5p	2.45	7.51E-02	8.70E-01
mmu-miR-713	-2.17	7.60E-02	8.70E-01
mmu-miR-1933-3p	-2.10	7.61E-02	8.70E-01
mmu-miR-1941-3p	-1.86	7.94E-02	8.70E-01
mmu-miR-669c-5p	-3.17	8.32E-02	8.70E-01
mmu-miR-686	2.44	8.32E-02	8.70E-01
mmu-miR-184-3p	-1.66	8.33E-02	8.70E-01
mmu-miR-383-5p	-2.23	8.40E-02	8.70E-01
mmu-miR-669d-5p	-2.28	8.46E-02	8.70E-01
mmu-miR-105	-2.40	8.60E-02	8.70E-01
mmu-miR-684	-3.09	8.71E-02	8.70E-01
mmu-miR-1957a	2.39	9.38E-02	8.83E-01
mmu-miR-676-3p	-1.98	9.47E-02	8.83E-01
mmu-miR-692	4.04	9.95E-02	8.85E-01
mmu-miR-466f	-3.16	1.01E-01	8.85E-01
mmu-miR-296-5p	-1.57	1.03E-01	8.85E-01
mmu-miR-331-5p	2.45	1.08E-01	9.03E-01
mmu-miR-483-3p	2.62	1.09E-01	9.03E-01
mmu-miR-669j	-2.29	1.12E-01	9.03E-01
mmu-miR-490-5p	-1.50	1.13E-01	9.03E-01
mmu-miR-1903	-2.95	1.14E-01	9.03E-01
mmu-miR-1195	1.40	1.17E-01	9.10E-01
mmu-miR-18a-5p	1.63	1.19E-01	9.20E-01
mmu-miR-23a-5p	2.35	1.25E-01	9.42E-01
mmu-miR-691	-1.76	1.26E-01	9.42E-01
mmu-miR-195a-3p	-2.19	1.27E-01	9.42E-01
mmu-miR-217-5p	-2.72	1.30E-01	9.42E-01
mmu-let-7a-2-3p	-1.86	1.33E-01	9.42E-01
mmu-miR-291a-3p	3.34	1.40E-01	9.78E-01
mmu-miR-148a-5p	-2.01	1.48E-01	> 0.99
mmu-miR-1952	1.47	1.49E-01	> 0.99
mmu-miR-33-3p	-1.69	1.52E-01	> 0.99
mmu-miR-323-5p	-2.21	1.52E-01	> 0.99
mmu-miR-465b-5p	-1.69	1.58E-01	> 0.99
mmu-miR-125b-2-3p	-3.36	1.58E-01	> 0.99
mmu-miR-200c-5p	1.58	1.58E-01	> 0.99
mmu-miR-450a-5p	-3.27	1.59E-01	> 0.99
mmu-miR-26b-3p	-1.51	1.77E-01	> 0.99
mmu-miR-30d-5p	-1.41	1.78E-01	> 0.99
mmu-miR-1955-5p	-2.94	1.82E-01	> 0.99
mmu-miR-7a-1-3p	-1.65	1.83E-01	> 0.99
mmu-miR-132-3p	1.60	1.86E-01	> 0.99
mmu-miR-205-5p	-1.99	1.88E-01	> 0.99
mmu-miR-598-3p	-2.26	1.89E-01	> 0.99
mmu-miR-667-3p	-1.45	1.91E-01	> 0.99
mmu-miR-376c-3p	-1.44	1.93E-01	> 0.99

mmu-miR-382-3p	-1.95	1.96E-01	> 0.99
mmu-miR-297b-5p	1.91	1.96E-01	> 0.99
mmu-miR-202-3p	1.52	2.02E-01	> 0.99
mmu-miR-297a-5p	-1.62	2.06E-01	> 0.99
mmu-miR-1249-3p	-1.80	2.07E-01	> 0.99
mmu-miR-503-5p	-1.37	2.07E-01	> 0.99
mmu-miR-153-3p	-1.60	2.13E-01	> 0.99
mmu-miR-330-5p	-2.37	2.15E-01	> 0.99
mmu-miR-18b-5p	2.25	2.15E-01	> 0.99
mmu-miR-1902	1.84	2.22E-01	> 0.99
mmu-miR-511-5p	-1.96	2.27E-01	> 0.99
mmu-miR-1966-5p	1.52	2.27E-01	> 0.99
mmu-miR-486-3p	-1.51	2.29E-01	> 0.99
mmu-miR-448-3p	1.59	2.32E-01	> 0.99
mmu-miR-138-5p	-1.73	2.35E-01	> 0.99
mmu-miR-214-3p	-1.41	2.36E-01	> 0.99
mmu-miR-362-5p	1.57	2.36E-01	> 0.99
mmu-miR-694	1.53	2.42E-01	> 0.99
mmu-miR-468-3p	1.74	2.46E-01	> 0.99
mmu-miR-211-5p	1.47	2.52E-01	> 0.99
mmu-miR-339-5p	-1.25	2.54E-01	> 0.99
mmu-miR-467g	-1.76	2.55E-01	> 0.99
mmu-miR-297a-3p	-1.50	2.59E-01	> 0.99
mmu-miR-503-3p	-1.66	2.63E-01	> 0.99
mmu-miR-700-3p	-1.81	2.63E-01	> 0.99
mmu-miR-1947-5p	1.97	2.71E-01	> 0.99
mmu-miR-127-5p	-1.89	2.72E-01	> 0.99
mmu-miR-181c-5p	-1.41	2.77E-01	> 0.99
mmu-miR-330-3p	-1.53	2.88E-01	> 0.99
mmu-miR-742-3p	1.69	2.88E-01	> 0.99
mmu-let-7c-1-3p	-1.32	2.99E-01	> 0.99
mmu-miR-101a-5p	-1.54	3.00E-01	> 0.99
mmu-miR-1929-5p	-1.36	3.00E-01	> 0.99
mmu-miR-193a-5p	-1.39	3.01E-01	> 0.99
mmu-miR-350-3p	-1.34	3.02E-01	> 0.99
mmu-miR-491-5p	1.60	3.04E-01	> 0.99
mmu-miR-7b-5p	-1.50	3.05E-01	> 0.99
mmu-miR-671-3p	1.45	3.06E-01	> 0.99
mmu-miR-323-3p	1.58	3.07E-01	> 0.99
mmu-miR-1948-3p	-1.91	3.10E-01	> 0.99
mmu-miR-29c-5p	-1.36	3.12E-01	> 0.99
mmu-miR-190a-5p	-1.35	3.14E-01	> 0.99
mmu-miR-542-3p	-1.52	3.20E-01	> 0.99
mmu-miR-126-5p	-1.39	3.25E-01	> 0.99
mmu-miR-92b-3p	1.50	3.31E-01	> 0.99
mmu-miR-129-5p	1.92	3.32E-01	> 0.99

mmu-miR-326-3p	-1.30	3.32E-01	> 0.99
mmu-miR-7a-5p	1.34	3.40E-01	> 0.99
mmu-miR-802-5p	-1.34	3.42E-01	> 0.99
mmu-miR-378a-5p	-1.39	3.42E-01	> 0.99
mmu-miR-34a-5p	-1.27	3.44E-01	> 0.99
mmu-miR-409-3p	1.80	3.48E-01	> 0.99
mmu-miR-706	1.37	3.50E-01	> 0.99
mmu-miR-871-5p	-1.35	3.60E-01	> 0.99
mmu-miR-301b-3p	1.55	3.64E-01	> 0.99
mmu-miR-26a-1-3p	-1.49	3.66E-01	> 0.99
mmu-miR-1956	-1.59	3.67E-01	> 0.99
mmu-miR-654-3p	-1.35	3.67E-01	> 0.99
mmu-miR-466h-5p	-1.51	3.68E-01	> 0.99
mmu-miR-188-3p	-1.73	3.69E-01	> 0.99
mmu-miR-34b-3p	1.76	3.71E-01	> 0.99
mmu-miR-34c-3p	1.68	3.73E-01	> 0.99
mmu-miR-375-3p	-1.29	3.76E-01	> 0.99
mmu-miR-29b-1-5p	-1.63	3.78E-01	> 0.99
mmu-miR-130a-3p	-1.86	3.80E-01	> 0.99
mmu-miR-467f	-1.31	3.84E-01	> 0.99
mmu-miR-194-5p	-1.29	3.84E-01	> 0.99
mmu-miR-1897-5p	1.34	3.86E-01	> 0.99
mmu-miR-494-3p	-1.33	3.87E-01	> 0.99
mmu-miR-369-3p	1.50	3.93E-01	> 0.99
mmu-miR-709	1.19	3.94E-01	> 0.99
mmu-miR-96-5p	-1.29	3.99E-01	> 0.99
mmu-miR-215-5p	-1.34	4.02E-01	> 0.99
mmu-miR-874-3p	-1.38	4.03E-01	> 0.99
mmu-miR-187-3p	-1.38	4.05E-01	> 0.99
mmu-miR-376b-5p	1.86	4.06E-01	> 0.99
mmu-miR-338-5p	-1.42	4.08E-01	> 0.99
mmu-miR-322-3p	-1.45	4.10E-01	> 0.99
mmu-miR-15a-3p	-1.43	4.12E-01	> 0.99
mmu-miR-33-5p	-1.33	4.13E-01	> 0.99
mmu-miR-369-5p	1.81	4.15E-01	> 0.99
mmu-miR-31-3p	-1.33	4.16E-01	> 0.99
mmu-miR-129-2-3p	-2.29	4.20E-01	> 0.99
mmu-miR-27b-5p	-1.32	4.23E-01	> 0.99
mmu-miR-488-3p	1.50	4.27E-01	> 0.99
mmu-miR-181a-1-3p	-1.69	4.32E-01	> 0.99
mmu-miR-455-3p	-1.40	4.37E-01	> 0.99
mmu-miR-299a-5p	-1.54	4.41E-01	> 0.99
mmu-miR-382-5p	1.28	4.43E-01	> 0.99
mmu-miR-1199-5p	-1.30	4.47E-01	> 0.99
mmu-let-7f-2-3p	-1.36	4.48E-01	> 0.99
mmu-miR-140-3p	-1.24	4.49E-01	> 0.99

mmu-miR-18a-3p	1.32	4.50E-01	> 0.99
mmu-miR-296-3p	-1.30	4.53E-01	> 0.99
mmu-miR-142-5p	-1.29	4.55E-01	> 0.99
mmu-miR-28a-3p	-1.26	4.67E-01	> 0.99
mmu-miR-712-5p	1.19	4.68E-01	> 0.99
mmu-miR-151-3p	-1.24	4.69E-01	> 0.99
mmu-miR-434-3p	-1.41	4.69E-01	> 0.99
mmu-miR-467b-5p	1.44	4.71E-01	> 0.99
mmu-miR-30b-5p	-1.23	4.75E-01	> 0.99
mmu-miR-1928	-1.28	4.75E-01	> 0.99
mmu-miR-423-5p	-1.17	4.75E-01	> 0.99
mmu-miR-127-3p	-1.31	4.77E-01	> 0.99
mmu-miR-3107-5p	-1.30	4.78E-01	> 0.99
mmu-miR-146a-5p	-1.20	4.80E-01	> 0.99
mmu-miR-29c-3p	-1.25	4.80E-01	> 0.99
mmu-miR-710	-1.53	4.81E-01	> 0.99
mmu-miR-1b-5p	-1.47	4.81E-01	> 0.99
mmu-miR-133a-3p	-1.23	4.83E-01	> 0.99
mmu-miR-214-5p	-1.28	4.89E-01	> 0.99
mmu-miR-125b-1-3p	-1.67	4.91E-01	> 0.99
mmu-miR-551b-3p	-1.31	4.91E-01	> 0.99
mmu-miR-669e-5p	-1.50	4.92E-01	> 0.99
mmu-miR-27a-5p	-1.27	4.95E-01	> 0.99
mmu-miR-1964-3p	-1.68	4.96E-01	> 0.99
mmu-miR-150-5p	-1.27	4.97E-01	> 0.99
mmu-miR-669b-5p	-1.65	4.99E-01	> 0.99
mmu-miR-151-5p	-1.23	4.99E-01	> 0.99
mmu-miR-546	1.51	5.00E-01	> 0.99
mmu-miR-196a-2-3p	1.29	5.00E-01	> 0.99
mmu-miR-30c-5p	-1.25	5.00E-01	> 0.99
mmu-miR-17-5p	1.26	5.01E-01	> 0.99
mmu-let-7g-3p	-1.30	5.01E-01	> 0.99
mmu-miR-540-3p	1.49	5.03E-01	> 0.99
mmu-miR-30e-3p	-1.22	5.05E-01	> 0.99
mmu-miR-466d-3p	1.41	5.06E-01	> 0.99
mmu-miR-760-3p	1.42	5.08E-01	> 0.99
mmu-miR-200a-3p	-1.21	5.08E-01	> 0.99
mmu-miR-744-3p	-1.20	5.09E-01	> 0.99
mmu-miR-376a-3p	-1.33	5.20E-01	> 0.99
mmu-miR-652-3p	-1.22	5.21E-01	> 0.99
mmu-miR-384-5p	-1.66	5.23E-01	> 0.99
mmu-miR-15a-5p	-1.20	5.26E-01	> 0.99
mmu-miR-484	1.21	5.27E-01	> 0.99
mmu-miR-10a-5p	-1.18	5.27E-01	> 0.99
mmu-miR-30c-1-3p	1.35	5.27E-01	> 0.99
mmu-miR-1907	-1.55	5.29E-01	> 0.99
mmu-miR-496a-3p	-1.50	5.29E-01	> 0.99
mmu-miR-380-3p	-1.32	5.30E-01	> 0.99
mmu-miR-29b-3p	-1.21	5.34E-01	> 0.99
mmu-miR-183-5p	-1.26	5.34E-01	> 0.99
mmu-miR-668-3p	1.47	5.41E-01	> 0.99
mmu-miR-1981-5p	1.30	5.41E-01	> 0.99
mmu-let-7g-5p	-1.21	5.42E-01	> 0.99
mmu-miR-681	-1.28	5.45E-01	> 0.99
mmu-miR-196a-5p	1.42	5.46E-01	> 0.99
mmu-miR-26b-5p	-1.22	5.46E-01	> 0.99
mmu-miR-20a-5p	1.21	5.47E-01	> 0.99
mmu-miR-378a-3p	-1.18	5.49E-01	> 0.99
mmu-miR-1839-5p	-1.24	5.51E-01	> 0.99
mmu-miR-467c-5p	-1.40	5.57E-01	> 0.99
mmu-miR-1930-5p	-1.24	5.58E-01	> 0.99
mmu-miR-329-3p	-1.32	5.59E-01	> 0.99
mmu-miR-872-5p	-1.23	5.61E-01	> 0.99
mmu-miR-466g	1.14	5.62E-01	> 0.99
mmu-miR-181b-5p	-1.23	5.63E-01	> 0.99
mmu-miR-1961	-1.16	5.64E-01	> 0.99
mmu-miR-677-5p	-1.56	5.67E-01	> 0.99
mmu-miR-320-3p	-1.14	5.68E-01	> 0.99
mmu-miR-379-5p	-1.28	5.71E-01	> 0.99
mmu-miR-26a-5p	-1.22	5.72E-01	> 0.99
mmu-miR-676-5p	1.40	5.73E-01	> 0.99
mmu-miR-1191	-1.22	5.75E-01	> 0.99
mmu-miR-200b-3p	-1.20	5.80E-01	> 0.99
mmu-miR-181a-5p	-1.19	5.81E-01	> 0.99
mmu-miR-224-5p	-1.22	5.81E-01	> 0.99
mmu-miR-1934-5p	-1.47	5.81E-01	> 0.99
mmu-miR-155-5p	-1.22	5.83E-01	> 0.99
mmu-miR-1935	1.54	5.86E-01	> 0.99
mmu-let-7e-3p	-1.25	5.86E-01	> 0.99
mmu-miR-744-5p	-1.12	5.87E-01	> 0.99
mmu-miR-137-3p	1.27	5.92E-01	> 0.99
mmu-miR-376b-3p	-1.28	5.94E-01	> 0.99
mmu-miR-291b-5p	1.23	5.95E-01	> 0.99
mmu-miR-297c-5p	-1.28	5.95E-01	> 0.99
mmu-miR-433-5p	-1.33	5.95E-01	> 0.99
mmu-miR-365-3p	-1.18	5.99E-01	> 0.99
mmu-miR-340-3p	-1.18	5.99E-01	> 0.99
mmu-miR-142-3p	-1.17	6.00E-01	> 0.99
mmu-miR-20a-3p	1.22	6.01E-01	> 0.99
mmu-miR-10b-5p	-1.16	6.03E-01	> 0.99
mmu-miR-325-3p	-1.18	6.03E-01	> 0.99
mmu-miR-27a-3p	-1.16	6.04E-01	> 0.99

mmu-miR-501-3p	1.19	6.08E-01	> 0.99
mmu-miR-124-3p	1.19	6.10E-01	> 0.99
mmu-miR-92a-2-5p	1.30	6.10E-01	> 0.99
mmu-miR-207	1.31	6.15E-01	> 0.99
mmu-let-7b-5p	-1.17	6.16E-01	> 0.99
mmu-miR-449a-5p	1.24	6.18E-01	> 0.99
mmu-miR-543-3p	-1.31	6.19E-01	> 0.99
mmu-miR-219-1-3p	1.40	6.24E-01	> 0.99
mmu-miR-145a-3p	-1.19	6.24E-01	> 0.99
mmu-miR-15b-5p	-1.19	6.27E-01	> 0.99
mmu-miR-762	-1.11	6.27E-01	> 0.99
mmu-miR-106b-3p	-1.18	6.30E-01	> 0.99
mmu-miR-135a-5p	-1.19	6.31E-01	> 0.99
mmu-miR-466j	1.51	6.35E-01	> 0.99
mmu-miR-130b-5p	-1.20	6.38E-01	> 0.99
mmu-miR-191-3p	1.18	6.40E-01	> 0.99
mmu-miR-182-5p	-1.15	6.41E-01	> 0.99
mmu-miR-206-3p	1.33	6.42E-01	> 0.99
mmu-miR-139-5p	-1.14	6.45E-01	> 0.99
mmu-miR-200a-5p	-1.15	6.47E-01	> 0.99
mmu-miR-200b-5p	-1.16	6.51E-01	> 0.99
mmu-miR-704	1.32	6.56E-01	> 0.99
mmu-miR-30d-3p	-1.17	6.56E-01	> 0.99
mmu-miR-411-3p	-1.11	6.57E-01	> 0.99
mmu-miR-1186a	-1.33	6.57E-01	> 0.99
mmu-miR-31-5p	-1.14	6.57E-01	> 0.99
mmu-miR-340-5p	-1.25	6.58E-01	> 0.99
mmu-miR-590-3p	-1.13	6.61E-01	> 0.99
mmu-miR-351-5p	-1.17	6.64E-01	> 0.99
mmu-miR-539-5p	1.41	6.67E-01	> 0.99
mmu-let-7d-3p	1.20	6.68E-01	> 0.99
mmu-miR-331-3p	1.22	6.71E-01	> 0.99
mmu-miR-669l-5p	-1.33	6.72E-01	> 0.99
mmu-miR-1967	-1.15	6.73E-01	> 0.99
mmu-miR-434-5p	-1.20	6.73E-01	> 0.99
mmu-miR-708-3p	-1.32	6.76E-01	> 0.99
mmu-miR-199a-3p	-1.14	6.79E-01	> 0.99
mmu-miR-337-5p	1.26	6.83E-01	> 0.99
mmu-miR-322-5p	-1.14	6.85E-01	> 0.99
mmu-miR-140-5p	-1.14	6.85E-01	> 0.99
mmu-miR-466d-5p	-1.49	6.86E-01	> 0.99
mmu-miR-19b-1-5p	-1.20	6.87E-01	> 0.99
mmu-miR-29a-3p	-1.14	6.89E-01	> 0.99
mmu-miR-203-3p	-1.12	6.90E-01	> 0.99
mmu-miR-425-5p	-1.13	6.90E-01	> 0.99
mmu-miR-450b-5p	-1.57	6.94E-01	> 0.99

mmu-miR-338-3p	-1.15	6.96E-01	> 0.99
mmu-miR-342-3p	-1.13	7.00E-01	> 0.99
mmu-miR-1951	1.18	7.04E-01	> 0.99
mmu-miR-1247-5p	-1.27	7.05E-01	> 0.99
mmu-miR-1198-5p	-1.12	7.07E-01	> 0.99
mmu-miR-141-3p	-1.12	7.08E-01	> 0.99
mmu-miR-203-5p	-1.10	7.11E-01	> 0.99
mmu-miR-145a-5p	-1.16	7.11E-01	> 0.99
mmu-miR-574-3p	-1.14	7.11E-01	> 0.99
mmu-miR-362-3p	1.16	7.11E-01	> 0.99
mmu-let-7a-5p	-1.12	7.12E-01	> 0.99
mmu-miR-9-3p	1.25	7.14E-01	> 0.99
mmu-miR-199a-5p	-1.12	7.17E-01	> 0.99
mmu-miR-377-3p	1.15	7.18E-01	> 0.99
mmu-miR-1839-3p	-1.13	7.18E-01	> 0.99
mmu-miR-425-3p	-1.13	7.19E-01	> 0.99
mmu-miR-466c-5p	1.21	7.24E-01	> 0.99
mmu-miR-125b-5p	-1.11	7.27E-01	> 0.99
mmu-miR-500-3p	1.13	7.30E-01	> 0.99
mmu-miR-191-5p	-1.13	7.31E-01	> 0.99
mmu-miR-742-5p	1.16	7.31E-01	> 0.99
mmu-miR-29a-5p	-1.15	7.32E-01	> 0.99
mmu-miR-199b-5p	-1.12	7.35E-01	> 0.99
mmu-miR-212-5p	1.17	7.36E-01	> 0.99
mmu-miR-300-3p	1.16	7.38E-01	> 0.99
mmu-miR-1949	1.09	7.41E-01	> 0.99
mmu-miR-210-3p	1.10	7.41E-01	> 0.99
mmu-miR-183-3p	1.12	7.43E-01	> 0.99
mmu-miR-411-5p	1.14	7.44E-01	> 0.99
mmu-miR-374b-5p	-1.08	7.50E-01	> 0.99
mmu-miR-125a-5p	-1.10	7.52E-01	> 0.99
mmu-miR-190b-5p	-1.13	7.52E-01	> 0.99
mmu-miR-2137	1.09	7.53E-01	> 0.99
mmu-miR-804	1.15	7.53E-01	> 0.99
mmu-miR-181d-5p	-1.11	7.55E-01	> 0.99
mmu-miR-16-5p	-1.10	7.56E-01	> 0.99
mmu-miR-19a-3p	1.10	7.58E-01	> 0.99
mmu-miR-433-3p	-1.17	7.58E-01	> 0.99
mmu-miR-133b-3p	-1.12	7.59E-01	> 0.99
mmu-miR-34b-5p	-1.16	7.60E-01	> 0.99
mmu-miR-196b-5p	1.23	7.61E-01	> 0.99
mmu-miR-30e-5p	-1.10	7.61E-01	> 0.99
mmu-miR-504-5p	-1.52	7.61E-01	> 0.99
mmu-let-7c-5p	-1.09	7.62E-01	> 0.99
mmu-miR-200c-3p	-1.09	7.64E-01	> 0.99
mmu-miR-1927	1.09	7.64E-01	> 0.99

mmu-miR-381-3p	-1.13	7.66E-01	> 0.99
mmu-miR-30c-2-3p	1.15	7.66E-01	> 0.99
mmu-miR-1306-3p	-1.20	7.69E-01	> 0.99
mmu-miR-136-5p	1.14	7.76E-01	> 0.99
mmu-miR-192-5p	-1.09	7.77E-01	> 0.99
mmu-miR-466f-5p	1.13	7.77E-01	> 0.99
mmu-miR-144-3p	1.12	7.79E-01	> 0.99
mmu-miR-301a-3p	1.10	7.79E-01	> 0.99
mmu-miR-335-3p	-1.10	7.80E-01	> 0.99
mmu-miR-1982-5p	-1.15	7.80E-01	> 0.99
mmu-miR-541-5p	-1.08	7.82E-01	> 0.99
mmu-miR-122-5p	-1.17	7.83E-01	> 0.99
mmu-let-7i-3p	-1.10	7.86E-01	> 0.99
mmu-miR-423-3p	-1.07	7.86E-01	> 0.99
mmu-miR-20b-5p	1.19	7.89E-01	> 0.99
mmu-miR-149-5p	-1.11	7.90E-01	> 0.99
mmu-miR-467h	-1.14	7.94E-01	> 0.99
mmu-miR-495-3p	-1.07	7.97E-01	> 0.99
mmu-miR-25-3p	-1.07	8.00E-01	> 0.99
mmu-miR-99b-5p	-1.09	8.02E-01	> 0.99
mmu-miR-335-5p	-1.10	8.02E-01	> 0.99
mmu-miR-99b-3p	-1.19	8.04E-01	> 0.99
mmu-miR-674-5p	-1.08	8.05E-01	> 0.99
mmu-miR-682	-1.17	8.07E-01	> 0.99
mmu-miR-101a-3p	-1.08	8.08E-01	> 0.99
mmu-miR-193b-3p	1.06	8.14E-01	> 0.99
mmu-miR-1196-5p	1.10	8.16E-01	> 0.99
mmu-miR-376c-5p	-1.20	8.17E-01	> 0.99
mmu-let-7e-5p	1.08	8.17E-01	> 0.99
mmu-miR-154-3p	-1.10	8.18E-01	> 0.99
mmu-miR-30a-5p	-1.08	8.19E-01	> 0.99
mmu-miR-195a-5p	-1.09	8.20E-01	> 0.99
mmu-miR-671-5p	-1.06	8.22E-01	> 0.99
mmu-miR-222-3p	-1.08	8.22E-01	> 0.99
mmu-let-7a-1-3p	1.07	8.22E-01	> 0.99
mmu-miR-466f-3p	1.09	8.26E-01	> 0.99
mmu-miR-615-3p	-1.17	8.27E-01	> 0.99
mmu-miR-540-5p	1.11	8.29E-01	> 0.99
mmu-miR-669n	-1.07	8.29E-01	> 0.99
mmu-miR-188-5p	1.13	8.30E-01	> 0.99
mmu-miR-100-5p	-1.06	8.36E-01	> 0.99
mmu-miR-409-5p	-1.16	8.38E-01	> 0.99
mmu-miR-21a-5p	-1.07	8.40E-01	> 0.99
mmu-miR-339-3p	1.06	8.45E-01	> 0.99
mmu-miR-186-3p	1.06	8.45E-01	> 0.99
mmu-miR-93-3p	-1.08	8.48E-01	> 0.99

mmu-miR-133a-5p	1.11	8.54E-01	> 0.99
mmu-let-7i-5p	1.06	8.56E-01	> 0.99
mmu-miR-1946a	1.05	8.57E-01	> 0.99
mmu-miR-21a-3p	-1.04	8.58E-01	> 0.99
mmu-miR-24-3p	-1.06	8.59E-01	> 0.99
mmu-miR-497-5p	-1.05	8.60E-01	> 0.99
mmu-miR-1895	-1.05	8.60E-01	> 0.99
mmu-miR-125a-3p	1.06	8.61E-01	> 0.99
mmu-miR-466a-3p	1.09	8.62E-01	> 0.99
mmu-miR-221-3p	-1.04	8.64E-01	> 0.99
mmu-miR-542-5p	-1.06	8.66E-01	> 0.99
mmu-miR-467b-3p	-1.07	8.69E-01	> 0.99
mmu-miR-107-3p	1.05	8.70E-01	> 0.99
mmu-miR-154-5p	-1.05	8.72E-01	> 0.99
mmu-miR-99a-5p	-1.06	8.73E-01	> 0.99
mmu-miR-532-3p	-1.05	8.73E-01	> 0.99
mmu-miR-193a-3p	1.09	8.74E-01	> 0.99
mmu-miR-192-3p	-1.05	8.80E-01	> 0.99
mmu-miR-32-5p	1.05	8.81E-01	> 0.99
mmu-miR-146b-5p	1.04	8.81E-01	> 0.99
mmu-miR-429-3p	1.05	8.82E-01	> 0.99
mmu-miR-455-5p	1.05	8.84E-01	> 0.99
mmu-miR-106b-5p	1.05	8.84E-01	> 0.99
mmu-miR-204-5p	-1.06	8.84E-01	> 0.99
mmu-miR-421-3p	-1.05	8.85E-01	> 0.99
mmu-miR-17-3p	-1.03	8.85E-01	> 0.99
mmu-miR-23a-3p	-1.05	8.86E-01	> 0.99
mmu-miR-324-3p	-1.04	8.86E-01	> 0.99
mmu-miR-223-3p	-1.04	8.87E-01	> 0.99
mmu-miR-674-3p	-1.05	8.87E-01	> 0.99
mmu-miR-128-3p	-1.05	8.88E-01	> 0.99
mmu-miR-194-2-3p	-1.03	8.88E-01	> 0.99
mmu-let-7f-5p	-1.04	8.90E-01	> 0.99
mmu-miR-27b-3p	-1.05	8.93E-01	> 0.99
mmu-let-7d-5p	-1.04	8.96E-01	> 0.99
mmu-miR-487b-3p	-1.08	8.96E-01	> 0.99
mmu-miR-34c-5p	1.03	9.00E-01	> 0.99
mmu-miR-28a-5p	-1.04	9.00E-01	> 0.99
mmu-miR-410-3p	-1.04	9.02E-01	> 0.99
mmu-miR-451a	1.04	9.06E-01	> 0.99
mmu-miR-300-5p	1.06	9.07E-01	> 0.99
mmu-miR-431-5p	1.05	9.07E-01	> 0.99
mmu-miR-379-3p	1.04	9.08E-01	> 0.99
mmu-miR-670-5p	-1.06	9.09E-01	> 0.99
mmu-miR-106a-5p	1.04	9.09E-01	> 0.99
mmu-miR-10a-3p	-1.06	9.12E-01	> 0.99

mmu-miR-490-3p	-1.03	9.17E-01	> 0.99
mmu-miR-324-5p	1.04	9.17E-01	> 0.99
mmu-miR-770-5p	-1.05	9.19E-01	> 0.99
mmu-miR-872-3p	1.04	9.20E-01	> 0.99
mmu-miR-345-5p	1.03	9.24E-01	> 0.99
mmu-miR-345-3p	1.05	9.25E-01	> 0.99
mmu-miR-708-5p	1.04	9.26E-01	> 0.99
mmu-miR-450b-3p	1.06	9.26E-01	> 0.99
mmu-miR-532-5p	1.03	9.29E-01	> 0.99
mmu-miR-1a-3p	-1.03	9.32E-01	> 0.99
mmu-miR-669a-5p	-1.05	9.34E-01	> 0.99
mmu-miR-20b-3p	-1.06	9.36E-01	> 0.99
mmu-miR-129-1-3p	1.04	9.37E-01	> 0.99
mmu-miR-9-5p	1.03	9.40E-01	> 0.99
mmu-miR-185-5p	-1.03	9.41E-01	> 0.99
mmu-miR-148b-3p	-1.02	9.43E-01	> 0.99
mmu-let-7b-3p	1.03	9.49E-01	> 0.99
mmu-miR-361-5p	1.02	9.50E-01	> 0.99
mmu-miR-19b-3p	1.02	9.51E-01	> 0.99
mmu-miR-22-3p	-1.02	9.52E-01	> 0.99
mmu-miR-877-5p	-1.06	9.53E-01	> 0.99
mmu-miR-134-5p	-1.03	9.53E-01	> 0.99
mmu-miR-126-3p	-1.02	9.53E-01	> 0.99
mmu-miR-130b-3p	1.02	9.56E-01	> 0.99
mmu-miR-467e-5p	-1.03	9.57E-01	> 0.99
mmu-miR-152-3p	-1.02	9.57E-01	> 0.99
mmu-miR-15b-3p	-1.02	9.57E-01	> 0.99
mmu-miR-337-3p	1.03	9.59E-01	> 0.99
mmu-miR-363-3p	-1.03	9.59E-01	> 0.99
mmu-miR-141-5p	-1.02	9.60E-01	> 0.99
mmu-miR-592-5p	1.03	9.60E-01	> 0.99
mmu-miR-93-5p	1.02	9.60E-01	> 0.99
mmu-miR-143-3p	1.02	9.61E-01	> 0.99
mmu-miR-98-5p	1.01	9.62E-01	> 0.99
mmu-miR-148a-3p	1.02	9.63E-01	> 0.99
mmu-miR-30a-3p	1.01	9.64E-01	> 0.99
mmu-miR-186-5p	1.01	9.64E-01	> 0.99
mmu-miR-101b-3p	1.01	9.66E-01	> 0.99
mmu-miR-328-3p	-1.01	9.71E-01	> 0.99
mmu-miR-501-5p	1.02	9.74E-01	> 0.99
mmu-miR-467a-5p	1.01	9.74E-01	> 0.99
mmu-miR-467d-5p	1.01	9.76E-01	> 0.99
mmu-miR-758-3p	-1.01	9.78E-01	> 0.99
mmu-miR-218-5p	1.01	9.79E-01	> 0.99
mmu-miR-103-3p	1.01	9.80E-01	> 0.99
mmu-miR-299a-3p	-1.01	9.81E-01	> 0.99
mmu-miR-103-2-5p	-1.01	9.83E-01	> 0.99
mmu-let-7f-1-3p	1.01	9.83E-01	> 0.99
mmu-miR-219-5p	-1.01	9.86E-01	> 0.99
mmu-miR-1968-5p	1.01	9.90E-01	> 0.99
mmu-miR-23b-3p	-1.00	9.90E-01	> 0.99
mmu-miR-467d-3p	1.00	> 0.99	> 0.99
mmu-miR-342-5p	1.00	> 0.99	> 0.99
mmu-miR-22-5p	1.00	> 0.99	> 0.99

Supplementary Table 3. Intestinal miRNAs altered in the 20 weeks study. miRNAs differentially expressed after the consumption of a high-fat diet (HFD) for 20 weeks, in male C57BL/6 mice. [a] In bold, microRNAs with significant change, unpaired T-test, $p < 0.05$. [b] 20 weeks HFD *vs.* control. [c] Benjamini and Hochberg corrected values.

miRNA[a]	Fold Change[b]	P-value	P (Benjamini-Hochberg)[c]
**mmu-let-7i**	-2.24	8.40E-07	1.18E-02
**mmu-miR-542-5p**	-2.55	1.27E-05	1.24E-02
**mmu-miR-21**	-2.46	1.49E-05	1.24E-02
**mmu-miR-154**	-2.73	1.77E-05	1.24E-02
**mmu-miR-1897-5p**	7.63	3.87E-05	1.25E-02
**mmu-miR-23a**	-1.90	4.22E-05	1.27E-02
**mmu-miR-133a**	-2.75	5.49E-05	1.27E-02
**mmu-miR-598**	-2.50	6.01E-05	1.30E-02
**mmu-miR-133b**	-2.62	7.40E-05	1.30E-02
**mmu-miR-9**	-2.65	8.73E-05	1.35E-02
**mmu-miR-199a-3p**	-2.25	9.89E-05	1.45E-02
**mmu-miR-143**	-2.11	9.92E-05	1.45E-02
**mmu-miR-103**	1.67	1.47E-04	1.45E-02
**mmu-miR-425**	-2.00	1.48E-04	1.45E-02
**mmu-miR-130b**	-3.18	1.70E-04	1.53E-02
**mmu-miR-486**	-4.57	1.78E-04	1.53E-02
**mmu-miR-23a***	-2.49	2.18E-04	1.53E-02
**mmu-miR-27b**	-1.96	2.21E-04	1.53E-02
**mmu-miR-136**	-2.80	2.22E-04	1.54E-02
**mmu-miR-140***	-1.67	2.57E-04	1.54E-02
**mmu-miR-148b**	-1.94	2.63E-04	1.59E-02
**mmu-miR-384-5p**	-4.26	2.64E-04	1.59E-02
**mmu-miR-146a**	-2.60	2.67E-04	1.65E-02
**mmu-miR-449c**	11.05	3.05E-04	1.68E-02
**mmu-miR-342-5p**	-2.07	3.15E-04	1.68E-02
**mmu-miR-1894-5p**	-3.90	3.18E-04	1.70E-02
**mmu-miR-217**	-3.76	3.24E-04	1.82E-02
**mmu-miR-26b**	-3.19	3.30E-04	1.82E-02
**mmu-miR-185**	-2.51	3.62E-04	1.82E-02
**mmu-miR-26a**	-2.75	3.65E-04	1.84E-02
**mmu-miR-1**	-2.67	3.67E-04	1.85E-02
**mmu-miR-29b**	-2.28	3.88E-04	1.87E-02
**mmu-miR-151-3p**	-2.03	4.10E-04	1.93E-02
**mmu-miR-147**	-3.60	4.39E-04	2.15E-02
**mmu-miR-16**	-2.79	5.09E-04	2.24E-02
**mmu-miR-15a**	-1.99	5.78E-04	2.46E-02
**mmu-miR-127**	-3.79	5.79E-04	2.46E-02
**mmu-miR-125b-5p**	-2.17	5.92E-04	2.49E-02
**mmu-miR-669e**	5.84	6.06E-04	2.51E-02
**mmu-miR-191**	-1.68	6.47E-04	2.62E-02
**mmu-miR-7b**	-2.41	6.86E-04	2.69E-02
**mmu-miR-181a**	-2.26	7.63E-04	2.69E-02
**mmu-miR-145**	-2.26	7.64E-04	2.72E-02
**mmu-miR-138**	-2.35	7.98E-04	2.75E-02
**mmu-miR-1894-3p**	2.54	7.99E-04	2.75E-02
mmu-miR-467e	-2.45	1.12E-03	2.75E-02
mmu-miR-22	-1.87	1.12E-03	2.75E-02
mmu-miR-33	-3.67	1.16E-03	2.75E-02
mmu-miR-27a	-1.99	1.20E-03	2.77E-02
mmu-miR-411	-2.51	1.29E-03	2.80E-02
mmu-miR-337-3p	-3.84	1.34E-03	2.80E-02
mmu-miR-363-3p	-2.18	1.35E-03	2.89E-02
mmu-miR-10a	-2.17	1.47E-03	2.89E-02
mmu-miR-29c	-1.71	1.48E-03	2.95E-02
mmu-miR-666-3p	-3.26	1.50E-03	2.97E-02
mmu-miR-183	-2.20	1.50E-03	2.98E-02
mmu-miR-503	-3.36	1.51E-03	2.98E-02
mmu-miR-96	-2.14	1.62E-03	2.98E-02
mmu-miR-29a*	-1.67	1.65E-03	2.98E-02
mmu-miR-142-5p	-3.24	1.69E-03	2.98E-02
mmu-miR-125a-5p	-1.93	1.76E-03	3.05E-02
mmu-miR-369-5p	-1.67	1.77E-03	3.08E-02
mmu-let-7c	-1.65	1.86E-03	3.15E-02
mmu-miR-148a	-2.09	1.87E-03	3.15E-02
mmu-miR-20b	-5.04	1.96E-03	3.15E-02
mmu-miR-322	-2.25	2.20E-03	3.15E-02
mmu-miR-301b	-2.04	2.21E-03	3.18E-02
mmu-miR-106a	-1.92	2.22E-03	3.19E-02
mmu-miR-31*	1.62	2.23E-03	3.20E-02
mmu-miR-30c	-1.67	2.39E-03	3.29E-02
mmu-miR-1966	3.89	2.42E-03	3.40E-02
mmu-miR-467a	-2.32	2.45E-03	3.40E-02
mmu-miR-144	-5.23	2.47E-03	3.41E-02
mmu-let-7e	-2.04	2.53E-03	3.47E-02
mmu-miR-142-3p	-2.63	2.55E-03	3.47E-02
mmu-miR-704	-2.41	2.67E-03	3.47E-02
mmu-miR-101b	-1.97	2.70E-03	3.47E-02
mmu-miR-1947	-4.05	2.84E-03	3.47E-02
mmu-miR-362-3p	-1.75	2.94E-03	3.47E-02
mmu-miR-130a	-1.83	2.96E-03	3.47E-02
mmu-miR-1968	3.99	3.02E-03	3.47E-02

mmu-miR-92b	-3.51	3.29E-03	3.59E-02
mmu-miR-190	-2.01	3.32E-03	3.63E-02
mmu-miR-326	-1.82	3.35E-03	3.64E-02
mmu-miR-350	-2.23	3.42E-03	3.67E-02
mmu-miR-101a	-1.69	3.49E-03	3.74E-02
mmu-miR-24	-1.83	3.56E-03	3.74E-02
mmu-miR-339-3p	-4.24	3.70E-03	3.82E-02
mmu-miR-449a	-2.47	4.18E-03	3.94E-02
mmu-miR-377	-4.33	4.93E-03	3.97E-02
mmu-miR-139-3p	-2.59	4.94E-03	3.99E-02
mmu-miR-99b	-1.85	5.05E-03	3.99E-02
mmu-miR-374	-1.87	5.12E-03	3.99E-02
mmu-miR-182	-1.84	5.64E-03	3.99E-02
mmu-miR-107	-1.58	5.67E-03	3.99E-02
mmu-miR-497	-1.63	5.78E-03	4.02E-02
mmu-miR-345-3p	3.11	5.95E-03	4.03E-02
mmu-miR-466j	2.27	5.96E-03	4.03E-02
mmu-miR-467b	-2.52	6.10E-03	4.23E-02
mmu-miR-99a	-2.57	6.12E-03	4.23E-02
mmu-miR-93*	-2.16	6.23E-03	4.46E-02
mmu-miR-501-3p	-1.94	6.36E-03	4.50E-02
mmu-miR-19b	-1.69	6.41E-03	4.52E-02
mmu-miR-1898	4.40	6.71E-03	4.52E-02
mmu-miR-15b	-1.94	6.72E-03	4.52E-02
mmu-miR-488	-4.23	6.93E-03	4.52E-02
mmu-miR-17	-1.85	7.02E-03	4.53E-02
mmu-miR-200a	-1.57	7.11E-03	4.68E-02
mmu-miR-543	-5.57	7.30E-03	4.68E-02
mmu-miR-221	-1.59	7.33E-03	4.69E-02
mmu-miR-199a-5p	-1.62	7.37E-03	4.79E-02
mmu-miR-674	1.53	7.59E-03	4.96E-02
mmu-miR-376b	-3.53	7.72E-03	5.02E-02
mmu-miR-339-5p	-1.68	7.96E-03	5.08E-02
mmu-miR-140	-2.13	8.09E-03	5.10E-02
mmu-miR-369-3p	-2.48	8.16E-03	5.10E-02
mmu-miR-145*	-1.80	8.30E-03	5.22E-02
mmu-miR-195	-1.71	8.40E-03	5.22E-02
mmu-miR-1306	-2.21	8.48E-03	5.28E-02
mmu-miR-124	-2.20	8.78E-03	5.35E-02
mmu-miR-450b-5p	-2.79	9.19E-03	5.37E-02
mmu-miR-219-5p	-2.07	9.22E-03	5.37E-02
mmu-miR-30d	-1.73	9.31E-03	5.40E-02
mmu-miR-192	-1.59	9.60E-03	5.48E-02
mmu-miR-214	-1.88	9.70E-03	5.71E-02
mmu-miR-330	-2.34	9.80E-03	5.74E-02
mmu-miR-200b	-1.69	9.80E-03	5.74E-02

mmu-miR-181c	-2.10	9.96E-03	5.91E-02
mmu-miR-299	-2.04	1.00E-02	5.91E-02
mmu-miR-139-5p	-1.63	1.05E-02	5.97E-02
mmu-miR-186	-1.82	1.07E-02	5.98E-02
mmu-miR-324-5p	-1.87	1.08E-02	5.98E-02
mmu-miR-325	-1.84	1.09E-02	6.32E-02
mmu-miR-153	-4.80	1.12E-02	6.36E-02
mmu-miR-193	-2.46	1.13E-02	6.38E-02
mmu-miR-1895	2.56	1.16E-02	6.57E-02
mmu-miR-328	-2.00	1.20E-02	6.57E-02
mmu-miR-126-3p	-1.39	1.22E-02	6.69E-02
mmu-miR-223	-1.91	1.25E-02	6.70E-02
mmu-miR-138-1*	-1.68	1.25E-02	6.70E-02
mmu-miR-1946a	2.40	1.26E-02	6.92E-02
mmu-miR-338-5p	-2.87	1.26E-02	7.01E-02
mmu-miR-708	-3.02	1.27E-02	7.39E-02
mmu-miR-434-3p	-2.19	1.29E-02	7.40E-02
mmu-miR-351	-2.38	1.30E-02	7.44E-02
mmu-miR-137	-1.77	1.30E-02	7.49E-02
mmu-miR-335-5p	-2.53	1.38E-02	7.51E-02
mmu-miR-380-3p	-2.58	1.39E-02	7.53E-02
mmu-miR-674*	1.58	1.47E-02	7.60E-02
mmu-miR-100	-2.75	1.50E-02	7.74E-02
mmu-miR-669a-5p	-3.61	1.51E-02	7.85E-02
mmu-miR-23b	-1.53	1.52E-02	7.85E-02
mmu-miR-1907	2.46	1.54E-02	8.04E-02
mmu-miR-423-3p	-1.70	1.55E-02	8.43E-02
mmu-miR-299*	-2.48	1.61E-02	8.51E-02
mmu-miR-592	-2.84	1.62E-02	8.68E-02
mmu-miR-93	-1.61	1.63E-02	8.86E-02
mmu-miR-149	-2.22	1.68E-02	8.86E-02
mmu-let-7f	-2.19	1.75E-02	9.15E-02
mmu-miR-345-5p	-1.90	1.81E-02	9.27E-02
mmu-miR-193b	-2.58	1.83E-02	9.27E-02
mmu-miR-188-5p	-2.04	1.84E-02	9.37E-02
mmu-miR-19a	-1.79	1.89E-02	9.37E-02
mmu-miR-218	-1.44	1.90E-02	1.01E-01
mmu-miR-1196	3.21	1.93E-02	1.02E-01
mmu-miR-301a	-3.89	1.97E-02	1.03E-01
mmu-miR-181b	-2.00	2.00E-02	1.05E-01
mmu-let-7b	-1.45	2.00E-02	1.06E-01
mmu-miR-874	-2.04	2.02E-02	1.09E-01
mmu-miR-27a*	-1.85	2.16E-02	1.10E-01
mmu-miR-324-3p	-1.75	2.19E-02	1.10E-01
mmu-miR-296-5p	-3.51	2.20E-02	1.10E-01
mmu-miR-467c	-3.24	2.28E-02	1.12E-01

mmu-miR-106b	-1.51	2.32E-02	1.12E-01
mmu-miR-9*	-2.06	2.35E-02	1.12E-01
mmu-miR-18a	-1.59	2.50E-02	1.12E-01
mmu-miR-200c	-1.39	2.52E-02	1.14E-01
mmu-miR-127*	-2.44	2.54E-02	1.14E-01
mmu-miR-760-3p	-3.10	2.64E-02	1.15E-01
mmu-miR-30b	-1.56	2.65E-02	1.18E-01
mmu-miR-450a	-3.73	2.71E-02	1.19E-01
mmu-miR-330*	2.30	2.74E-02	1.19E-01
mmu-miR-106b*	1.30	2.84E-02	1.21E-01
mmu-miR-468	2.13	2.90E-02	1.22E-01
mmu-miR-466a-3p	-4.06	3.07E-02	1.26E-01
mmu-miR-101a*	-1.29	3.08E-02	1.26E-01
mmu-miR-365	-1.82	3.12E-02	1.26E-01
mmu-miR-150	-2.39	3.16E-02	1.28E-01
mmu-miR-141	-1.51	3.18E-02	1.28E-01
mmu-miR-434-5p	-1.84	3.20E-02	1.29E-01
mmu-miR-152	-1.49	3.32E-02	1.33E-01
mmu-miR-652	-1.68	3.38E-02	1.34E-01
mmu-miR-132	-1.29	3.40E-02	1.34E-01
mmu-miR-877	-1.73	3.50E-02	1.34E-01
mmu-miR-342-3p	-1.49	3.69E-02	1.34E-01
mmu-miR-715	2.24	3.74E-02	1.38E-01
mmu-miR-18a*	-1.85	3.83E-02	1.38E-01
mmu-miR-204	-1.89	3.94E-02	1.38E-01
mmu-miR-362-5p	-1.77	3.95E-02	1.39E-01
mmu-miR-451	-3.75	4.10E-02	1.39E-01
mmu-miR-29a	-1.70	4.17E-02	1.39E-01
mmu-miR-455*	-5.57	4.19E-02	1.44E-01
mmu-miR-1949	1.25	4.26E-02	1.45E-01
mmu-miR-184	-1.50	4.27E-02	1.46E-01
mmu-miR-99b*	1.59	4.60E-02	1.48E-01
mmu-miR-20a*	-2.22	4.69E-02	1.49E-01
mmu-miR-484	-1.42	4.77E-02	1.50E-01
mmu-miR-29c*	-1.62	5.09E-02	1.52E-01
mmu-miR-762	1.89	5.19E-02	1.56E-01
mmu-miR-125a-3p	-2.44	5.23E-02	1.60E-01
mmu-miR-129-5p	-2.01	5.36E-02	1.61E-01
mmu-miR-1900	1.34	5.36E-02	1.64E-01
mmu-miR-466f	2.52	5.39E-02	1.66E-01
mmu-miR-710	-1.74	5.41E-02	1.66E-01
mmu-miR-669b	2.09	5.50E-02	1.67E-01
mmu-miR-181d	-2.44	5.55E-02	1.71E-01
mmu-let-7d*	1.35	5.77E-02	1.71E-01
mmu-miR-582-5p	-1.38	5.85E-02	1.71E-01
mmu-miR-1959	-1.64	5.89E-02	1.73E-01

mmu-miR-673-5p	-2.49	6.08E-02	1.75E-01
mmu-miR-203*	-1.82	6.31E-02	1.75E-01
mmu-miR-298	-2.39	6.32E-02	1.79E-01
mmu-miR-532-5p	-1.56	6.47E-02	1.79E-01
mmu-miR-155	-2.00	6.56E-02	1.79E-01
mmu-miR-212-3p	-1.53	6.80E-02	1.81E-01
mmu-miR-205	-2.00	6.87E-02	1.85E-01
mmu-miR-500	-1.59	6.91E-02	1.87E-01
mmu-miR-21*	-1.42	6.94E-02	1.88E-01
mmu-miR-744	-1.68	6.99E-02	1.90E-01
mmu-miR-615-3p	-1.93	7.20E-02	1.95E-01
mmu-let-7i*	-1.38	7.26E-02	2.05E-01
mmu-miR-187	-1.99	7.28E-02	2.05E-01
mmu-miR-296-3p	1.60	7.36E-02	2.06E-01
mmu-miR-134	2.27	7.42E-02	2.14E-01
mmu-miR-25	-1.62	7.76E-02	2.16E-01
mmu-miR-532-3p	-1.36	7.84E-02	2.18E-01
mmu-miR-34c	-1.80	8.18E-02	2.19E-01
mmu-miR-1930	1.67	8.33E-02	2.21E-01
mmu-miR-1961	1.38	8.58E-02	2.23E-01
mmu-miR-574-3p	-1.54	8.79E-02	2.32E-01
mmu-miR-425*	-1.58	8.89E-02	2.37E-01
mmu-let-7b*	-1.76	9.27E-02	2.38E-01
mmu-miR-340-3p	-1.73	9.30E-02	2.38E-01
mmu-miR-34b-5p	-2.50	9.39E-02	2.38E-01
mmu-miR-431	-1.83	9.66E-02	2.39E-01
mmu-miR-320	-1.40	9.68E-02	2.42E-01
mmu-miR-222	-2.10	9.69E-02	2.44E-01
mmu-miR-379	-1.74	9.85E-02	2.46E-01
mmu-miR-300	-1.85	1.00E-01	2.51E-01
mmu-miR-31	-1.22	1.00E-01	2.53E-01
mmu-miR-694	-25.64	1.03E-01	2.53E-01
mmu-miR-148a*	1.98	1.03E-01	2.53E-01
mmu-miR-1982.1	-2.03	1.03E-01	2.53E-01
mmu-miR-382	-1.88	1.05E-01	2.55E-01
mmu-let-7g	-1.34	1.08E-01	2.55E-01
mmu-miR-340-5p	-1.81	1.09E-01	2.67E-01
mmu-miR-191	1.10	1.10E-01	2.69E-01
mmu-miR-151-5p	-1.86	1.12E-01	2.69E-01
mmu-miR-32	-1.54	1.16E-01	2.71E-01
mmu-miR-20a	-1.67	1.22E-01	2.71E-01
mmu-miR-677	1.40	1.23E-01	2.72E-01
mmu-miR-378	-1.35	1.28E-01	2.77E-01
mmu-miR-671-5p	1.84	1.30E-01	2.78E-01
mmu-miR-450b-3p	-2.12	1.32E-01	2.78E-01
mmu-miR-2137	-1.99	1.32E-01	2.82E-01

mmu-miR-128	-1.29	1.34E-01	2.82E-01
mmu-miR-329	-1.64	1.36E-01	2.89E-01
mmu-miR-34a	-1.33	1.42E-01	2.89E-01
mmu-miR-26b*	1.32	1.47E-01	2.89E-01
mmu-miR-1247	1.82	1.47E-01	2.89E-01
mmu-miR-224	-1.44	1.47E-01	2.91E-01
mmu-miR-409-5p	-1.87	1.49E-01	2.93E-01
mmu-miR-1839-3p	1.35	1.51E-01	2.94E-01
mmu-miR-7a-1*	-1.42	1.52E-01	2.96E-01
mmu-miR-1981	2.21	1.55E-01	2.99E-01
mmu-miR-1195	1.59	1.58E-01	3.03E-01
mmu-miR-203	-1.60	1.60E-01	3.13E-01
mmu-miR-466k	1.79	1.61E-01	3.15E-01
mmu-miR-361	-1.44	1.61E-01	3.19E-01
mmu-miR-146b	-1.81	1.63E-01	3.21E-01
mmu-miR-103-2*	1.70	1.63E-01	3.21E-01
mmu-miR-194	-1.25	1.72E-01	3.21E-01
mmu-miR-542-3p	1.53	1.74E-01	3.26E-01
mmu-miR-700	2.07	1.74E-01	3.39E-01
mmu-miR-15b*	-1.49	1.76E-01	3.42E-01
mmu-miR-214*	-1.39	1.77E-01	3.43E-01
mmu-miR-455	-1.40	1.78E-01	3.43E-01
mmu-miR-337-5p	-1.43	1.82E-01	3.45E-01
mmu-let-7d	-1.36	1.83E-01	3.48E-01
mmu-miR-708*	1.43	1.84E-01	3.54E-01
mmu-miR-667	-1.70	1.87E-01	3.55E-01
mmu-miR-30c-1*	-2.33	1.87E-01	3.55E-01
mmu-miR-186*	-1.86	1.93E-01	3.60E-01
mmu-miR-495	-2.19	1.93E-01	3.60E-01
mmu-miR-322*	-1.63	1.93E-01	3.60E-01
mmu-miR-338-3p	-1.32	1.94E-01	3.61E-01
mmu-miR-490-5p	-1.52	1.96E-01	3.63E-01
mmu-miR-423-5p	-1.21	2.01E-01	3.63E-01
mmu-miR-30a	-1.60	2.04E-01	3.70E-01
mmu-miR-331-3p	-1.54	2.07E-01	3.71E-01
mmu-miR-742	-1.83	2.17E-01	3.71E-01
mmu-miR-804	1.40	2.20E-01	3.79E-01
mmu-miR-195*	-1.26	2.23E-01	3.79E-01
mmu-miR-1964-3p	-2.46	2.24E-01	3.87E-01
mmu-miR-467d	-2.20	2.28E-01	4.16E-01
mmu-miR-672	-1.55	2.38E-01	4.22E-01
mmu-miR-30e	-1.37	2.40E-01	4.23E-01
mmu-miR-1937c	1.84	2.42E-01	4.23E-01
mmu-miR-30a*	-1.23	2.42E-01	4.23E-01
mmu-miR-466f-5p	-1.48	2.45E-01	4.26E-01
mmu-miR-207	-1.83	2.52E-01	4.29E-01

mmu-miR-1951	1.89	2.54E-01	4.31E-01
mmu-miR-29b-1*	-1.49	2.54E-01	4.31E-01
mmu-miR-429	-1.23	2.59E-01	4.33E-01
mmu-let-7a-2*	1.27	2.59E-01	4.36E-01
mmu-miR-22*	-1.13	2.60E-01	4.40E-01
mmu-miR-1957	1.47	2.62E-01	4.54E-01
mmu-miR-376a	-1.93	2.64E-01	4.64E-01
mmu-miR-215	-1.71	2.64E-01	4.65E-01
mmu-miR-181a-1*	1.42	2.70E-01	4.71E-01
mmu-miR-1937a	2.07	2.72E-01	4.73E-01
mmu-miR-7a	-1.15	2.73E-01	4.76E-01
mmu-miR-297a	-1.52	2.80E-01	4.86E-01
mmu-miR-297b-3p	-1.84	2.80E-01	4.86E-01
mmu-let-7e*	1.56	2.87E-01	4.87E-01
mmu-miR-28*	-1.18	3.09E-01	4.87E-01
mmu-miR-98	-1.39	3.14E-01	4.98E-01
mmu-miR-676	-1.47	3.17E-01	5.10E-01
mmu-miR-1937b	1.85	3.17E-01	5.12E-01
mmu-miR-490-3p	-1.46	3.21E-01	5.13E-01
mmu-miR-381	5.10	3.24E-01	5.15E-01
mmu-miR-706	-1.18	3.28E-01	5.23E-01
mmu-miR-744*	1.32	3.29E-01	5.23E-01
mmu-miR-546	-1.94	3.32E-01	5.24E-01
mmu-miR-541	-1.84	3.48E-01	5.25E-01
mmu-miR-16-1*	-1.36	3.57E-01	5.25E-01
mmu-miR-467b*	1.31	3.58E-01	5.26E-01
mmu-miR-2145	-1.20	3.65E-01	5.26E-01
mmu-miR-669i	1.23	3.79E-01	5.27E-01
mmu-miR-18b	-1.50	3.80E-01	5.33E-01
mmu-miR-1274a	1.77	3.83E-01	5.36E-01
mmu-miR-129-2-3p	1.18	3.83E-01	5.36E-01
mmu-let-7f-2*	-1.29	3.92E-01	5.36E-01
mmu-miR-92a-2*	1.35	4.02E-01	5.42E-01
mmu-miR-423-3p	-1.10	4.05E-01	5.46E-01
mmu-miR-200a*	1.06	4.10E-01	5.49E-01
mmu-miR-1839-5p	-1.16	4.18E-01	5.53E-01
mmu-miR-375	-1.36	4.18E-01	5.53E-01
mmu-miR-125b-2-3p	1.28	4.20E-01	5.53E-01
mmu-let-7f-1*	1.22	4.22E-01	5.53E-01
mmu-miR-28	-1.26	4.23E-01	5.56E-01
mmu-miR-1198-5p	-1.21	4.25E-01	5.59E-01
mmu-miR-191*	1.23	4.26E-01	5.63E-01
mmu-miR-30d*	-1.15	4.28E-01	5.63E-01
mmu-miR-194-2*	-1.11	4.33E-01	5.65E-01
mmu-let-7a-1*	-1.17	4.38E-01	5.69E-01
mmu-miR-1934	-1.29	4.40E-01	5.76E-01

mmu-miR-410	-1.35	4.46E-01	5.78E-01
mmu-miR-411*	-1.39	4.50E-01	5.81E-01
mmu-miR-200b*	1.13	4.59E-01	5.89E-01
mmu-miR-466d-3p	-1.33	4.59E-01	5.93E-01
mmu-miR-126-5p	-1.13	4.60E-01	5.98E-01
mmu-miR-200c*	-1.28	4.62E-01	5.98E-01
mmu-miR-291a-5p	-1.36	4.74E-01	6.01E-01
mmu-miR-1935	-1.47	4.74E-01	6.04E-01
mmu-miR-709	-2.72	4.77E-01	6.07E-01
mmu-miR-669l	1.35	4.81E-01	6.13E-01
mmu-miR-27b*	-1.45	4.88E-01	6.15E-01
mmu-miR-34b-3p	-1.35	4.92E-01	6.15E-01
mmu-miR-681	1.51	4.95E-01	6.22E-01
mmu-miR-758	-1.32	5.03E-01	6.23E-01
mmu-miR-192*	1.04	5.08E-01	6.25E-01
mmu-miR-1191	-1.58	5.14E-01	6.28E-01
mmu-miR-1939	1.23	5.14E-01	6.49E-01
mmu-miR-501-5p	1.13	5.22E-01	6.49E-01
mmu-miR-211	-1.30	5.26E-01	6.67E-01
mmu-miR-466h-5p	1.29	5.33E-01	6.83E-01
mmu-let-7g*	1.08	5.35E-01	6.83E-01
mmu-miR-669d	-1.53	5.36E-01	6.94E-01
mmu-miR-297c	-1.25	5.44E-01	6.96E-01
mmu-miR-130b*	-1.14	5.46E-01	6.97E-01
mmu-miR-692	-1.37	5.53E-01	7.01E-01
mmu-miR-376c*	-1.38	5.73E-01	7.01E-01
mmu-miR-1983	1.25	5.75E-01	7.12E-01
mmu-miR-582-3p	1.18	5.92E-01	7.12E-01
mmu-miR-30e*	1.04	6.08E-01	7.22E-01
mmu-miR-491	-1.31	6.09E-01	7.27E-01
mmu-miR-421	-1.12	6.23E-01	7.29E-01
mmu-miR-466f-3p	1.20	6.25E-01	7.37E-01
mmu-miR-467g	-1.25	6.31E-01	7.44E-01
mmu-miR-691	1.37	6.31E-01	7.49E-01
mmu-miR-26a-1*	1.09	6.43E-01	7.49E-01
mmu-miR-135a	-1.21	6.44E-01	7.53E-01
mmu-miR-210	-1.09	6.55E-01	7.53E-01
mmu-miR-212-5p	1.18	6.61E-01	7.53E-01
mmu-miR-1944	-1.08	6.73E-01	7.55E-01
mmu-miR-1929	1.07	6.81E-01	7.92E-01
mmu-miR-141*	1.05	6.86E-01	7.96E-01
mmu-miR-409-3p	1.78	6.88E-01	8.00E-01
mmu-miR-1982*	1.13	6.94E-01	8.00E-01
mmu-let-7a	-1.12	6.95E-01	8.12E-01
mmu-miR-122	-1.19	6.97E-01	8.31E-01
mmu-miR-1186	-1.28	7.00E-01	8.32E-01

mmu-miR-872*	1.06	7.36E-01	8.35E-01
mmu-miR-19b-1*	1.17	7.48E-01	8.43E-01
mmu-miR-379*	-1.22	7.48E-01	8.60E-01
mmu-miR-1249	-1.07	7.61E-01	8.60E-01
mmu-miR-1952	1.27	7.80E-01	8.66E-01
mmu-miR-15a*	1.06	7.83E-01	8.66E-01
mmu-miR-291b-5p	-1.15	7.88E-01	8.66E-01
mmu-miR-872	1.03	7.97E-01	8.77E-01
mmu-miR-30c-2*	-1.07	8.15E-01	8.77E-01
mmu-miR-188-3p	1.16	8.24E-01	8.79E-01
mmu-miR-654-3p	-1.12	8.27E-01	8.81E-01
mmu-miR-103	-1.05	8.39E-01	8.82E-01
mmu-miR-682	-1.12	8.42E-01	8.91E-01
mmu-miR-20b*	1.05	8.46E-01	8.95E-01
mmu-miR-466d-5p	-1.02	8.49E-01	8.98E-01
mmu-miR-193*	-1.12	8.62E-01	9.08E-01
mmu-miR-496	-1.05	8.67E-01	9.21E-01
mmu-miR-154*	1.06	8.73E-01	9.31E-01
mmu-miR-1199	-1.05	8.84E-01	9.36E-01
mmu-miR-10b	-1.05	9.30E-01	9.47E-01
mmu-miR-33*	-1.02	9.34E-01	9.49E-01
mmu-miR-720	1.02	9.59E-01	9.72E-01
mmu-miR-378*	-1.01	9.60E-01	9.72E-01
mmu-miR-802	1.01	9.66E-01	9.76E-01
mmu-miR-712	-1.01	9.69E-01	9.77E-01
mmu-miR-17*	-1.01	9.74E-01	9.80E-01
mmu-miR-671-3p	1.02	9.76E-01	9.80E-01
mmu-miR-467h	-1.01	9.82E-01	9.84E-01
mmu-miR-199b*	1.00	> 0.99	> 0.99

Supplementary Table 4. Gen set enrichment analysis, KEGG and GO Biological process pathways.

Kyoto Encyclopedia of Genes and Genomes (KEEG) pathways				
**Name**	**Hits**	**Genes**	**P-value**	**adjusted P-value**
hsa04110 Cell cycle	6	ORC4; E2F3; CDK6; CDC14B; CCND1; CCND2	0.0029	0.0441
hsa04630 JAK-STAT signaling pathway	6	SOCS5; PDGFRA; OSMR; CCND1; CCND2; IFNAR2	0.0098	0.0441
hsa05206 MicroRNAs in cancer	9	FOXP1; PDGFRA; E2F3; CDK6; PDCD4; CCND1; CCND2; BCL2L2; DICER1	0.0084	0.0441
hsa05202 Transcriptional misregulation in cancer	7	ELK4; CCNT2; RUNX1T1; KMT2A; CCND2; CCNT1; H3F3B	0.0052	0.0441
hsa04218 Cellular senescence	5	RASSF5; E2F3; CDK6; CCND1; CCND2	0.0323	0.0646
hsa05162 Measles	5	CDK6; CCND1; CCND2; CSNK2A1; IFNAR2	0.019	0.0646
hsa04310 Wnt signaling pathway	5	CCND1; CCND2; NKD1; CSNK2A1; ZNRF3	0.0323	0.0646
hsa04934 Cushing syndrome	5	E2F3; CDK6; CCND1; KMT2A; KMT2D	0.0289	0.0646
hsa05160 Hepatitis C	5	E2F3; CDK6; CCND1; PPP2R1B; IFNAR2	0.0289	0.0646
hsa04141 Protein processing in endoplasmic reticulum	5	SEC24A; SSR1; RAD23B; SEC61A2; DNAJC3	0.0368	0.0662
hsa04151 PI3K-Akt signaling pathway	8	COL4A4; PDGFRA; OSMR; CDK6; CCND1; PPP2R1B; CCND2; IFNAR2	0.0426	0.0697
hsa04510 Focal adhesion	5	COL4A4; PDGFRA; VCL; CCND1; CCND2	0.0675	0.0851
hsa05200 Pathways in cancer	10	TPM3; RASSF5; COL4A4; PDGFRA; E2F3; CDK6; RUNX1T1; CCND1; CCND2; IFNAR2	0.0685	0.0851
hsa05169 Epstein-Barr virus infection	5	E2F3; CDK6; CCND1; CCND2; IFNAR2	0.0697	0.0851
hsa05165 Human papillomavirus infection	7	COL4A4; ATP6V1A; CDK6; CCND1; PPP2R1B; CCND2; IFNAR2	0.0709	0.0851
hsa04010 MAPK signaling pathway	5	ELK4; PDGFRA; TAOK1; RPS6KA3; DUSP9	0.2119	0.2384
hsa05168 Herpes simplex virus 1 infection	5	ZNF620; SRPK1; ZNF200; ZNF264; IFNAR2	0.5894	0.6241
hsa01100 Metabolic pathways	12	AZIN2; TRAK2; ATP6V1A; PDE4D; PGM3; SPTLC1; PANK1; KMT2A; ETNK1; KMT2D; ACOX1; PYCR1	0.839	0.839
**GO Biological process pathways**				
**Name**	**Hits**	**Genes**	**P-value**	**adjusted P-value**
GO:0045737 positive regulation of cyclin-dependent protein serinethreonine kinase activity	5	CCNT2; CCNYL1; CCND1; CCND2; CCNT1	0.0	0.0
GO:0006457 protein folding	7	GNAT1; TTC1; PPIL1; CDC37L1; RAD23B; CSNK2A1; FKBP1A	0.0008	0.0077
GO:0007049 cell cycle	9	EPB41; CCNT2; E2F3; PTP4A1; DMTF1; CCNT1; CSNK2A1; SIK1; RPS6KA3	0.0011	0.0077
GO:0016055 Wnt signaling pathway	7	CUL3; CCND1; CSNK1G1; NKD1; RNF138; CSNK2A1; ZNRF3	0.0029	0.0122
GO:0000082 G1S transition of mitotic cell cycle	5	ORC4; USP37; CUL3; CDK6; CCND1	0.0035	0.0122
GO:0006888 ER to Golgi vesicle-mediated transport	7	MCFD2; CUL3; SEC24A; DCTN4; YKT6; TMED2; GOSR1	0.0034	0.0122
GO:0006468 protein phosphorylation	11	MAP3K21; ACVR2A; SRPK1; CDK6; CCND1; CCND2; CCNT1; TAOK1; CSNK2A1; SIK1; CASK	0.0079	0.0237
GO:0006974 cellular response to DNA damage stimulus	7	PARP1; FOXP1; CBX3; CCND1; ZBTB4; TAOK1; ZBTB7A	0.0108	0.0284
GO:0046777 protein autophosphorylation	5	DDR2; MAP3K21; PDGFRA; TAOK1; SIK1	0.0289	0.0674
GO:0015031 protein transport	7	MCFD2; CHMP3; ATG9A; YKT6; VPS33A; VPS53; GOSR1	0.0359	0.0754
GO:0043687 post-translational protein modification	7	FBXO44; SOCS5; CUL3; DCUN1D1; DNAJC3; FEM1B; DCUN1D3	0.0546	0.0838
GO:0043066 negative regulation of apoptotic process	9	SLC9A1; HIGD1A; PDCD4; CCND2; TMBIM4; DNAJC3; BCL2L2; ARHGDIA; RPS6KA3	0.0559	0.0838
GO:0051301 cell division	7	EPB41; CCNT2; USP37; CDK6; CCND1; CCND2; CCNT1	0.0552	0.0838
GO:0008283 cell proliferation	5	GNAT1; CDV3; TBCK; PURB; H3F3B	0.0558	0.0838
GO:0006281 DNA repair	5	CLSPN; PARP1; CDC14B; PAGR1; TAOK1	0.0682	0.0938

GO:0016567 protein ubiquitination	8	SOCS5; CUL3; MSL2; FEM1B; ARIH1; MIB1; RNF138; ZNRF3	0.0715	0.0938
GO:0007283 spermatogenesis	7	AZIN2; ACVR2A; CCNYL1; PGM3; RAD23B; BCL2L2; ACOX1	0.1109	0.137
GO:0019221 cytokine-mediated signaling pathway	5	SOCS5; OSMR; CCND1; NUMBL; IFNAR2	0.1434	0.1634
GO:0006511 ubiquitin-dependent protein catabolic process	5	USP37; CUL3; UBE4A; ARIH1; ZNRF3	0.1478	0.1634
GO:0035556 intracellular signal transduction	6	RASSF5; SOCS5; PRKAR2A; SRPK1; SIK1; RPS6KA3	0.1618	0.1699
GO:0016032 viral process	5	PDGFRA; SRPK1; NUP98; CCNT1; RCOR1	0.2914	0.2914
